# *N*-Arylphenothiazines as strong donors for photoredox catalysis – pushing the frontiers of nucleophilic addition of alcohols to alkenes

**DOI:** 10.3762/bjoc.15.5

**Published:** 2019-01-04

**Authors:** Fabienne Speck, David Rombach, Hans-Achim Wagenknecht

**Affiliations:** 1 Institute of Organic Chemistry, Karlsruhe Institute of Technology (KIT), Fritz-Haber-Weg 6, 76131 Karlsruhe, Germany

**Keywords:** addition, phenothiazine, photochemistry, photoredox catalysis, redox potential

## Abstract

A new range of *N*-phenylphenothiazine derivatives was synthesized as potential photoredox catalysts to broaden the substrate scope for the nucleophilic addition of methanol to styrenes through photoredox catalysis. These *N*-phenylphenothiazines differ by their electron-donating and electron-withdrawing substituents at the phenyl group, covering both, σ and π-type groups, in order to modulate their absorbance and electrochemical characteristics. Among the synthesized compounds, alkylaminylated *N*-phenylphenothiazines were identified to be highly suitable for photoredox catalysis. The dialkylamino substituents of these *N*-phenylphenothiazines shift the estimated excited state reduction potential up to −3.0 V (vs SCE). These highly reducing properties allow the addition of methanol to α-methylstyrene as less-activated substrate for this type of reaction. Without the help of an additive, the reaction conditions were optimized to achieve a quantitative yield for the Markovnivkov-type addition product after 20 h of irradiation.

## Introduction

Visible-light photoredox catalysis has become a precious tool in modern synthetic organic chemistry and experiences a continuously growing interest in industrial applications. The access to electronically excited states of organic molecules allows unlocking new and sometimes complementary chemical reactivities that cannot be tackled by using thermally driven chemical reactions [1]. This complementarity allows for the development of so far unknown transformations [2]. The photochemical reactivity can be tuned by the absorption and excited state characteristics of the photocatalyst. In this context, organic dyes represent a perfectly suited class of photocatalysts as they can easily be modified by the introduction of functional groups that allow fine-tuning the optoelectronic properties of the molecules. Photochemical methods have allowed to overcome some of the current limitations in thermally driven chemistry and to substitute conventional energy demanding chemistry by highly sustainable photochemical methods [3–12].

Phenothiazines have become a precious class of organic molecules, not only due to their widespread use in medicinal chemistry [13] but also because of their fascinating electronic properties. Recently their use in photoredox catalysis allowed for the development of some novel transformations, namely dehalogenation [14] as well as the first pentafluorosulfanylation method starting from sulfur hexafluoride [2]. We are convinced that the value of phenothiazine derivatives in photoredox catalysis is still underestimated. While these compounds found widespread use in ATRA (atom transfer radical addition) polymerization [15–16] the interest of using this class of catalysts only gained limited interest during the last years. The advantage of using *N*-phenylphenothiazine catalysts in photoredox chemistry is attributed to their beneficial redox properties. Moreover, a modification of the core is rather simple and allows fast access to a wide variety of catalysts. Recently it was shown that the radical cation of the photoredox catalyst can play a key role in photoinduced oxidation chemistry [16]. This is rather unusual due to the usually short lifetime of radical cations in solution attributed to their low-lying excited states. Normally, this is the reason why photochemical processes can hardly compete with photophysical decay processes. However, a pre-coordination of the substrate may facilitate electron transfer under non-diffusional controlled conditions. Very recently, the fast (picosecond) excited state dynamics of the radical cation of *N*-phenylphenothiazine was investigated by Wasielewski et al. This radical cation had a high reduction potential of about +2.1 V (vs SCE) [17] allowing the reduction of poorly oxidizing agents. The combination of both properties in one system is a remarkable feature for chemical redox dynamics between −2.1 V up to +2.1 V. One of the key problems in photochemistry, which was recently addressed by the development of the consecutive photoelectron transfer process (conPET) [5], is the need to push the frontiers by accessing high reduction potentials. While the classical photoredox concept is based on the photophysical properties of the excited photoredox catalyst, the idea of the conPET concept mimics nature’s light collection system and consecutively collects the energy of two photons stored in the excited state of the initially pre-promoted photoredox catalyst’s radical ion. This was one of the features to use *N*-phenylphenothiazine for the photoactivation of SF_6_ for the pentafluorosulfanylation of styrenes [2]. This two photon concept can further be extended to the photoredox catalytically generation of hydrated electrons as very powerful reductants (*E* = −2.8 V (vs NHE)) for organic reactions [18].

During the last years, we investigated the photoredox chemistry of new classes of catalysts like perylene bisimides, for their suitability in these types of processes [19] and evaluated the addition of methanol to alkenes as a simple model system. Due to the insufficient reduction potential of the photoredox catalyst, the Markovnikov addition of alcohols through oxidative quenching is yet limited to highly activated, aromatic alkenes. To the best of our knowledge no methods are known today that allow the addition of alcohols to α-methyl-substituted styrenes through photoredox catalysis. The currently available methods are based on a two-step procedure involving an iodoalkoxylation with NIS followed by the reduction of the formed alkyl iodide generating the product in moderate yields [20], or through the direct addition of MeOH catalyzed by either acidic conditions or heated ion exchange resin [21–22]. These methods are therefore not suitable for the alkoxylation of acid or base-labile substrates. To overcome the current limitations of reduction potentials of single electron transfer processes in photoredox catalysis we present herein a range of new *N*-phenylphenothiazine derivatives **1**–**11** as photoredox catalysts. Three of them were identified to be highly suitable for the addition of methanol to alkenes affording the corresponding Markovnikov products.

## Results and Discussion

**Figure 1:**
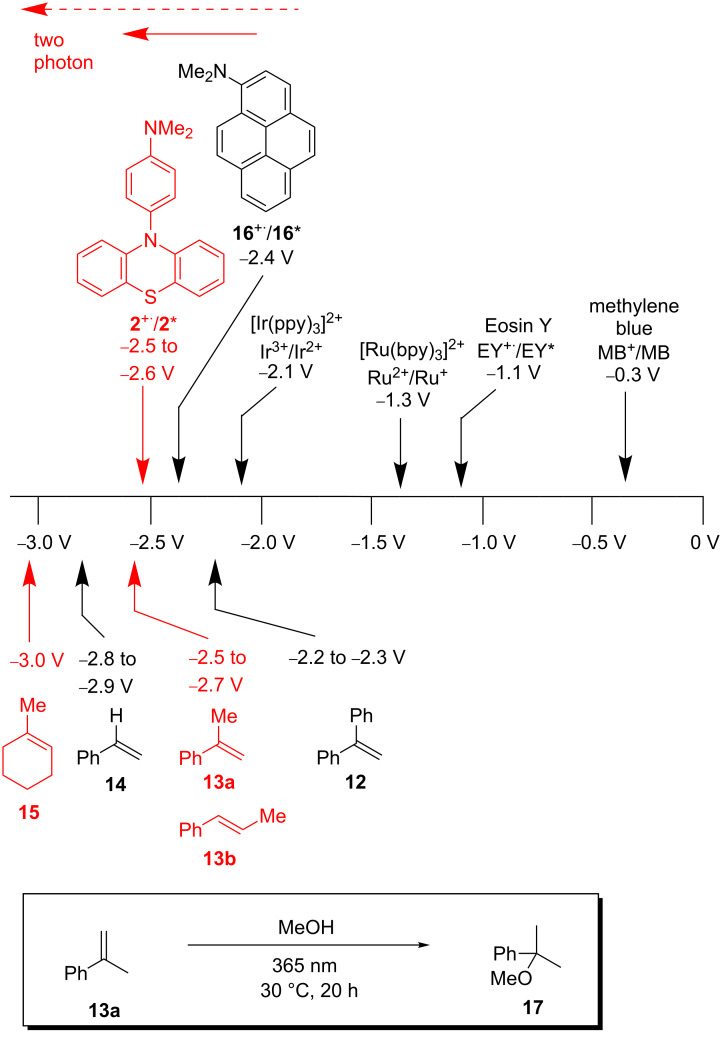
Reduction potentials (vs SCE) of common photoredox catalysts, pyrene **16** and phenothiazine **2**, in comparison to addressable substrate scope **12**–**15**. Bottom: photoredox catalytic addition of MeOH to α-methylstyrene (**13a**).

**Figure 2:**
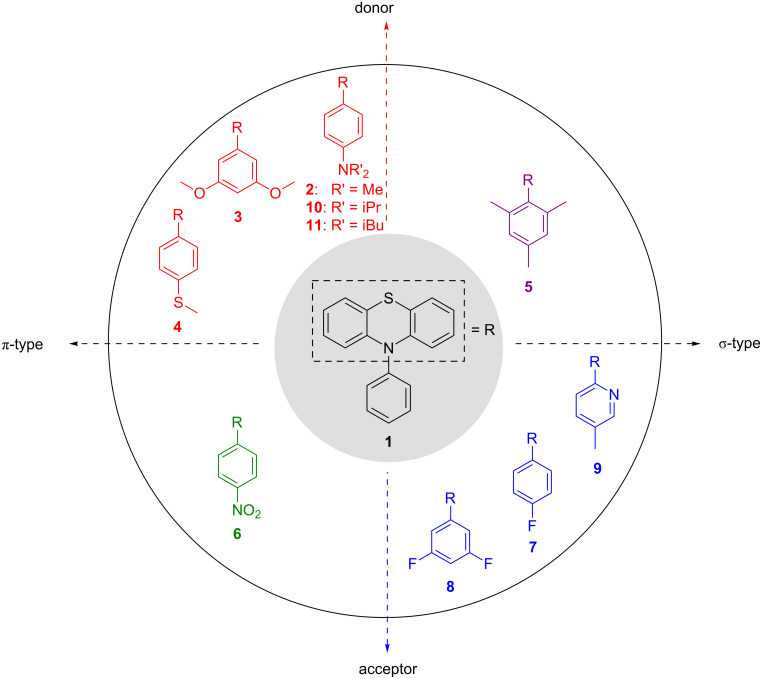
Acceptor or donor-modified phenothiazines **1**–**11** as potential photoredox catalysts.

Activated aromatic olefins, such as 1,1-diphenylethylene (**12**), have reduction potentials *E*_red_(S/S^−^**^·^**) in the range of −2.2 to −2.3 V [23–24], α- and β-methylstyrene (**13a** and **13b**) have an *E*_red_(S/S^−^**^·^**) of −2.5 to −2.7 V [25], and styrene (**14**) an *E*_red_(S/S^−^**^·^**) of −2.6 V (Figure 1) [25–26]. For non-aromatic, alkylated olefins, like 1-methylcyclohex-1-ene (**15**), the reduction potentials are estimated to values of *E*_red_(S/S^−^**^·^**) = −3.0 V [25]. In our initial photoredox catalyst screening [26], we identified 1-(*N*,*N*-dimethylamino)pyrene (**16**) having an excited-state reduction potential E*_ox_(P^+^**^·^**/P*) of −2.4 V (determined by cyclic voltammetry and *E*_00_). Thus, we are able to photoreduce 1,1-diphenylethylene (**12**), but not yet α-methylstyrene (**13**), and clearly not non-aromatic (alkylated) olefins, such as methylated cyclohexene **15**, as basic structures. The absorption of *N*-phenylphenothiazine (**1**) disappears at around 390 nm. This feature requires the excitation of the molecule using UV light sources and contradicts the use of visible light irradiation due to vanishing extinction coefficients at the edge to the visible region. To reach for high excited state reduction potentials and excite at rather long irradiation wavelengths an energetically high lying electronic groundstate potential has to be connected with a small S_0_–S_1_ gap for the development of strongly reducing photoredox catalysts. Thus, we first focused our strategy in catalyst development on the synthesis of some highly electron-rich phenothiazines **2**–**5** as well as some electron-deficient phenothiazines **6**–**9** to analyze the influences of modifications of the core and the aryl moiety (Figure 2). The observed trends allowed us to come up with a set of strongly reducing photoredox catalysts that operate under UV-A conditions close to the visible range. In order to extend the scope of available reduction potentials we expected that the *N*-phenylphenothiazine core having installed additional electron-donating groups, like NR_2_ in **2**, reaches very low reduction potentials in the range of *E*(P^+^**^·^**/P*) = −2.5 to −3.0 V which is in the range of solid sodium [27], that would be able to attack low-substituted styrenes like **13a** and **13b**.

**Figure 3:**
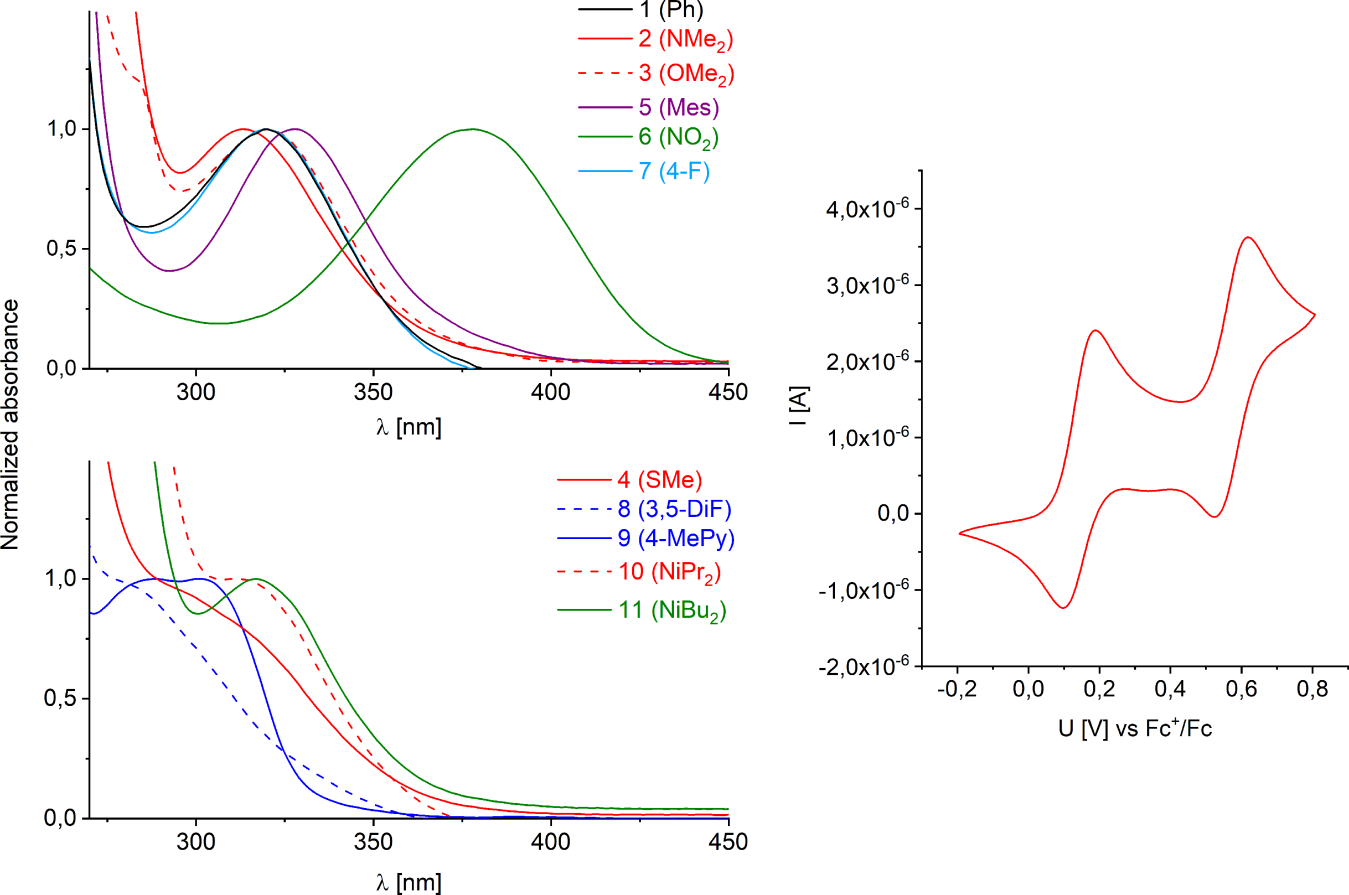
Normalized UV–vis absorption spectra above 290 nm of *N*-phenylphenothiazines **1**–**11** (left) and representative cyclic voltammogram of **2** (right).

Firstly, the absorbance characteristics of the derivatives **1**–**9** were analyzed and compared (Figure 3). The parent compound *N*-phenylphenothiazine (**1**) shows an absorption maximum at 320 nm. Substitution of the arene moiety results in a shift of the absorption maxima due to a change in the HOMO–LUMO gaps. It turned out that the introduction of the π-donating dimethylamino substituent in **2** induces a hypsochromic shift of the absorption maximum by 7 nm to 313 nm, while the mesityl group present in **5** as a σ-donor causes a bathochromic shift of about 8 nm. The detailed structure of the alkyl group attached to the amino part in the phenothiazines **2**, **10** and **11** showed no significant change in the absorption maximum of the S_1_ transition (**10** in comparison to **2**), but replacement of the methyl groups by branched isobutyl groups in **11** resulted in a hypsochromic shift of the bathochromic features of absorption. The nitro compound **6** turned out to show a distinct long wavelength absorption that is apart from the region of absorption of all other catalysts by a shift of about 40 nm which is probably due to a charge transfer state. Interestingly, the spectrum of the methylpyridine derivative **9** showed a rather short absorption maximum at 302 nm.

All *N*-phenylphenothiazines **1**–**11** were additionally characterized by cyclic voltammetry (Figure 3 and Table 1) [28]. We found the first oxidation half wave of the unsubstituted *N*-phenylphenothiazine (**1**) as a fully reversible process as it was described in literature recently [18]. The second oxidation process of **1** is almost reversible but induces to some extent an irreversible oxidation process that shows up as further reduction half wave in the cyclic voltammogram. This is true for almost all synthesized derivatives **3**–**9**. The radical dication is known to undergo disproportionation reactions [27], which potentially explain the results. Only the amino derivatives **2**, **10** and **11** managed to undergo a second completely reversible oxidation process. To exclude interference with water and oxygen the measurements were carried under strict exclusion of any contaminants. The first oxidation of the lead structure **1** was found to occur at *E*(**1**^+^**^·^**/**1**) = 0.75 V (vs SCE). The substitution of the arene moiety by one (see **7**) or two fluorine substituents (see **8**) only leads to a shift in the reduction potential of about 0.06 V. This trend was expected due to the lower electron density of these two *N*-phenylphenothiazines at the arene moiety. However, the effect by the pure σ-acceptor fluorine is not very pronounced. In the case of the 4-NO_2_ substituted derivative **6** the pronounced influence of the π-acceptor shifts the reduction potential to a value of up to *E*(**6**^+^**^·^**/**6**) = 0.89 V (vs SCE). Substitution of the *N*-aryl moiety by electron-donating substituents shifts the potentials correspondingly towards lower reduction potentials, as expected. By introducing the thioether substituent (see **4**) to the arene the potential drops to about *E*(**4**^+·^/**4**) = 0.71 V (vs SCE). If the steric bulk is enhanced by a mesityl substituent (see **5**) the reduction potential interestingly is higher than in the parent compound **1** although there are electron-donating alkyl groups present in the molecular structure. This can be explained by a twist of the arene moieties due to steric bulk causing an interruption of the delocalization. The electron transfer is found to occur at *E*(**5**^+·^/**5**) = 0.67 V (vs SCE). However, the introduction of the π-donating dimethylamino substituent dramatically shifts the reduction potential to up to *E*(**2**^+·^/**2**) = 0.57 V (vs SCE). We hypothesized that the introduction of even more donating substituents could reduce the reduction potential further. Therefore, we synthesized the modified alkylated compounds **10** and **11**, respectively. Indeed, both compounds showed a lower potential with **11** having an *E*_red_(**11**^+^**^·^**/**11**) = 0.49 V (vs SCE). This made us curious about the excited state potential of the catalysts. Using the Rehm–Weller equation (without considering the solvent term) [29] we estimated the excited state potential of these catalysts to be as low as *E*_red_(**X**^+^**^·^**/X*) ≈ −2.9 to −3.0 V (vs SCE).

**Table 1:** Reduction potentials *E*_red_(**X**^+^**^·^**/**X**) of *N*-phenylphenothiazines **1**–**11** (determined by cyclic voltammetry using ferrocene as standard).

compound	*E*_1_(**X**^+·^/**X**)^a^	*E*_2_(**X**^+·^/**X**)	*E*_1_(**X**^+·^/X*)	*E*_00_^b^

**1**	0.75 V	1.50 V^c^	−2.5 V	3.2 eV
**2**	0.57 V	1.00 V	−2.5 V	3.1 eV
**3**	0.73 V	1.49 V	−2.5 V	3.2 eV
**4**	0.71 V	–	−3.0 V	3.7 eV^d^
**5**	0.67 V	1.59 V	−2.5 V	3.1 eV
**6**	0.89 V	1.55 V	−2.1 V	3.0 eV
**7**	0.75 V	1.50 V^c^	−2.5 V	3.3 eV
**8**	0.77 V	1.05 V^c^	−2.6 V	3.4 eV
**9**	0.80 V	–	−2.6 V	3.4 eV
**10**	0.53 V	0.98 V	−2.9 V	3.4 eV
**11**	0.49 V	0.96 V	−2.9 V	3.4 eV

^a^Converted from the ferrocene scale to the scale vs SCE: +0.38 V [30]. ^b^*E*_00_ was estimated by using the method of determination of the intersection of the normalized absorption and fluorescence. ^c^Irreversible. ^d^Fluorescence in the UV-A range, see Figure S27 (Supporting Information File 1).

**Figure 4:**
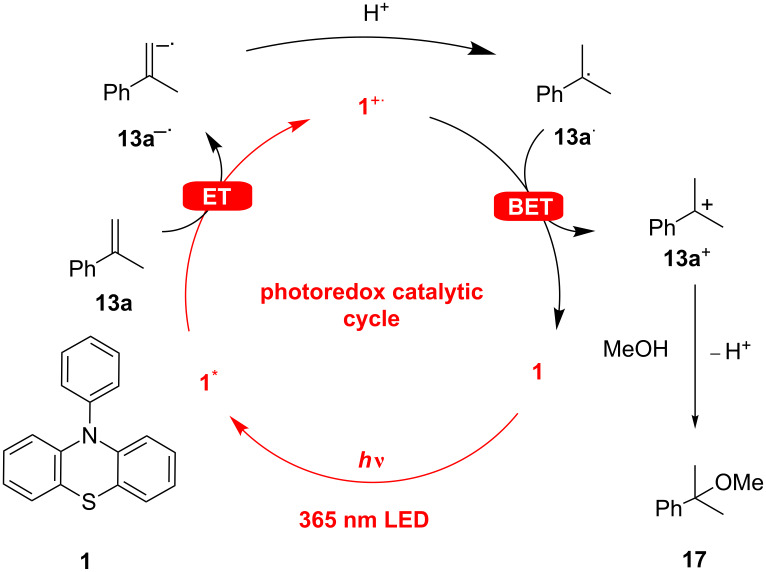
Proposed mechanism for the photoredox-catalyzed addition of methanol to α-methylstyrene (**13a**). (ET = electron transfer, BET = back-electron transfer).

The proposed photoredox catalytic mechanism (Figure 4) for the nucleophilic addition of methanol to olefins starts with photoinduced electron transfer from the *N*-phenylphenothiazine (**1**) as photocatalyst to **13a** as substrate. The resulting substrate radical anion **13a**^−·^ is instantaneously protonated to radical **13a**^·^ and back-electron transfer to the intermediate phenothiazine radical cation **1**^+·^ yields the substrate cation **13a**^+^. The latter is attacked by methanol as nucleophile and finally deprotonation gives rise to the product **17** (see Figure 4). The principal problem of this type of photoredox catalytic cycle is that the back-electron transfer cannot compete with the initial electron transfer because both components, **1**^+·^ and **13**^−·^, are formed only in stationary low concentrations. In the past, we used electron mediators as additives (triethylamine) [19,26] or peptides with substrate-binding sites [31–32] to overcome this problem. For the current work, we propose a radical ion pair in a solvent cage that undergoes an extremely fast proton transfer followed by the intracage back-electron transfer, since triethylamine is no longer needed (vide infra).

The evaluation of both the optical and electrochemical properties of the prepared phenothiazine derivatives **1**–**11** leads to the conclusion that only the dialkylamino derivatives **2**, **10** and **11** come up with an estimated excited state reduction potential capable of reducing α-methylstyrene (**13a**). The optoelectronic properties and the excited state reduction potential of *E*_red_(**2**^+·^/2*) = −2.5 V of dimethylamino compound **2** that is close to the reduction potential of the substrate **13a** encouraged us to approach the so far not yet observed addition of methanol to this less activated substrate promoted by catalyst **2**. After optimizing our catalytic system with catalyst **2**, we could also confirm the expected reactivity of the branched dialkylamino-substituted derivatives **10** and **11** (entry 12 and 13, Table 2).

**Table 2:** Screening of reaction conditions for the methanol addition to α-methylstyrene (**13a**).^a^

entry	**13a** [mM]	catalyst	mol %	additive	solvent	yield

1	84.6	**2**	5	NEt_3_^b^	MeOH	31%
4	84.6	**2**	5	–	MeOH	84%
5	42.3	**2**	10	–	MeOH	quant.
6	42,3	**2**	10	–	MeOH	–^c^
7	42.3	**2**	10	–	MeOH	–^d^
8	42.3	–	–	NEt_3_	MeOH	–^d^
9	42.3	**2**	10	–	MeOD	78%^e^
11	170	**2**	10	–	MeOD	quant.^e^
12	170	**10**	10	–	MeOH	quant.^e^
13	170	**11**	10	–	MeOH	quant.^e^

^a^Conditions: 30 °C, 65 h, 365 nm LEDs. ^b^10 equiv. ^c^No light. ^d^No catalyst. ^e^20 h.

The initial conditions included irradiation of substrate **13a** in the presence of the catalyst (5 mol %) in methanol and triethylamine (10% (v/v)) as the additive according to our previously reported photoredox catalysis with pyrene **16** [18]. Under these conditions the product **17** was formed in a yield of 31%. It turned out that omitting the additive as electron shuttle enhanced the catalytic efficiency and the yield increased up to 84%. Obviously, this is a major difference between the photoredox catalysis with pyrene **16**, where triethylamine was absolutely crucial to obtaining good product yields, and *N*-phenylphenothiazine **1**. Having this electron shuttle (ca. 1 M) in the reaction mixture efficiently leads to silent or non-silent quenching of the reactive species due to the following modes of quenching. While the back-electron transfer under generation of the triethylamine radical cation unproductively consumes electrons while oxidizing triethylamine, the hydrogen abstraction pathway generates the reduced phenylethane, which is observed in small concentrations in the reaction mixture. The analysis of the reaction mixture still showed some unreacted starting material. Assuming the first electron transfer as the rate-determining step the substrate concentration was reduced to 42 mM and the catalyst concentration was increased to 10 mol %. This change in the reaction conditions led to a quantitative product formation after 65 h. Finally, the rather long reaction times were addressed by speeding up the reaction simply by raising the concentration of all components to 170 mM. This reduced the reaction time to 20 h irradiation producing the product **17** in quantitative yield. However, a further increase of substrate concentration slowed down the reaction again by speeding up silent electron transfer processes.

## Conclusion

One of the major current challenges in photoredox catalysis is the design of chromophores suitable for the most reductive chemical reactions, like for instance reductions by alkaline metals, affording reaction conditions that are easier to handle. While solid sodium comes up with a reduction potential of −3.0 V (vs SCE) the present novel *N*-phenylphenothiazine-based photoredox catalysts reach impressive excited state reduction potentials with up to −3.0 V (vs SCE) in case of catalyst **10**. We applied the strongly reducing *N*-phenylphenothiazines **2**, **10** and **11** for the photoredox catalytic reduction of α-methylstyrene (**13a**) as a less activated styrene that could not be addressed before. After optimization, the photoredox catalytic addition of methanol proceeded in quantitative yield within 20 h without any further additive, like triethylamine as electron shuttle. We could speed up the reaction by using increased concentrations of the substrate and the catalyst affording the product in quantitative yield after 20 h reaction time. We believe that photoredox catalysis with synthetically easily accessible *N*-phenylphenothiazines will lead to the development of new photoredox catalytic approaches based on their strongly reducing excited states.

## Supporting Information

File 1 Copies of ^1^H and ^13^C NMR spectra, mass spectra, absorption and emission spectra and cyclic voltammetry data of **1**–**11** and **17**.
