# Supplementary material for: N-Arylphenothiazines as strong donors for photoredox catalysis – pushing the frontiers of nucleophilic addition of alcohols to alkenes
Source: Beilstein J Org Chem. 2019 Jan 4;15:52–9. doi: 10.3762/bjoc.15.5 (PMC6334793; doi:10.3762/bjoc.15.5)
Supplement: File 1 — Copies of 1H and 13C NMR spectra, mass spectra, absorption and emission spectra and cyclic voltammetry data of 1–11 and 17. [file Beilstein_J_Org_Chem-15-52-s001.pdf]

BEILSTEIN JOURNAL OF ORGANIC CHEMISTRY

Supporting Information

for

N-Arylphenothiazines as strong donors for photoredox catalysis – pushing the frontiers of nucleophilic addition of alcohols to alkenes

Fabienne Speck, David Rombach and Hans-Achim Wagenknecht

Beilstein J. Org. Chem. **2019,** *15,* 52–59. doi:10.3762/bjoc.15.5

Copies of ^{1}H and ^{13}C NMR spectra, mass spectra, absorption and emission spectra and cyclic voltammetry data of 1–11 and 17

License and Terms: This is a supporting information file under the terms of the Creative Commons Attribution License (http://creativecommons.org/licenses/by/4.0). Please note that the reuse, redistribution and reproduction in particular requires that the authors and source are credited.
The license is subject to the *Beilstein Journal of Organic Chemistry* terms and conditions: (https://www.beilstein-journals.org/bjoc)

Materials and methods

All chemicals were purchased from Sigma Aldrich, Fluka, Alfa Aesar, ABCR or Tokyo Chemical Industry (TCI) and used as received if not otherwise stated. Benchmark experiments for addition of methanol to α-methylsytrene were performed using Fischer HPLC grade solvent.

NMR spectroscopic data were recorded using the following spectrometer hardware. Bruker B-ACS 60, ^{1}H NMR (300 MHz), ^{13}C NMR (75 MHz); Bruker Avance DRX 400, ^{1}H NMR (400 MHz), ^{13}C NMR (101 MHz), ^{19}F NMR (376 MHz); Bruker Ascend 500, ^{1}H NMR (500 MHz), ^{13}C NMR (126 MHz), ^{19}F NMR (470 MHz). Chemical shifts of the ^{1}H ,^{13}C and ^{19}F NMR spectra are reported in parts per million (ppm) relative to the solvent as an internal standard and converted to the TMS-reference system by applying frequency correction using the values of Fulmer et al. [S1]. Routine ^{13}C NMR spectroscopy was recorded while applying broadband ^{1}H-decoupling. The chemical shifts of ^{19}F NMR experiments are reported relative to CCl_3F as standard, which was added in a concentration of 0.1% to the neat NMR solvent. Coupling constants (*J*) are given in hertz (Hz) and the multiplicity of signals is reported as follows: s (singlet), d (doublet), t (triplet), q (quadruplet), p (pentet), sext (sextet), m (multiplet), br. s (broad singlet), dt (doublet of triplets), td (triplet of doublets), dp (doublet of pentets), ddp (doublet of doublet of pentets). Analytical GC determination of the yields of the catalysis was carried out with a Bruker 430 GC instrument equipped with a capillary column FactorFourTM VF-5 ms (30 m × 0.25 mm × 0.25 µm), using flame ionization detection. The oven temperature program was: initial temperature 95 °C, hold for 1 min, ramp at 15 °C/min to 220 °C, hold for 4 min, ramp at 15 °C/min to 325 °C, hold for 2 min. The injector transfer line temperature was set

to 220 °C. Measurements were performed in split–split mode using hydrogen as the carrier gas (flow rate 30 mL/min).

For the calculation of the GC yields a concentration series with (2-methoxypropan-2-yl)benzene (**17**) and 2-bromotoluol (8.77 µmol/mL) as internal standard was prepared. The R-value of the series was determined to be 0.91. To analyze the catalysis mixture, 100 µL of the crude reaction mixture, 174.6 µL of a stock solution of 2-bromotoluol (8.77 µmol/mL) and 724.6 µL ethyl acetate were mixed and shaken vigorously. Then, a volume of 300 µL of the resulting solution was transferred to an GC vial.

Irradiation of the photochemical reactions was carried out using a setup which was designed and manufactured by the University of Regensburg and the workshop of the Institute for Physical Chemistry at KIT Karlsruhe. We warmly thank Dieter Waltz and Klaus Stree for their kind support with manufacturing the irradiation hardware. A Nichia NVSU233A LED was applied for 365 nm irradiation and the photoreactions were irradiated from the bottom. The temperature during the reaction time was controlled using a LAUDA Alpha R8 thermostat. High resolution mass spectrometry was performed on a Finnigan Modell MAT 95 using an electron impact ionization source or on a Q Exactive Plus Orbitrap from Thermo Scientific. MALDI mass spectra were recorded on a Bruker Daltonics Biflex-IV spectrometer, operated in linear negative mode with 3-hydroxy-2-pyridinecarboxylic acid as a matrix. GC–MS coupling was recorded using a Varian 431 GC with a capillary column FactorFourTM VF-5 ms (30 m × 0.25 mm × 0.25 µm) and a Varian 210 ion trap mass detector. Thin layer chromatography was performed using Fluka silica gel 60 F254 coated aluminium foil. Flash chromatography was carried out on silica gel 60 supplied by Sigma Aldrich (43–60 µm. Spectroelectrochemical measurements were carried out using a Varian Cary 50 spectrometer at room temperature and a HORBIA-Scientific

Fluoromax-4 spectrofluorometer with an AC 200 thermostat from Thermo Scientific and the FluoroEssence software V3.5 in semi-micro quartz glass cuvettes (width 1 cm, volume 1.4 mL) from Starna.

The cyclic voltammetry measurements were carried out with complete exclusion of air and moisture. We warmly thank Prof. Dr. Frank Breher for sharing his CV infrastructure. The working electrode and the counter electrode were made of platinum. Reference electrode serving as the potential zero point was made of silver. For the electrolysis solution, a 0.05 M solution of tetrabutylammonium hexafluorophosphate in dry DCM or in dry MeCN was prepared.

Photochemical experiment setup

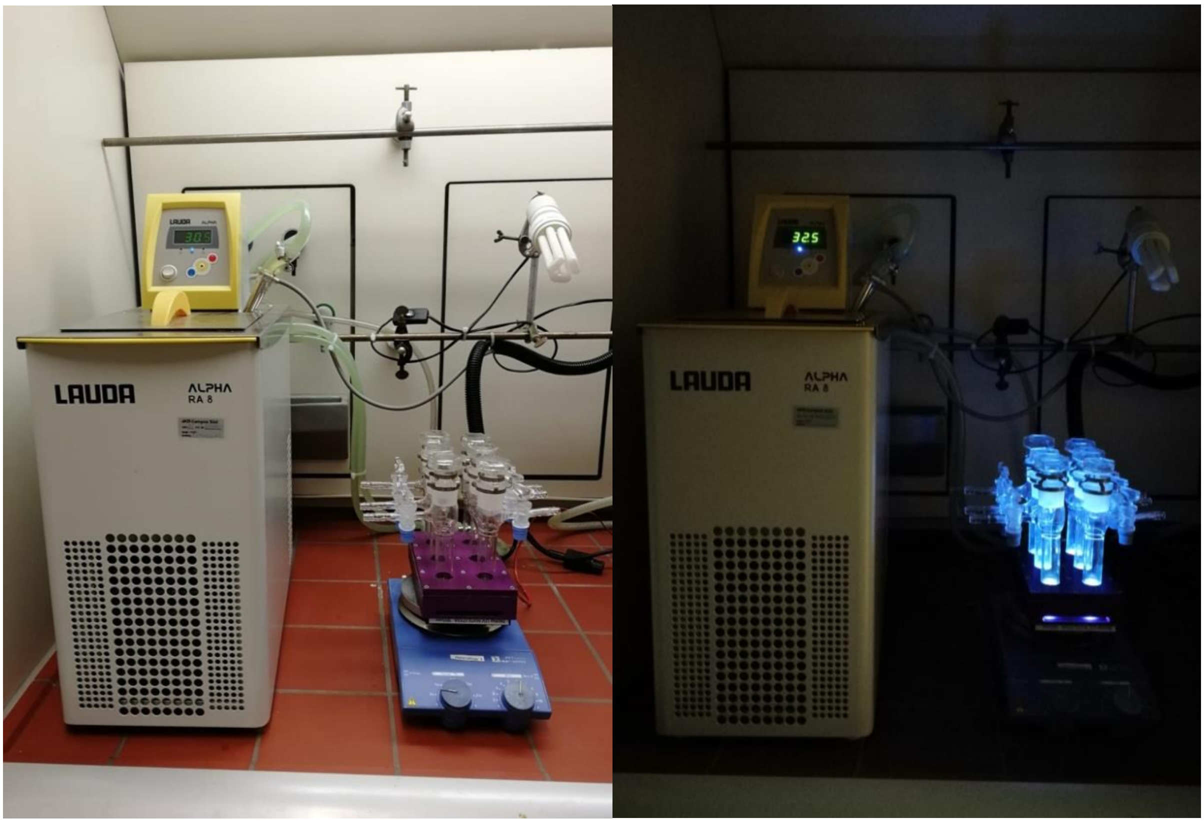

Figure S1: Modular LED irradiation setup and cooling blocks.

Synthetic procedures

General procedure A - synthesis of *N*-phenylphenothiazines. *N*-phenylpheno-thiazines were synthesized similarily to the reported procedure [S2]. Phenothiazine (1.00 equiv) was dissolved in anhydrous toluene (0.51 M). Bromobenzene (1.22 equiv), KO*t*-Bu (1.29 equiv) and $(t\text{-Bu})_3PHBF_4$ (6 mol %) were added, followed by Pd_2dba_3 (3 mol % as 6 mol % Pd). The reaction mixture was degassed using three freeze-pump-thaw cycles and finally stirred under reflux for 20 °h. After reaching room temperature, 100 mL EtOAc and 50 mL water were added to the reaction mixture. After phase separation, the aqueous phase was extracted additionally with 3 × 100 mL EtOAc. The combined organic phases were dried over Na_2SO_4, the solvent evaporated and the crude product purified by column chromatography.

General procedure B - synthesis of *N*-phenylphenothiazines. *N*-Phenothiazine (1.00 equiv), aryl halide (1.50 equvi), sodium *tert*-butoxide (2.50 equiv), Pd_2dba_3 (0.05 equiv/5 mol %) and tricyclohexylphosphine (0.07 equiv) were dissolved in anhydrous toluene (0.26 M). The reaction mixture was then stirred under reflux under inert atmosphere overnight. The reaction mixture was cooled to room temperature and 50 mL water were added. The reaction mixture was extracted with EtOAc (3 × 100 mL). The combined organic phases were dried over $MgSO_4$, the solvent removed and the crude product purified by column chromatography.

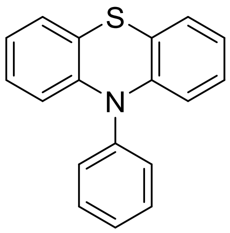

Synthesis of 1

10-Phenyl-10*H*-phenothiazine was synthesized similarily to the reported procedure [S2]. Phenothiazine (810 mg, 4.06 mmol, 1.00 equiv), bromobenzene (520 µL, 780 mg, 4.97 mmol, 1.22 equiv), KO*t*-Bu (587 mg, 5.23 mmol, 1.29 equiv), (*t*-Bu)$_3$PHBF$_4$ (71 mg, 0.244 mmol, 6 mol %) and Pd$_2$dba$_3$ (112 mg, 0.122 mmol, 6 mol % Pd) were used for the preparation following general procedure A and purified by column chromatography (cyclohexane, silica gel, R_f = 0.33). The product was obtained as colorless solid (1.08 g, 3.93 mmol, 97%) and analytical data were identical with those in the literature [S3].

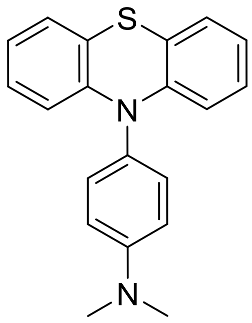

Synthesis of 2

Phenothiazine (750 mg, 3.80 mmol, 1.00 equiv), 4-bromo-*N*,*N*-dimethylaniline (1.14 g, 5.70 mmol, 1.50 equiv), NaO*t*-Bu (913 mg, 9.50 mmol, 2.50 equiv), tricyclohexylphosphine (73 mg, 0.26 mmol, 0.07 equiv) and Pd_2dba_3 (173 mg, 0.19 mmol, 0.05 equiv) were used for the preparation following general procedure B and purified by column chromatography (hexane/CH_2Cl_2 4:1, silica gel, R_f = 0.14). The product was obtained as white solid (229.9 mg, 0.722 mmol, 19%)**:** ^{1}H NMR (300 MHz, THF-d_8): δ (ppm) = 7.17 (d, J = 8.7 Hz, 2H), 6.95 - 6.89 (m, 4H), 6.80 - 6.68 (m, 4H), 6.22 (d, J = 8.1 Hz, 2H), 3.03 (s, 6H)**:** ^{13}C NMR (126 MHz, $CDCl_3$): δ (ppm) = 150.3, 145.4, 131.9, 129.4, 127.2, 126.9, 122.4, 119.8, 116.1, 114.1, 40.6. MALDI-TOF-MS: m/z (%) = 318.7 [M^+].

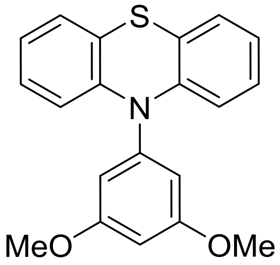

Synthesis of 3

Phenothiazine (750 mg, 3.80 mmol, 1.00 equiv), bromodimethoxybenzene (1.24 g, 5.70 mmol, 1.50 equiv), NaO*t*-Bu (910 mg, 9.50 mmol, 2.50 equiv), tricyclohexylphosphine (75 mg, 0.26 mmol, 0.07 equiv) and Pd_2dba_3 (170 mg, 0.19 mmol, 0.05 equiv) were used for the preparation following general procedure B and purified by column chromatography (hexane/CH_2Cl_2 5:1, silica gel, R_f = 0.19). The product was isolated as light yellow powder (1.08 g, 3.22 mmol, 85%)**:** ^{1}H NMR (300 MHz, THF-d_8): δ (ppm) = 6.96 8d, *J* = 7.4 Hz, 2H), 6.86 – 6.74 (m, 4H), 6.61 (s, 1H), 6.57 (s, 6H), 6.33 (d, *J* = 8.0 Hz, 2H), 3.79 (s, 6H)**:** ^{13}C NMR (126 MHz, $CDCl_3$): δ (ppm) = 162.5, 143.9, 127.1, 126.8, 122.7, 120.3, 116.3, 108.0, 105.5, 100.5, 55.6. MALDI-TOF-MS : m/z (%) = 335.9 [M^+].

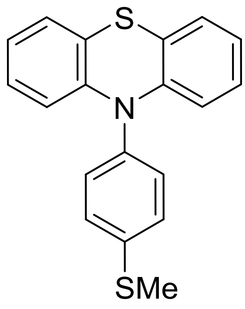

Synthesis of 4

Phenothiazine (1.00 g, 5.00 mmol, 1.00 equiv), 4-bromothioanisol (2,20 mL, 1.50 g, 7.50 mmol, 1.50 equiv), NaO*t*-Bu (1.20 g, 12.5 mmol, 2.50 equiv), tricyclohexylphosphine (100 mg, 0.35 mmol, 0.07 equiv) and Pd_2dba_3 (230 mg, 0.25 mmol, 0.05 equiv) were used for the preparation following general procedure B and purified by column chromatography (hexane/CH_2Cl_2 5:1, silica gel, R_f = 0.20). The product was isolated as colorless powder (1.44 g, 4.48 mmol, 90%)**:** ^{1}H NMR (500 MHz, THF-d_8): δ (ppm) = 7.50 (d, *J* = 8.6 Hz, 2H), 7.32 (d, *J* = 8.6 Hz, 2H), 6.97 (d, *J* = 7.3 Hz, 2H), 6.86 – 6.72 (m, 4H), 6.22 (d, *J* = 7.3 Hz, 2H), 2.55 (s, 3H)**:** ^{13}C NMR (126 MHz, $CDCl_3$): δ (ppm) = 139.0, 137.6, 135.2, 131.6, 128.2, 127.6, 127.2, 126.9, 116.0, 15.7. MALDI-TOF-MS: m/z (%) = 319.8 [M^+].

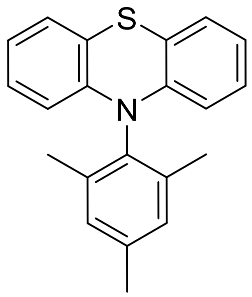

Synthesis of 5

Phenothiazine (750 mg, 3.80 mmol, 1.00 equiv), 2-bromomesitylene (0.870 mL, 1.13 g, 5.70 mmol, 1.50 equiv), NaO*t*-Bu (910 mg, 9.50 mmol, 2.50 equiv), tricyclohexylphosphine (75 mg, 0.26 mmol, 0.07 equiv) and Pd_2dba_3 (170 mg, 0.19 mmol, 0.05 equiv) were used for the preparation following general procedure B and purified by column chromatography (hexane, silica gel, R_f = 0.20). The product was obtained as colorless solid (340 mg, 0.874 mmol, 23%)**:** ^{1}H NMR (300 MHz, THF-d_8): δ (ppm) = 7.10 (s, 2H), 6.85 (d, J = 7.0 Hz, 2H), 6.75 – 6.65 (m, 4H), 2.35 (s, 3H), 2.13 (s, 6H)**:** ^{13}C NMR (126 MHz, $CDCl_3$): δ (ppm) = 141.2, 138.0, 134.6, 130.1, 128.7, 127.0, 126.2, 121.8, 118.2, 113.8, 21.0, 17.9. MALDI-TOF-MS: m/z (%) = 317.1 [M^+].

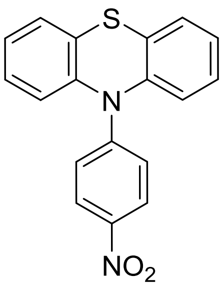

Synthesis of 6

[S4]. Phenothiazine (829 mg, 4.16 mmol, 1.00 equiv), 4-bromonitrobenzene (1.26 g, 6.24 mmol, 1.50 equiv), NaO*t*-Bu (1.00 g, 10.4 mmol, 2.50 equiv), tricyclohexylphosphine (82.0 mg, 0.29 mmol, 0.07 equiv) and Pd_2dba_3 (192 mg, 0.21 mmol, 0.05 equiv) were used for the preparation following general procedure B and purified by column chromatography (hexane/CH_2Cl_2 2:1, silica gel, R_f = 0.14). The product was isolated as yellow powder (595 mg, 1.86 mmol, 45%)**:** ^1H NMR (500 MHz, $CDCl_3$): δ (ppm) = 8.05 (d, *J* = 9.3 Hz, 2H), 7.51 (d, *J* = 7.7 Hz, 2H), 7.46 (d, *J* = 7.7 Hz, 2H), 7.39 (t, *J* = 7.5 Hz, 2H), 7.27 (d, *J* = 7.7 Hz, 2H), 7.02 (d, *J* = 9.3 Hz, 2H)**:** ^{13}C NMR (126 MHz, $CDCl_3$): δ (ppm) = 151.2, 141.1, 140.6, 134.8, 129.3, 127.7, 127.2, 126.9, 125.7, 113.9. HR-EI-MS m/z (calc.) = 320.0619 [M^+]; m/z (found) = 321.0689 [$M+H^+$].

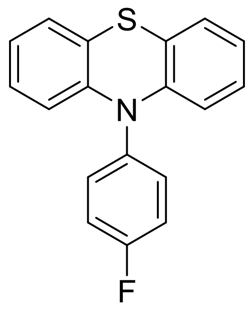

Synthesis of 7

Phenothiazine (810 mg, 4.06 mmol, 1.00 equiv), 1-bromo-4-fluorobenzene (875 mg, 549 µL, 4.97 mmol, 1.22 equiv), KO*t*-Bu (587 mg, 5.23 mmol, 1.29 equiv), (*t*-Bu)$_3$PHBF$_4$ (71 mg, 0.244 mmol, 6 mol %) and Pd$_2$dba$_3$ (112 mg, 0.122 mmol, 6 mol % Pd) were used for the preparation following general procedure A and purified by column chromatography (cyclohexane, silica gel, R_f = 0.40). The product was obtained as colorless solid (1.10 g, 3.75 mmol, 92%)**:** ^{1}H NMR (acetonitrile-d_3, 300 MHz): δ 7.49 – 7.32 (m, 4H), 7.04 (dd, *J* = 7.6, 1.7 Hz, 2H), 6.91 (ddd, *J* = 8.4, 7.4, 1.7 Hz, 2H), 6.84 (td, *J* = 7.4, 1.3 Hz, 2H), 6.20 (dd, *J* = 8.2, 1.3 Hz, 2H)**:** ^{13}C NMR (126 MHz, acetonitrile-d_3) δ 163.1 (d, *J* = 245.8 Hz), 145.1 , 137.9 (d, *J* = 3.1 Hz), 133.8 (d, *J* = 8.9 Hz), 128.2 , 127.6 , 123.7 , 120.8 , 118.7 (d, *J* = 22.8 Hz), 117.0. ^{19}F NMR (471 MHz, acetonitrile-d_3) δ -113.3 – -113.4 (m, 1F). HR-EI-MS m/z (calc.) = 293.0674 [M$^+$]; m/z (found) = 293.0676 [M$^+$].

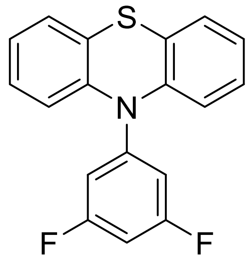

Synthesis of 8

Phenothiazine (810 mg, 4.06 mmol, 1.00 equiv), 1-bromo-3,5-difluorobenzene (575 µL, 964 mg, 4.97 mmol, 1.22 equiv), KO*t*-Bu (587 mg, 5.23 mmol, 1.29 equiv), (t-Bu)$_3$PHBF$_4$ (71 mg, 0.244 mmol, 6 mol %) and Pd$_2$dba$_3$ (112 mg, 0.122 mmol, 6 mol % Pd) were used for the preparation following general procedure A. The product was obtained as colorless solid (976 mg, 3.13 mmol, 77%)**:** ^{1}H NMR (500 MHz, acetonitrile-d^3): δ 7.32 (dd, *J* = 7.7, 1.5 Hz, 2H), 7.21 (td, *J* = 7.8, 1.5 Hz, 2H), 7.11 (td, *J* = 7.6, 1.4 Hz, 2H), 6.96 (dd, *J* = 8.1, 1.3 Hz, 2H), 6.81 – 6.69 (m, 3H)**:** ^{13}C NMR (126 MHz, acetonitrile-d_3) δ 165.00 (dd, *J* = 245.8, 15.4 Hz), 147.64 (t, *J* = 12.6 Hz), 142.95, 128.8, 128.7, 128.5, 126.03, 123.29 , 106.3-106.1 (m), 100.25 (t, *J* = 26.3 Hz). ^{19}F NMR (471 MHz, acetonitrile-d_3) δ -108.36 – -108.69 (m, 2F). HR-EI-MS: m/z (calc.) = 311.0580 [M$^+$]; m/z (found) = 311.0579 [M$^+$].

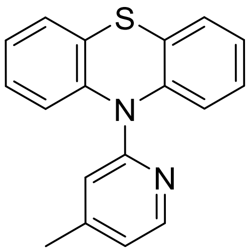

Synthesis of 9

Phenothiazine (750 mg, 3.80 mmol, 1.00 equiv), 2-bromo-4-methylpyridine (0.64 mL, 981 mg, 5.70 mmol, 1.50 equiv), NaO*t*-Bu (910 mg, 9.50 mmol, 2.50 equiv), tricyclohexylphosphine (75 mg, 0.26 mmol, 0.07 equiv) and Pd_2dba_3 (170 mg, 0.19 mmol, 0.05 equiv) were used for the preparation following general procedure B and purified by column chromatography (hexane/CH_2Cl_2 1:1, silica gel, R_f = 0.20). The product was isolated as colorless powder (939 mg, 3.23 mmol, 86%)**:** ^{1}H NMR (500 MHz, $CDCl_3$): δ (ppm) = 8.20 (d, *J* = 5.1 Hz, 1H), 7.48 (d, *J* = 8.0 Hz, 2H), 7.36 (dd, *J* = 7.7 Hz, 2H), 7.26 (t, *J* = 7.7 Hz, 2H), 7.13 (t, *J* = 7.5 Hz, 2H), 6.79 (s, 1H), 6.76 (d, *J* = 5.1 Hz, 1H), 2.24 (s, 3H)**:** ^{13}C NMR (126 MHz, $CDCl_3$): δ (ppm) = 156.5, 149.4, 147.9, 141.8, 131.6, 128.1, 127.0, 125.9, 125.4, 119.1, 112.4, 21.4. MALDI-TOF-MS : m/z (%) = 290.9 [M^+].

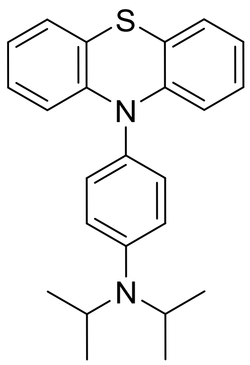

Synthesis of 10

Phenothiazine (518 mg, 2.60 mmol, 1.00 equiv), 4-bromo-*N*,*N*-diisopropylaniline (**19**, 0.71 mL, 1.00 g, 3.90 mmol, 1.50 equiv), NaO*t*-Bu (625 mg, 6.50 mmol, 2.50 equiv), tricyclohexylphosphine (51.0 mg, 0.18 mmol, 0.07 equiv) and Pd_2dba_3 (119 mg, 0.13 mmol, 0.05 equiv) were used for the preparation following general procedure B and purified by column chromatography (hexane/ethylacetate 50:1, silica gel, R_f = 0.33). The product was isolated as colorless powder (740 mg, 1.98 mmol, 76%)**:** ^{1}H NMR (500 MHz, $CDCl_3$): δ (ppm) = 7.15 (d, *J* = 8.8 Hz, 2H), 7.01 (d, *J* = 8.7 Hz, 2H), 6.98 (d, *J* = 7.5 Hz, 2H), 6.85 (t, *J* = 6.8 Hz, 2H), 6.77 (t, *J* = 7.3 Hz, 2H), 6.30 (d, *J* = 8.0 Hz, 2H), 3.89 (m, 2H), 1.32 (d, *J* = 7.5 Hz, 12H)**:** ^{13}C NMR (126 MHz, $CDCl_3$): δ (ppm) = 148.0, 145.2, 131.1, 129.5, 126.9, 126.6, 122.0, 119.6, 118.6, 115.9. MALDI-TOF-MS : m/z (%) = 372.90 [M^+].

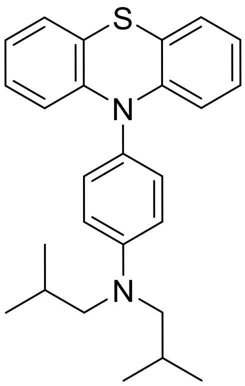

Synthesis of 11

Phenothiazine (795 mg, 3.99 mmol, 1.00 equiv), 4-bromo-*N*,*N*-diisobutylaniline (**20**, 1.70 g, 5.98 mmol, 1.50 equiv), NaO*t*-Bu (959 mg, 9.98 mmol, 2.50 equiv), tricyclohexylphosphine (78.5 mg, 0.28 mmol, 0.07 equiv) and Pd_2dba_3 (183 mg, 0.20 mmol, 0.05 equiv) were used for the preparation following general procedure B and purified by column chromatography (hexane, silica gel, R_f = 0.23). The product was isolated as colorless powder (853 mg, 2.12 mmol, 57%)**:** ^{1}H NMR (500 MHz, $CDCl_3$): δ (ppm) = 7.13 (d, *J* = 8.0 Hz, 2H), 6.97 (d, *J* = 7.4 Hz, 2H), 6.84 (t, *J* = 7.2 Hz, 2H), 6.76 (t, *J* = 7.2 Hz, 2H), 6.27 (d, *J* = 8.0 Hz, 2H), 3.21 (d, *J* = 7.0 Hz, 4H), 2.20 – 2.12 (hept, *J* = 6.7 Hz, 2H), 0.96 (d, *J* = 6.7 Hz, 12H)**:** ^{13}C NMR (126 MHz, $CDCl_3$): δ (ppm) = 147.9, 145.3, 131.5, 127.9, 126.9, 126.6, 122.0, 119.5, 115.9, 113.7, 60.7, 26.5, 20.5. HR-EI-MS: m/z (calc.) = 402.2130 [M^+]; m/z (found) = 402.2120 [M^+].

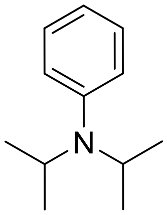

Synthesis of 18

[S5]. A mixture of bromobenzene (3.40 mL, 4.71 g, 30.0 mmol), potassium *tert*-butoxide (5.05 g, 45.0 mmol, 1.50 equiv), diisopropylamine (6.30 mL, 4.55 g, 45 mmol, 1.50 equiv), Pd(dppf)Cl_2 (50 mg, 68.3 µmol, 0.002 equiv) and 40 mL DMSO was degassed (× 3) and heated to 150 °C for 24 h. After cooling to room temperature, the mixture was poured into brine solution (100 mL), then extracted with diethyl ether (2 × 100 mL). The organic layer was separated, dried over $MgSO_4$ and evaporated. The brown colored residue was suspended into hexanes (100 mL) and extracted with 5% aqueous HCl solution (2 × 100 mL). The aqueous layer was made basic by adding NaOH pellets at 0 °C, then extracted with diethyl ether (2 × 100 mL). The separated organic layer was dried over $MgSO_4$ and evaporated to yield an orange oil (1.12 g, 6.32 mmol, 21%)**:** ^{1}H NMR (500 MHz, $CDCl_3$): δ (ppm) = 7.20 (t, *J* = 7.8 Hz, 2H), 6.90 (d, *J* = 8.5 Hz, 2H), 6.77 (t, *J* = 7.3 Hz, 1H), 3.77 (hept, *J* = 6.6 Hz, 2H), 1.21 (d, *J* = 6.6 Hz, 12H). HR-EI-MS: m/z (calc.) = 177.1517 [M^+]; m/z (found) = 178.1588 [$M+H^+$].

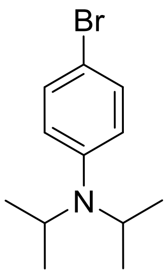

Synthesis of 19

[S5]. To a solution of **18** (1.10 mL, 1.00 g, 5.64 mmol, 1.00 equiv) in 15 mL of DMF at −10 °C was added dropwise a solution of NBS (1.05 g, 5.92 mmol, 1.05 equiv) in 10 mL of DMF. The mixture was stirred at −10 °C for 1 h. The mixture was diluted with brine (100 mL) and extracted with ethyl acetate (3 × 100 mL). The combined organic layers were dried over $MgSO_4$ and evaporated. The residue was redissolved into diethyl ether (75 mL) and filtered. The filtrate was washed with NaOH (3 M, 100 mL) and dried over $MgSO_4$. The solvent was removed to give a yellow oil (1.34 g, 5.25 mmol, 93%)**:** ^{1}H NMR (300 MHz, $CDCl_3$): δ (ppm) = 7.25 (d, J = 8.8 Hz, 2H), 6.74 (d, J = 9.1 Hz, 2H), 3.73 (hept, J = 6.7 Hz, 2H), 1.19 (d, J = 6.9 Hz, 12H).

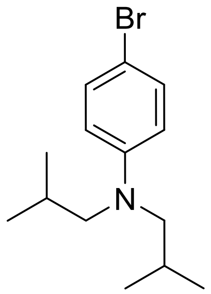

Synthesis of 20

[S5]. To a solution of *N,N*-diisobutylaniline (1.67 mL, 1.50 g, 7.30 mmol, 1.00 equiv) in 20 mL of DMF at −10 °C was added dropwise a solution of NBS (1.37 g, 7.67 mmol, 1.05 equiv) in 10 mL of DMF. The mixture was stirred at −10 °C for 40 min. The mixture was diluted with brine (100 mL) and extracted with ethyl acetate (3 × 100 mL). The combined organic layers were dried over $MgSO_4$ and evaporated. The residue was redissolved into diethyl ether (75 mL) and filtered. The filtrate was washed with NaOH (3 M, 100 mL) and dried over $MgSO_4$. The solvent was removed to give a light yellow powder (1.91 g, 6.72 mmol, 92%)**:** ^{1}H NMR (500 MHz, $CDCl_3$): δ (ppm) = 7.24 (d, *J* = 8.7 Hz, 2H), 6.50 (d, *J* = 8.7 Hz, 2H), 3.11 (d, *J* = 7.5 Hz, 4H), 2.04 (hept, *J* = 6.7 Hz, 2H), 0.88 (d, *J* = 6.7 Hz, 12H)**:** ^{13}C NMR (126 MHz, $CDCl_3$): δ (ppm) = 147.2, 131.8, 114.2, 106.9, 60.5, 26.4, 20.4. HR-EI-MS: m/z (calc.) = 283.0936 [M^+]; m/z (found) = 284.1003 [$M+H^+$].

Spectroscopic data

UV–vis absorption

The UV–vis absorption spectra were recorded at 20 °C in acetonitrile (HPLC grade, Fisher Scientific) or dichloromethane (HPLC grade, Fisher Scientific) using slit 2 nm.

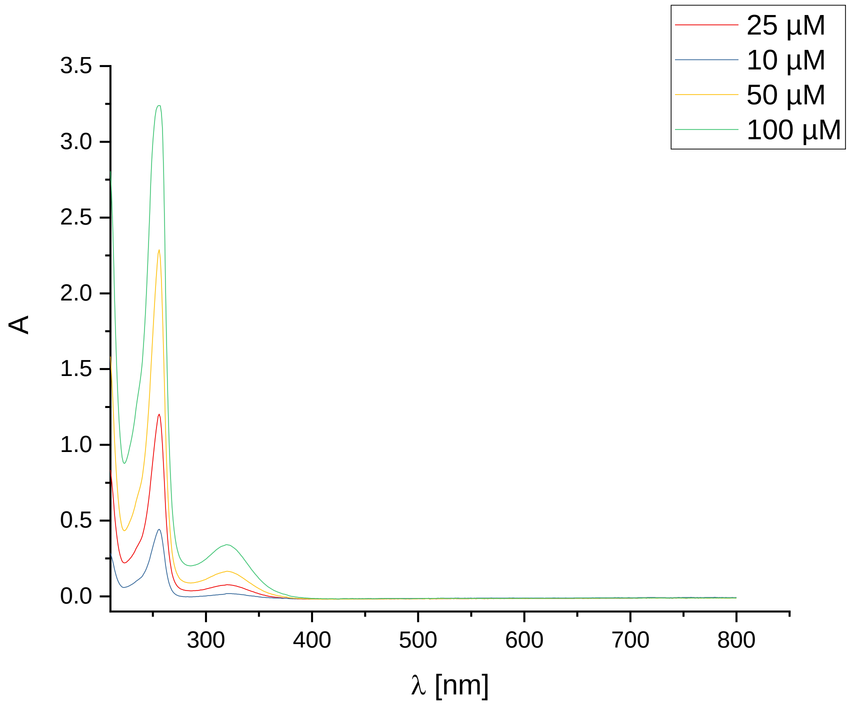

Figure S2: UV–vis absorption spectrum of **1** in MeCN.

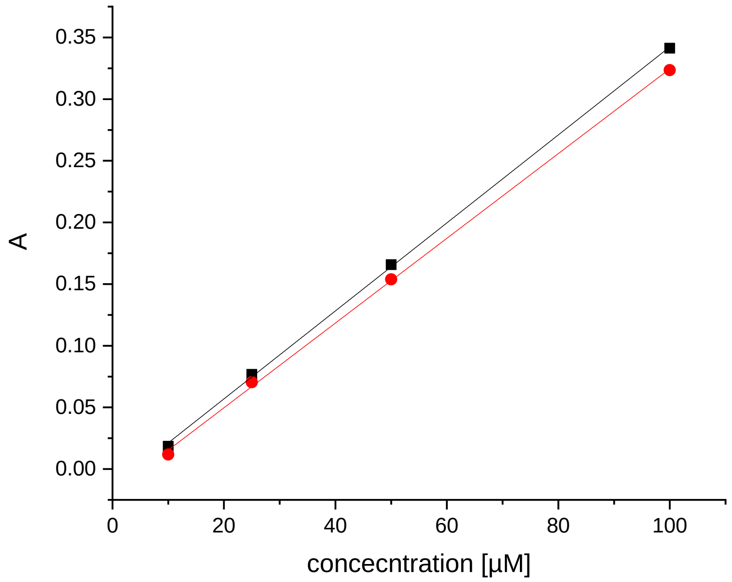

Figure S3: Concentration-dependent UV–vis absorption at 313 nm and 320 nm for determination of the extinction coefficient of 10-phenyl-10*H*-phenothiazine (**1**) in MeCN.

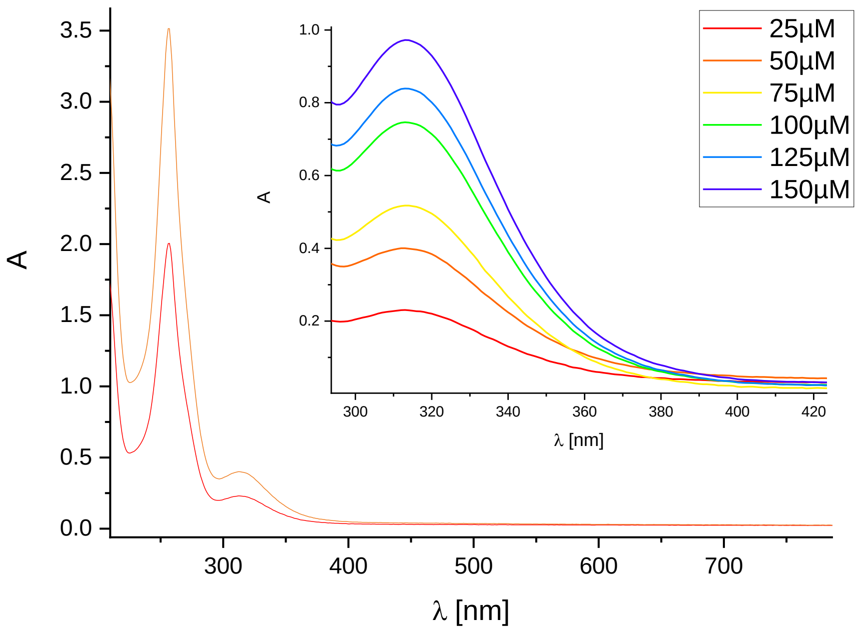

Figure S4: UV–vis absorption spectra of **2** in CH_2Cl_2.

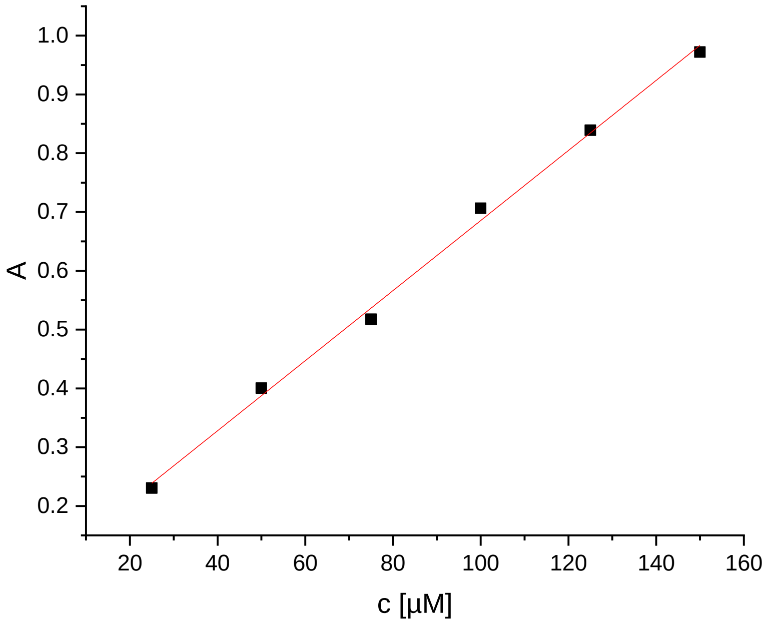

Figure S5: Concentration-dependent UV–vis absorption for determination of extinction coefficient of **2** in CH_2Cl_2.

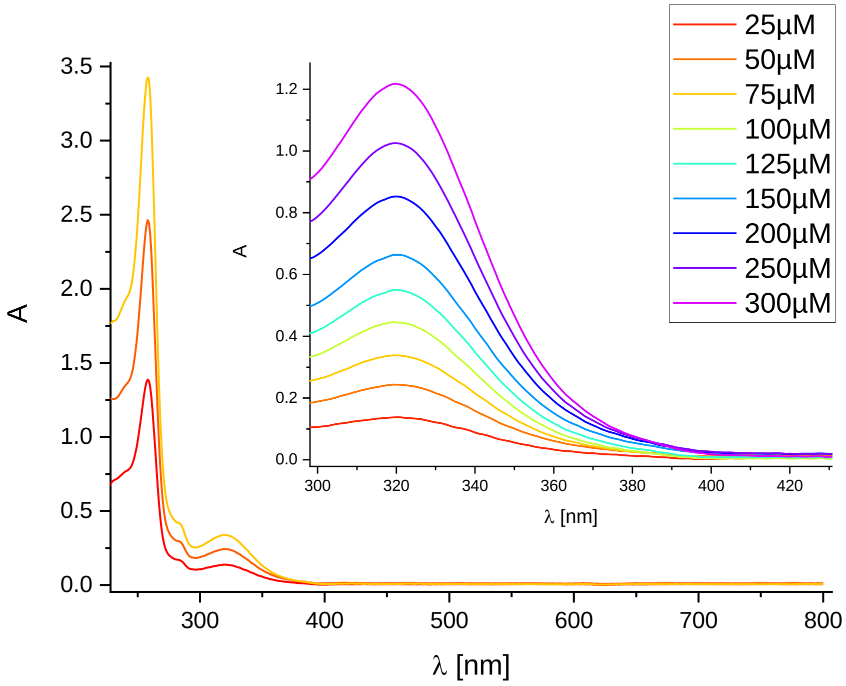

Figure S6: UV–vis absorption spectra of **3** in CH_2Cl_2.

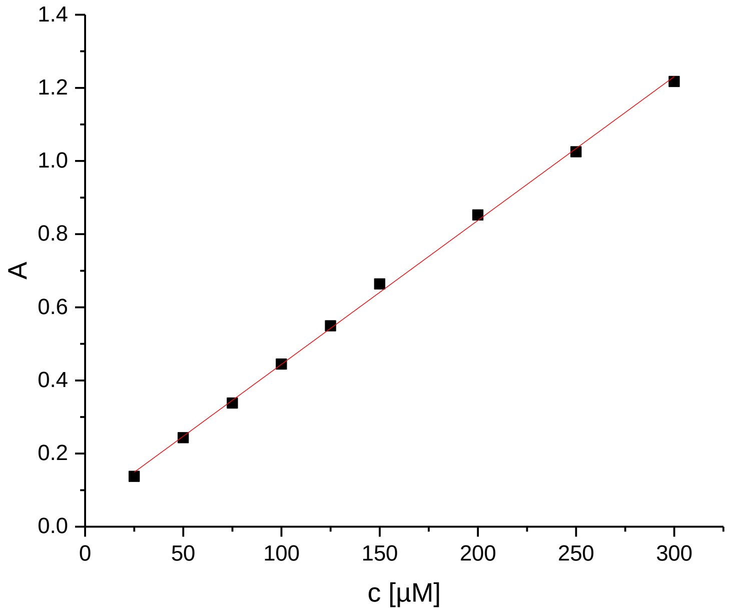

Figure S7: Concentration-dependent UV–vis absorption for determination of extinction coefficient **3** in CH_2Cl_2.

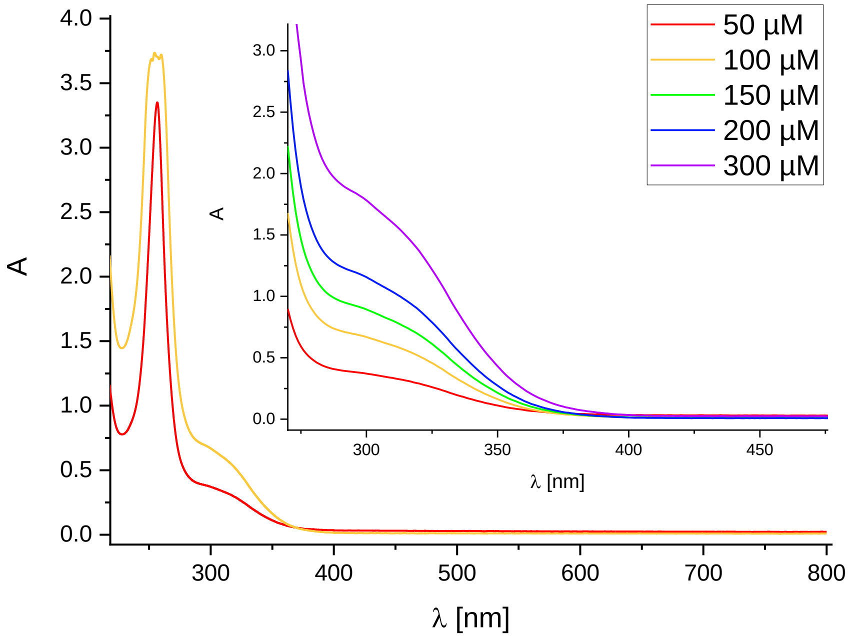

Figure S8: UV–vis absorption spectra of **4** in CH_2Cl_2.

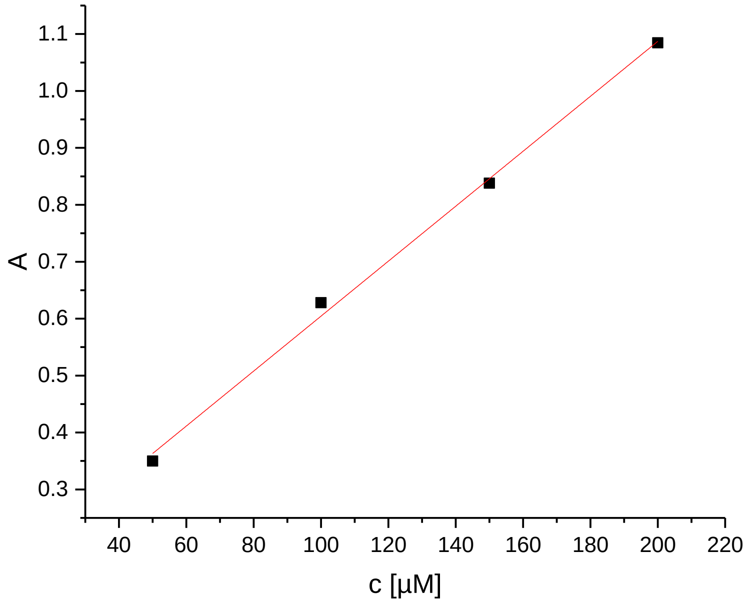

Figure S9: Concentration-dependent UV–vis absorption for determination of extinction coefficient of **4** in CH_2Cl_2.

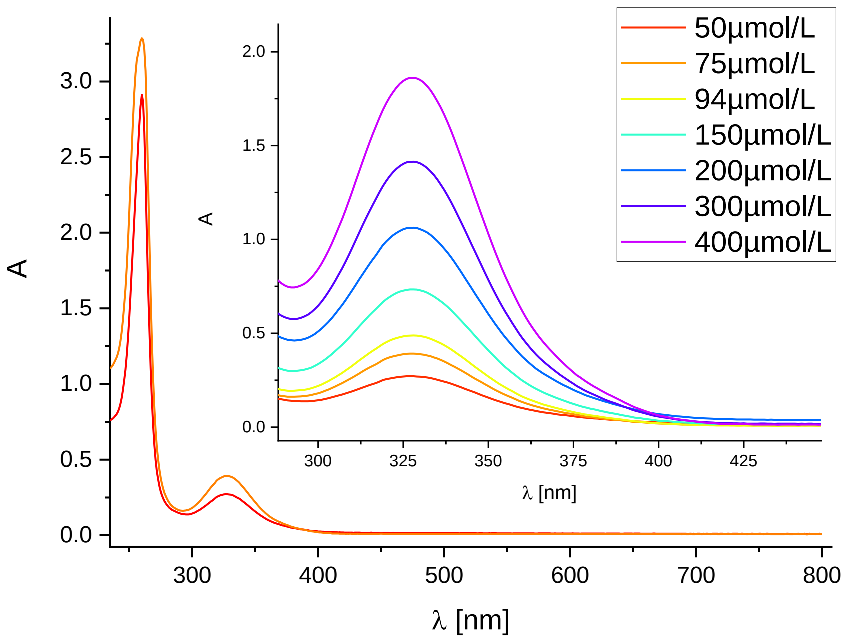

Figure S10: UV–vis absorption spectra of **5** in CH_2Cl_2.

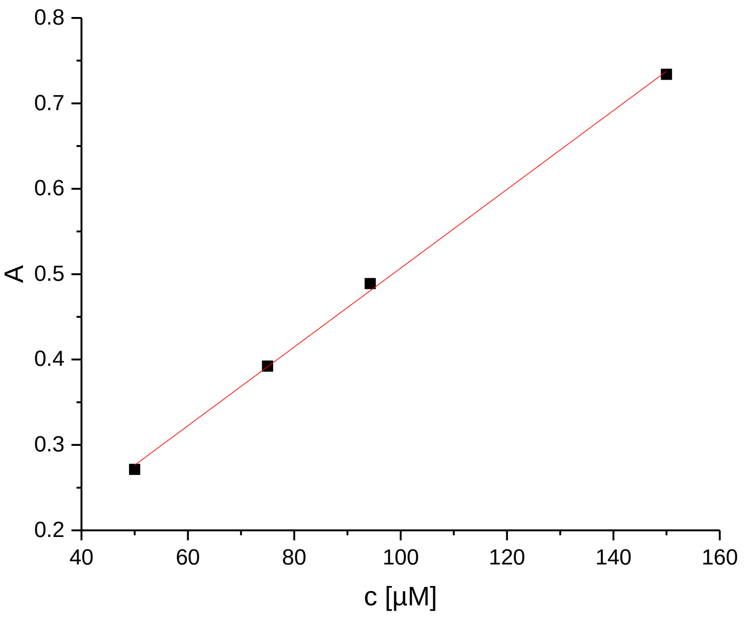

Figure S11: Concentration-dependent UV–vis absorption for determination of extinction coefficient of **5** in CH_2Cl_2.

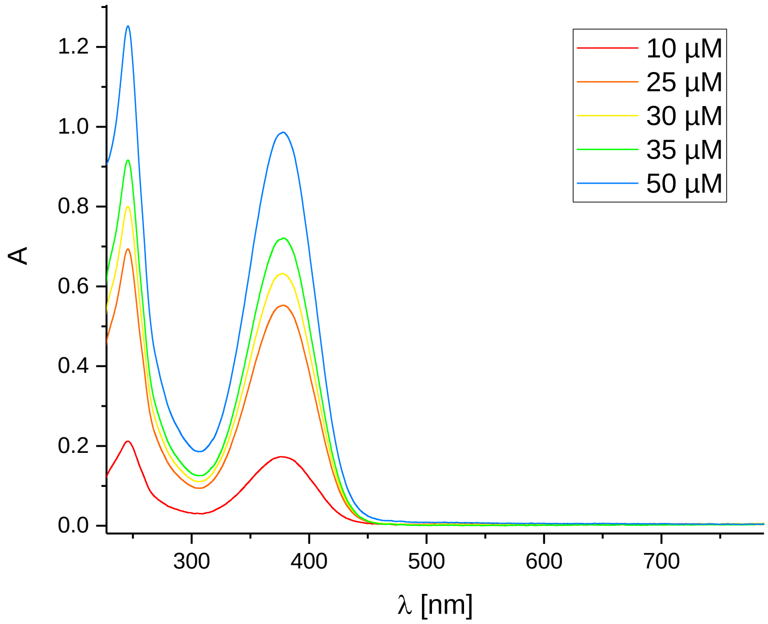

Figure S12: UV–vis absorption spectra of **6** in CH_2Cl_2.

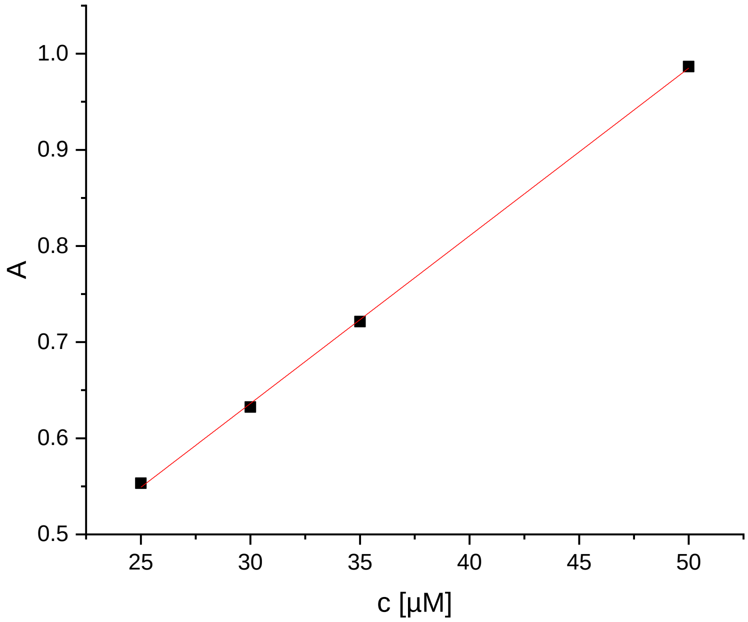

Figure S13: Concentration-dependent UV–vis absorption for determination of extinction coefficient of **6** in CH_2Cl_2.

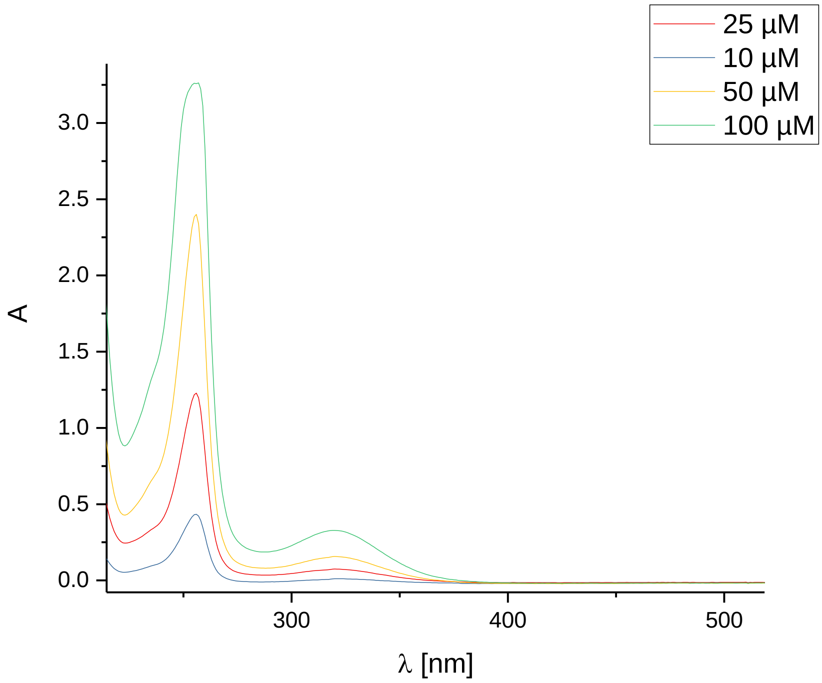

Figure S14: UV–vis absorption spectra of **7** in MeCN.

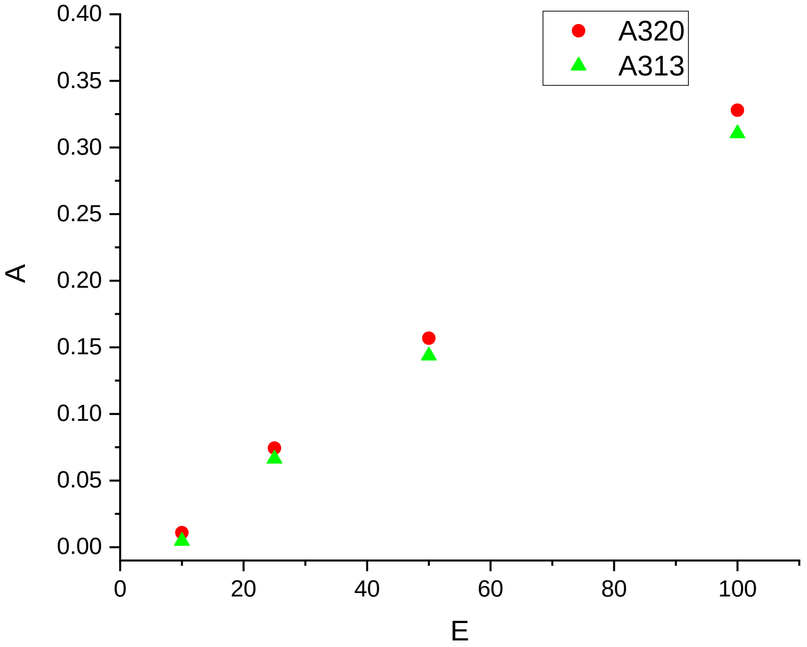

Figure S15: Concentration-dependent UV–vis absorption for determination of extinction coefficient of **7** in MeCN.

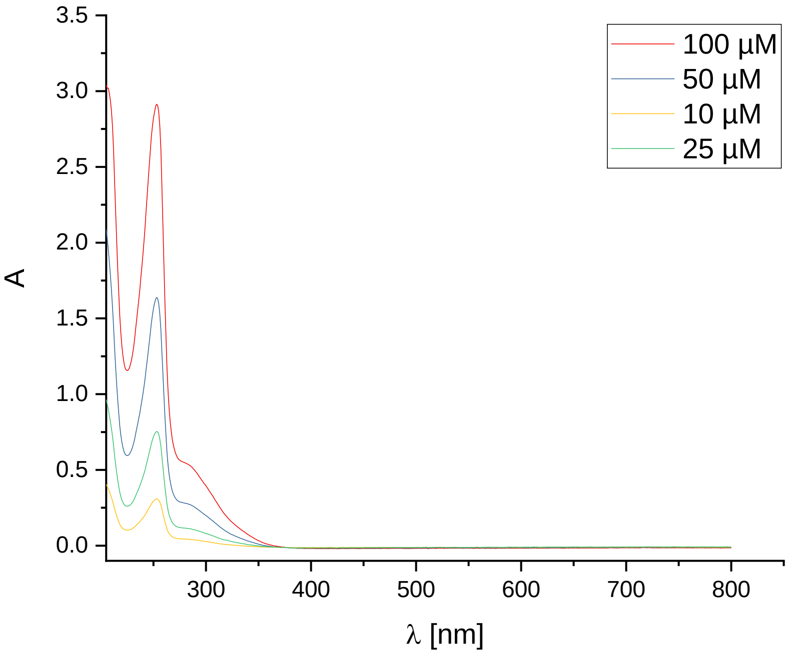

Figure S16: UV–vis absorption spectra of **8** in MeCN.

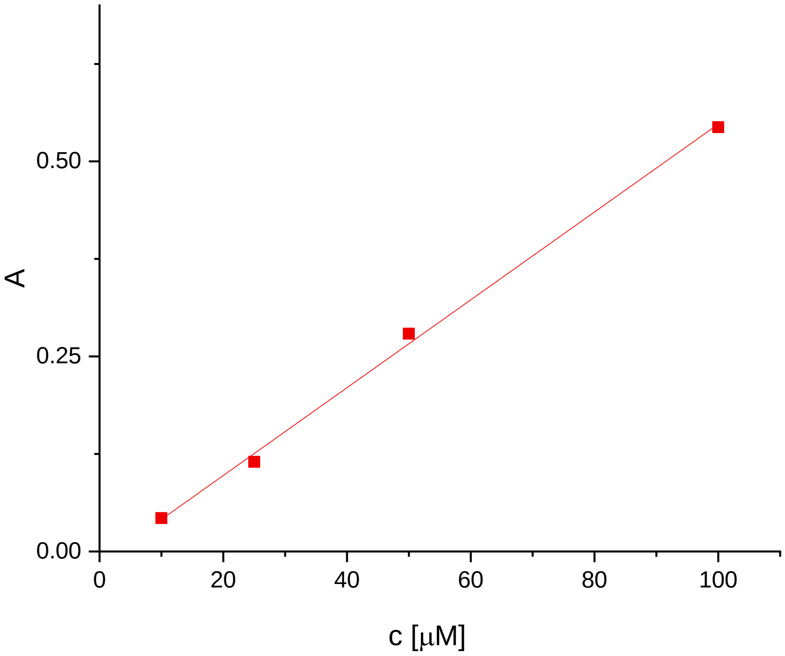

Figure S17: Concentration-dependent UV–vis absorption for determination of extinction coefficient of **8** in MeCN.

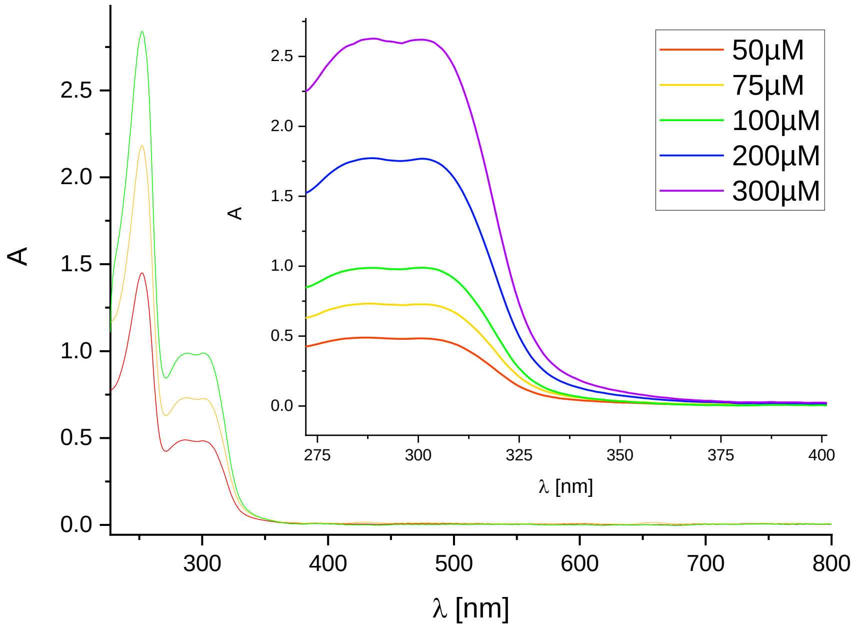

Figure S18: UV–vis absorption spectra of **9** in CH_2Cl_2.

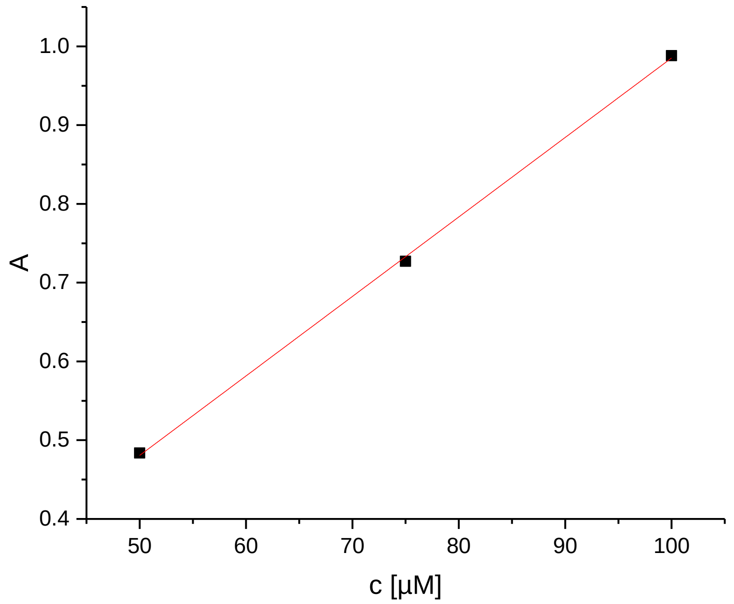

Figure S19: Concentration-dependent UV–vis absorption for determination of extinction coefficient of **9** in CH_2Cl_2.

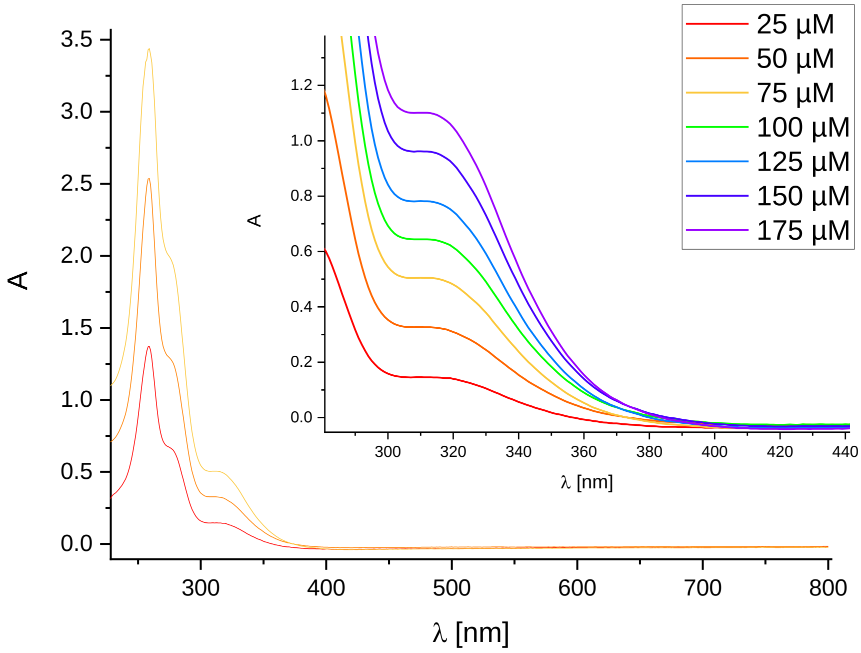

Figure S20: UV–vis absorption spectra of CH_2Cl_2 **10** in CH_2Cl_2.

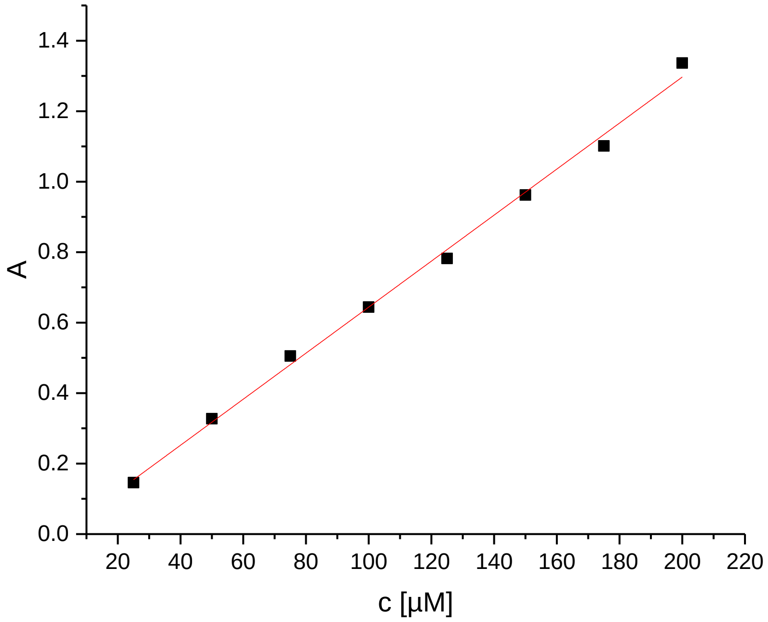

Figure S21: Concentration-dependent UV–vis absorption for determination of extinction coefficient of **10** in CH_2Cl_2.

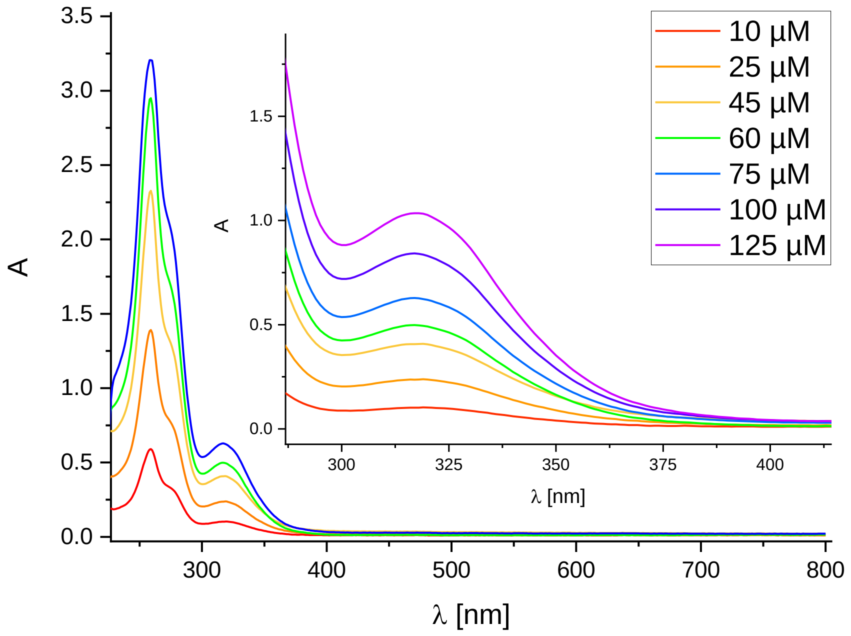

Figure S22: UV–vis absorption spectra of **11** in CH_2Cl_2.

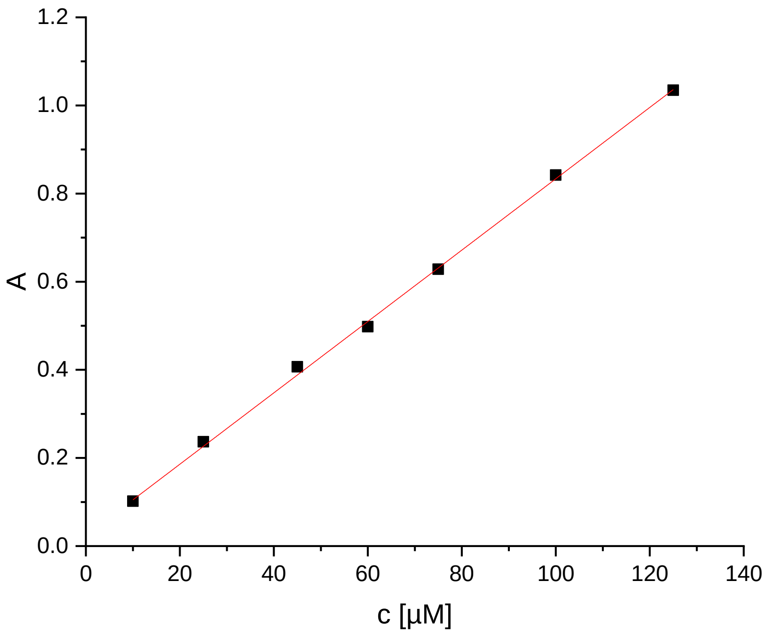

Figure S23: Concentration-dependent UV–vis absorption for determination of extinction coefficient of **11** in CH_2Cl_2.

Emission

The emission spectra were recorded at 20 °C in acetonitrile (HPLC grade, Fisher Scientific) or dichloromethane (HPLC grade, Fisher Scientific).

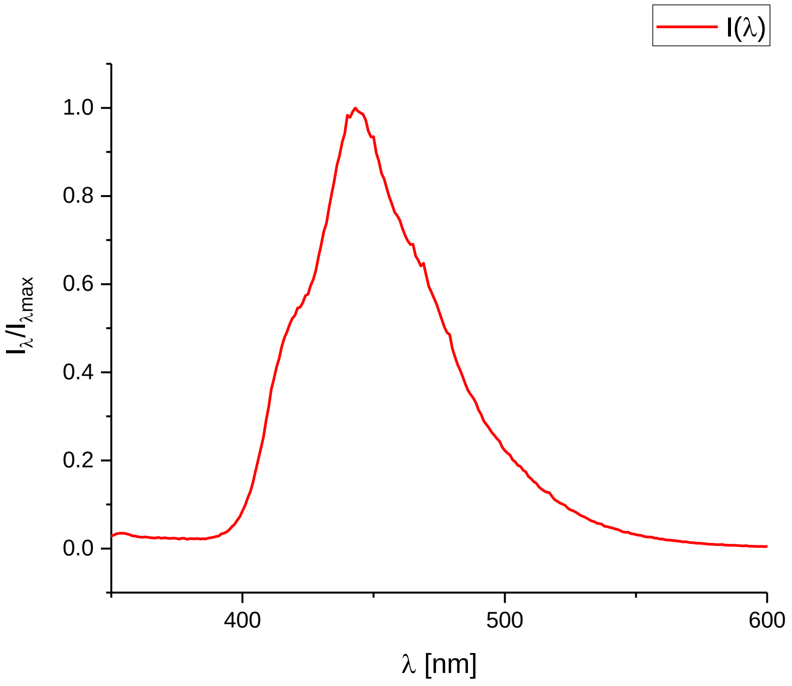

Figure S24: Normalized emission of **1** in MeCN, 100 µM, λ_{ex} = 320 nm, $slit_{ex,em}$ = 5 nm/5 nm.

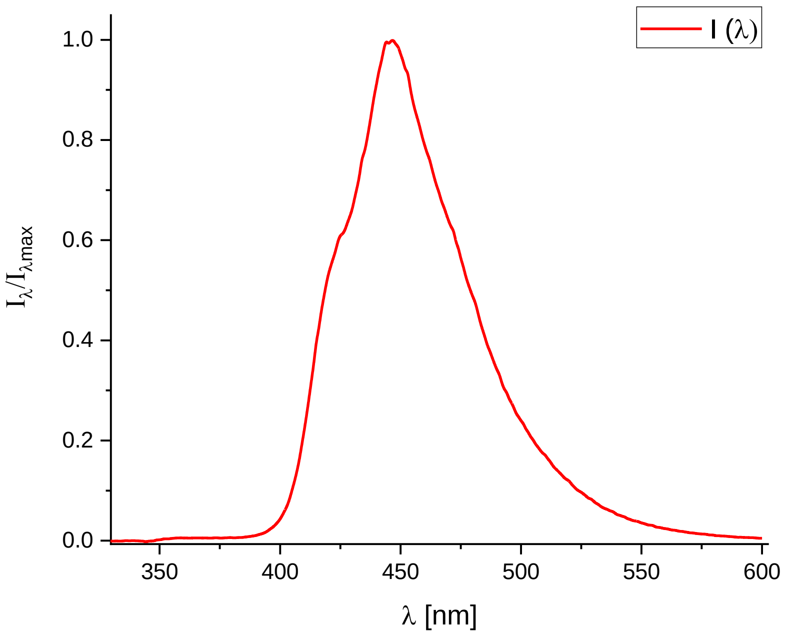

Figure S25: Normalized emission of **2** in CH_2Cl_2, 100 µM, λ_{ex} = 313 nm, slits = 5 nm/5 nm.

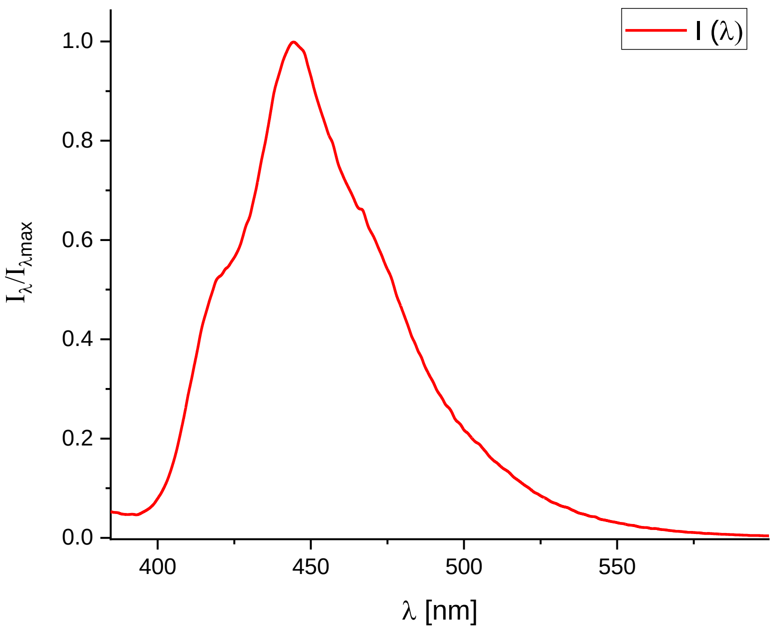

Figure S26: Normalized emission of **3** in CH_2Cl_2, 100 µM, λ_{ex} = 320 nm, slits = 5 nm/5 nm.

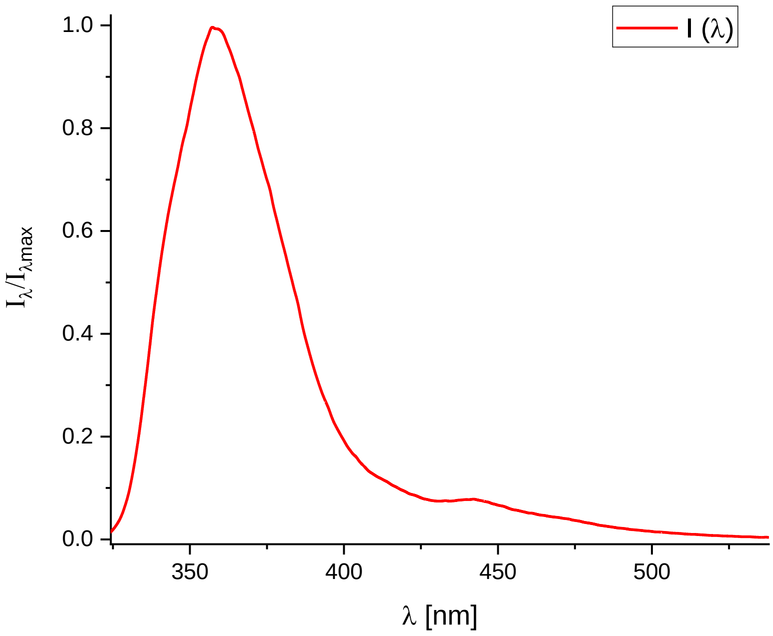

Figure S27: Normalized emission of **4** in MeCN, 100 μM, λ_{ex} = 310 nm, slits = 3 nm/3 nm.

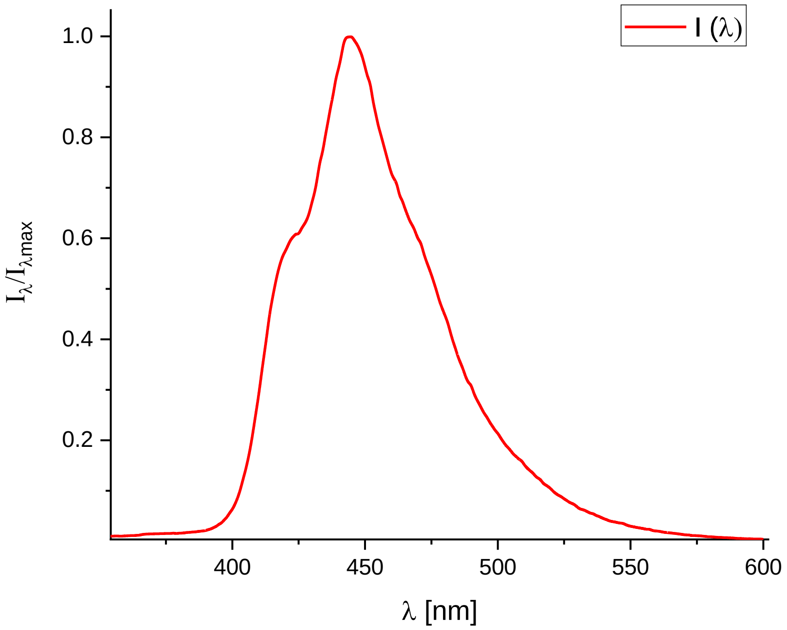

Figure S28: Normalized emission of **5** in CH_2Cl_2, 100 μM, λ_{ex} = 328 nm, slits = 5 nm/5 nm.

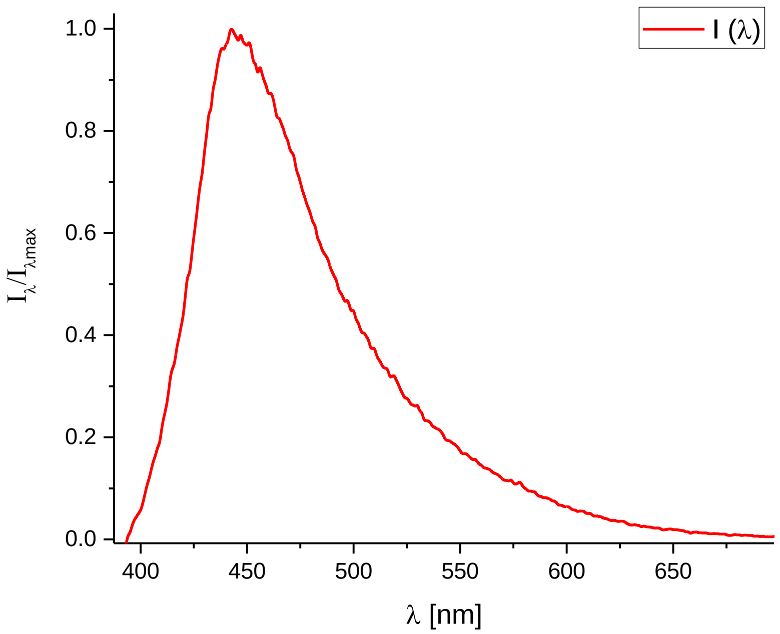

Figure S29: Normalized emission of **6** in MeCN, 50 µM, λ_{ex} = 378 nm, slits = 8 nm/8 nm.

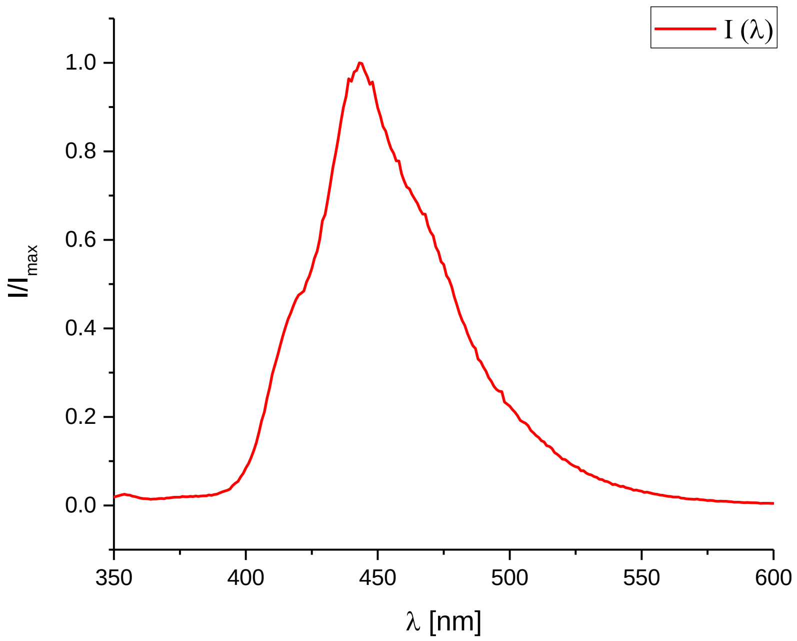

Figure S30: Normalized emission of **7** in MeCN, 100 μM, λ_{ex} = 320 nm, $slit_{ex/em}$ =5 nm/5 nm.

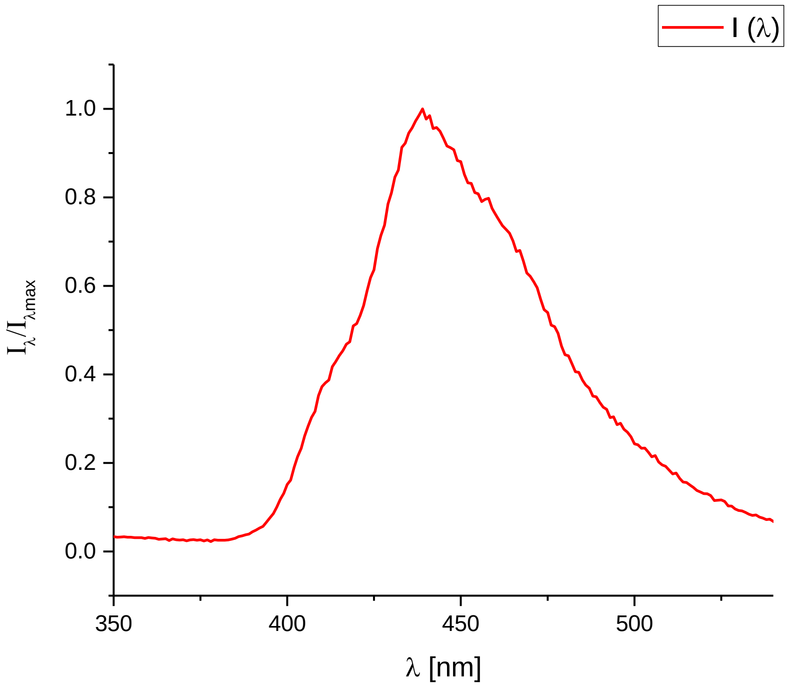

Figure S31: Normalized emission of **8** in MeCN; 100 µM, λ_{ex} = 280 nm , $slit_{ex/em}$ =5 nm/5 nm.

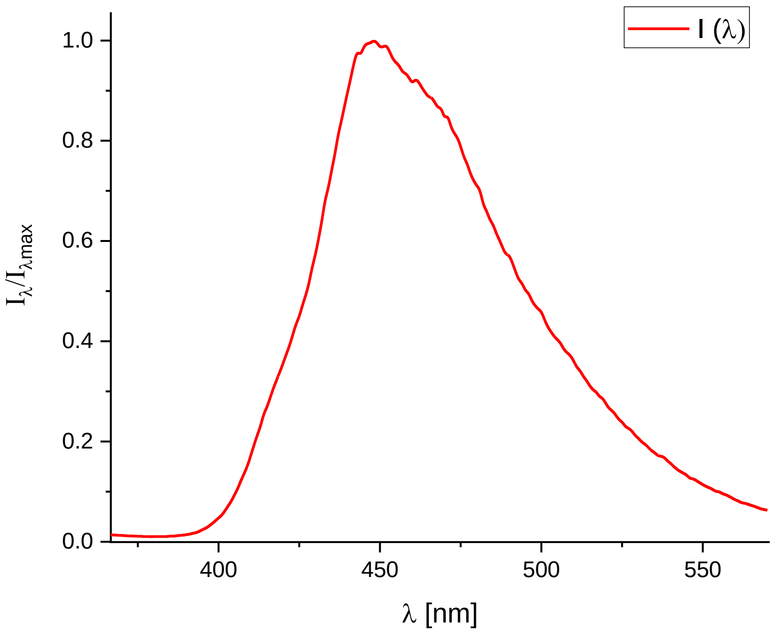

Figure S32: Normalized emission of **9** in CH_2Cl_2, 100 μM, λ_{ex} = 300 nm, slits = 5 nm/5 nm.

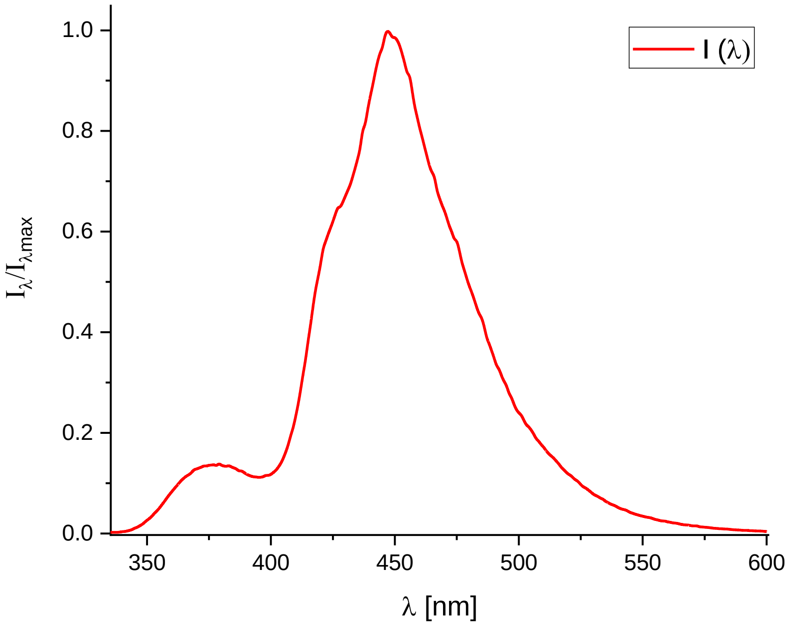

Figure S33: Normalized emission of **10** in CH_2Cl_2, 100 μM, λ_{ex} = 310 nm, slits = 5 nm/5 nm.

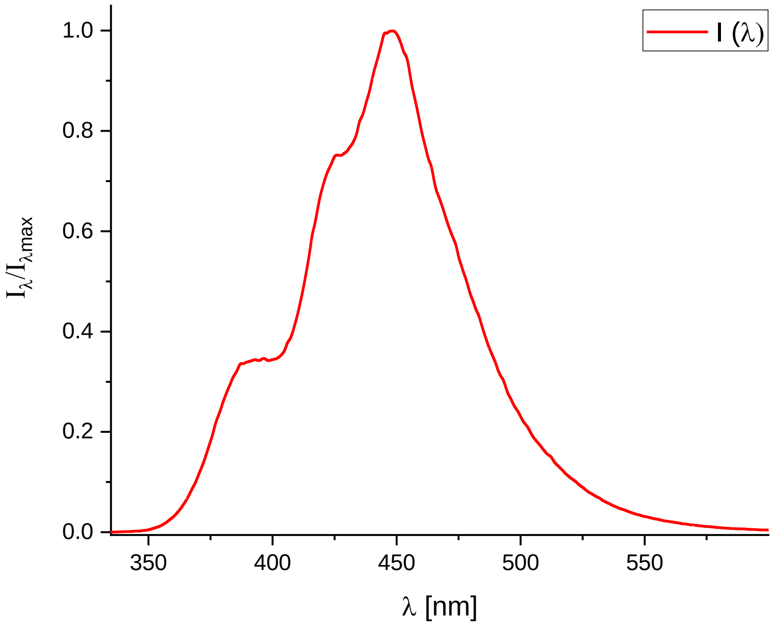

Figure S34: Normalized emission of **11** in CH_2Cl_2, 100 µM, λ_{ex} = 317 nm, slits = 6 nm/6 nm.

Cyclic voltammetry data

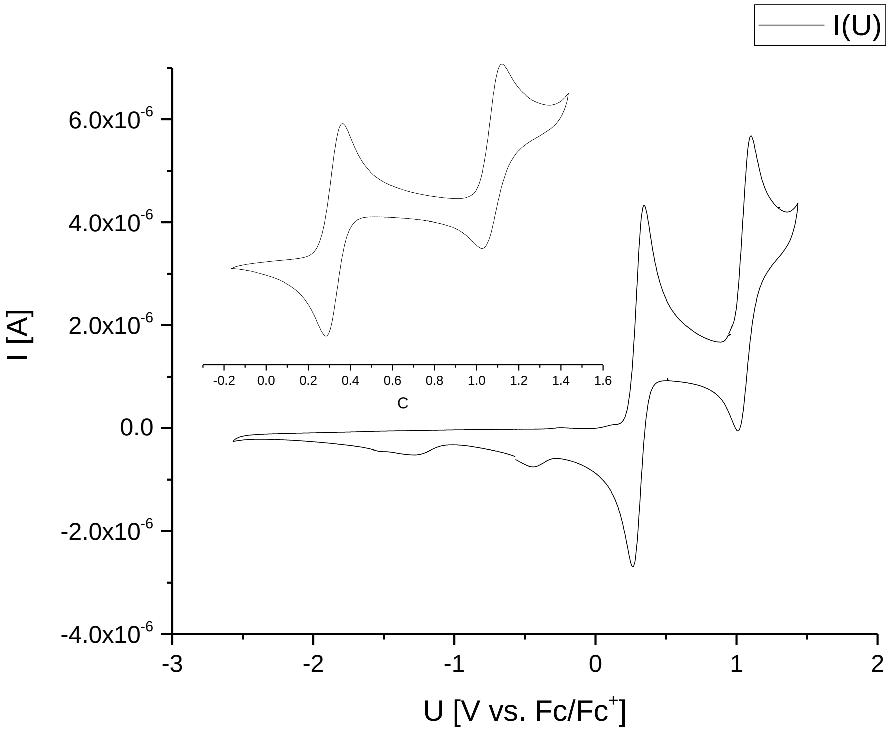

Figure S35: Cyclovoltammetry of **1** in MeCN (0.05 M NBu_4PF_6), V_s = 100 mVs^{-1}, referenced vs. Fc/Fc^+.

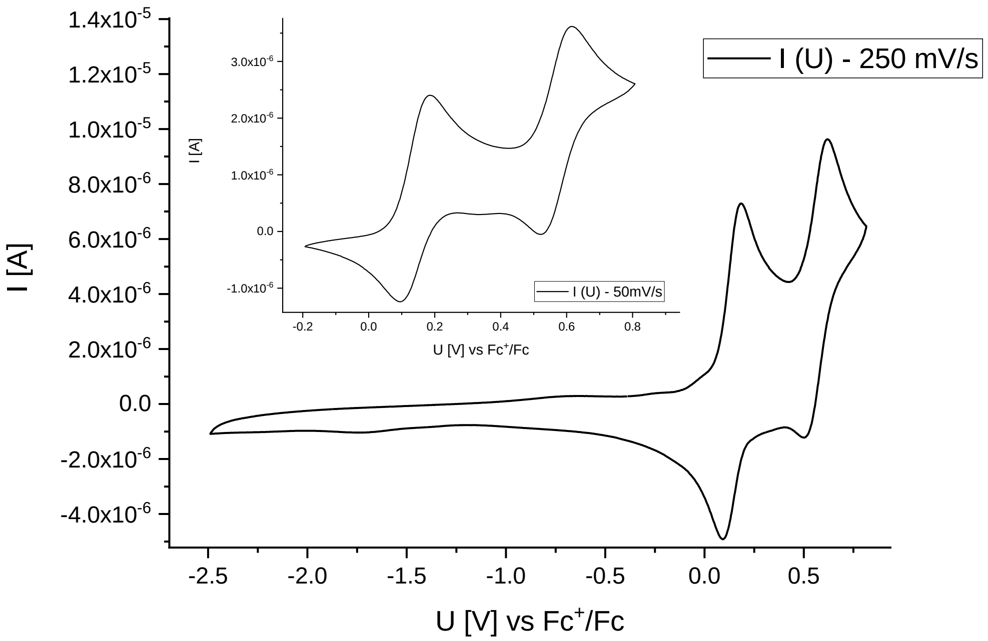

Figure S36: Cyclovoltammetry of **2** in CH_2Cl_2 (0.05 M NBu_4PF_6), V_s = 50mV/s and 250 mV/s, referenced vs. Fc/Fc^+.

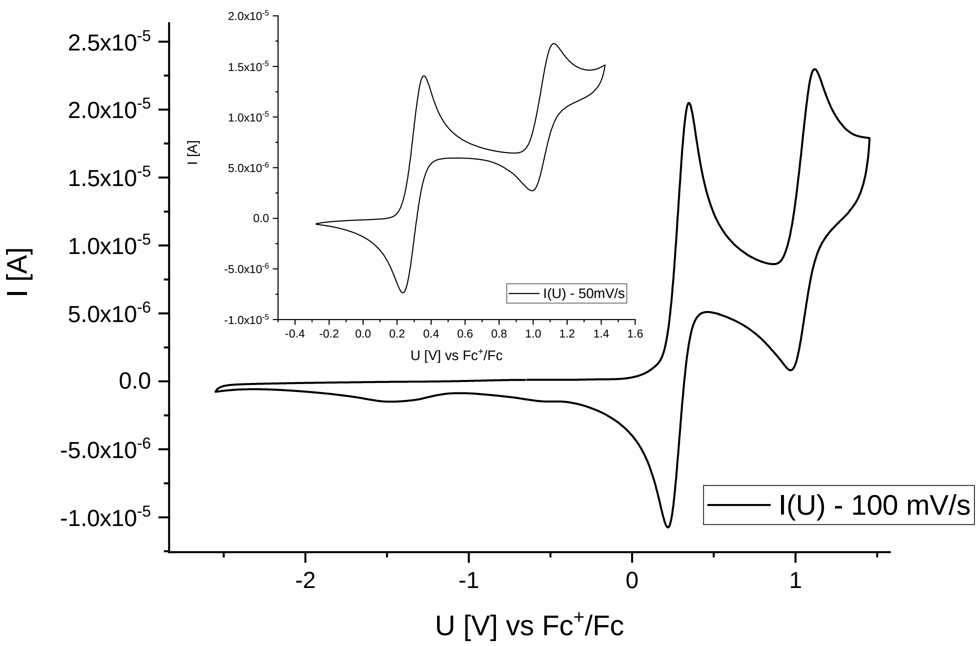

Figure S37: Cyclovoltammetry of **3** in MeCN (0.05 M NBu_4PF_6), V_s = 50 mV/s and 100 mV/s, referenced vs. Fc/Fc^+.

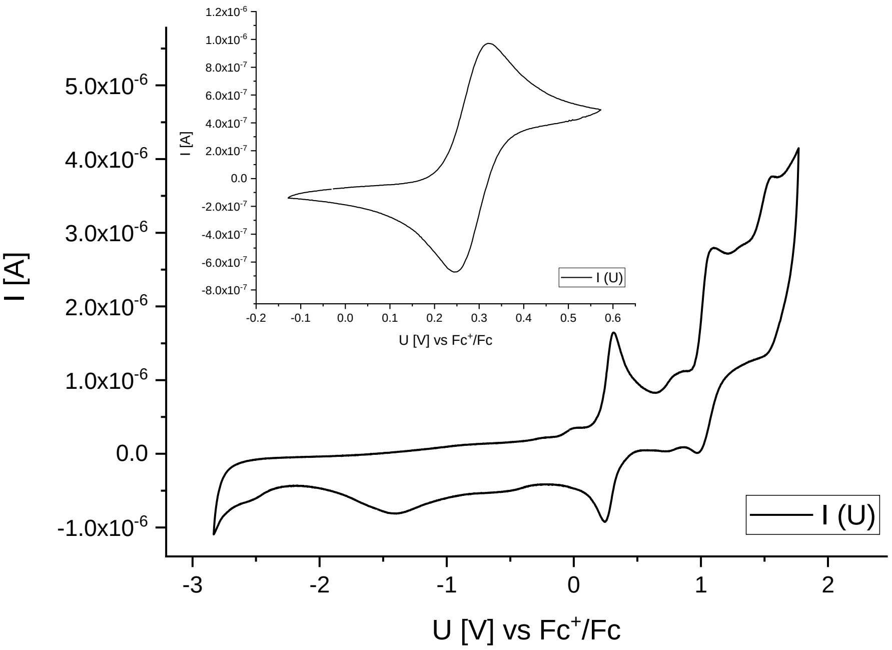

Figure S38: Cyclovoltammetry of **4** in MeCN (0.05 M NBu_4PF_6), V_s = 250 mV/s, referenced vs. Fc/Fc^+.

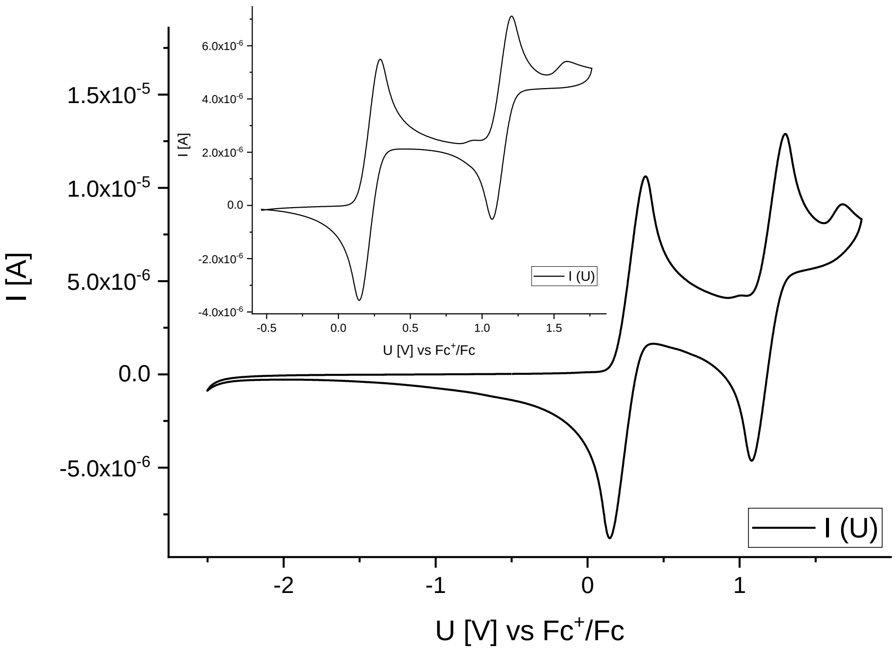

Figure S39: Cyclovoltammetry of **5** in CH_2Cl_2 (0.05 M NBu_4PF_6), V_s = 250 mV/s, referenced vs. Fc/Fc^+.

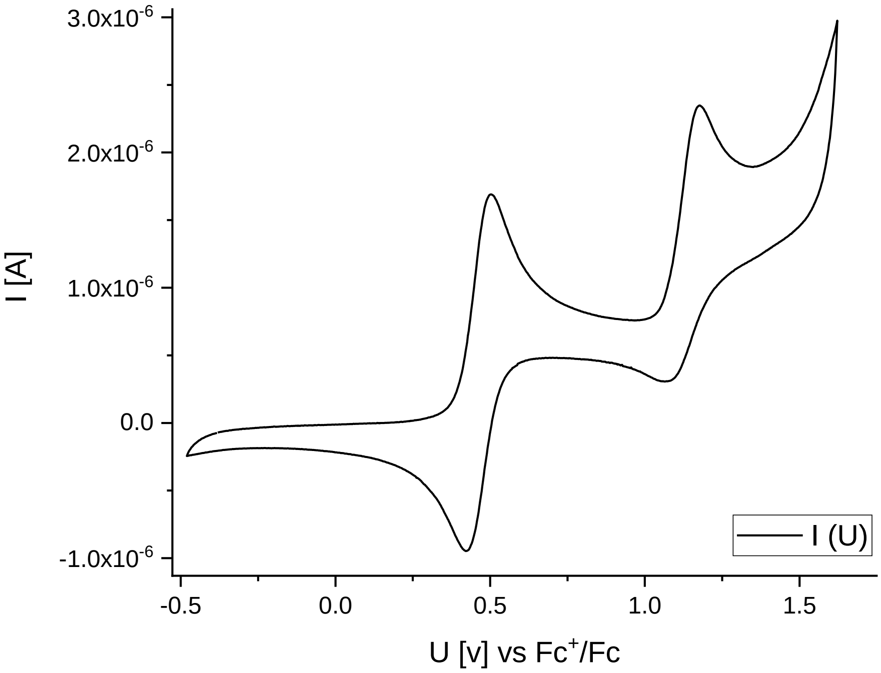

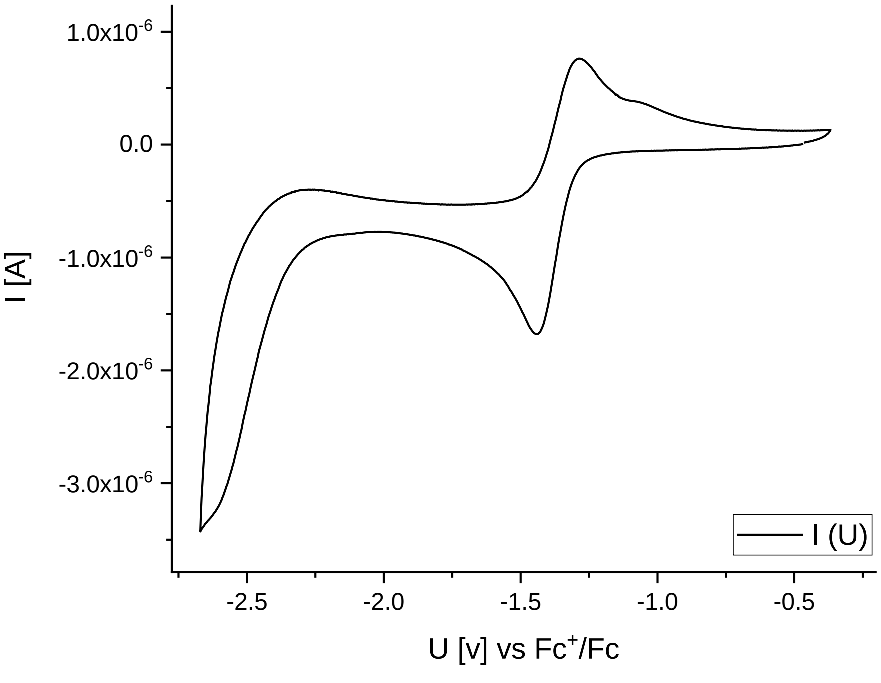

Figure S40: Cyclovoltammetry of **6** in MeCN (0.05 M NBu_4PF_6), V_s = 100 mV/s, referenced vs. Fc/Fc^+.

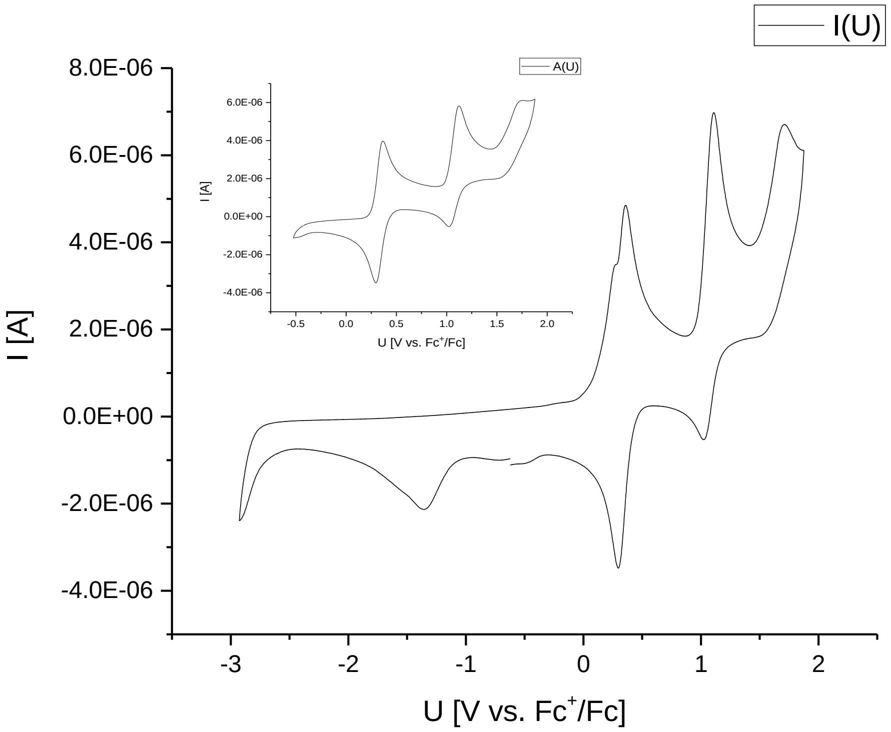

Figure S41: Cyclovoltammetry of **7** in MeCN (0.05 M NBu_4PF_6), V_s = 250 mV/s, referenced vs. Fc/Fc^+. Reduction at −1.3V belongs to irreversible reduction induced by irreversible oxidation second potential.

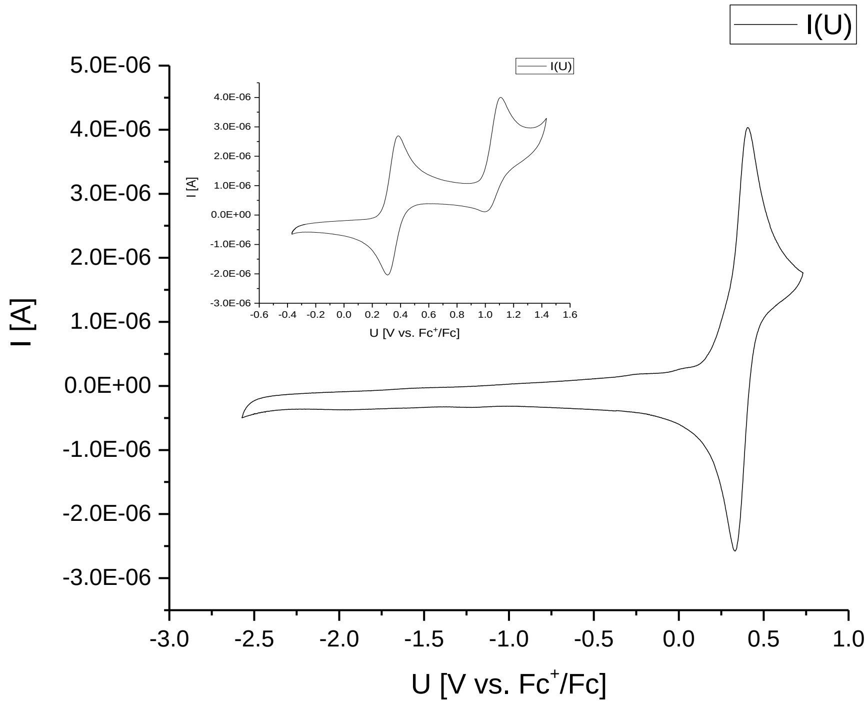

Figure S42: Cyclovoltammetry of **8** in MeCN (0.05 M NBu_4PF_6), V_s = 250 mV/s, referenced vs. Fc/Fc^+. Irreversible reduction bands are coming up if scanning higher than first oxidation.

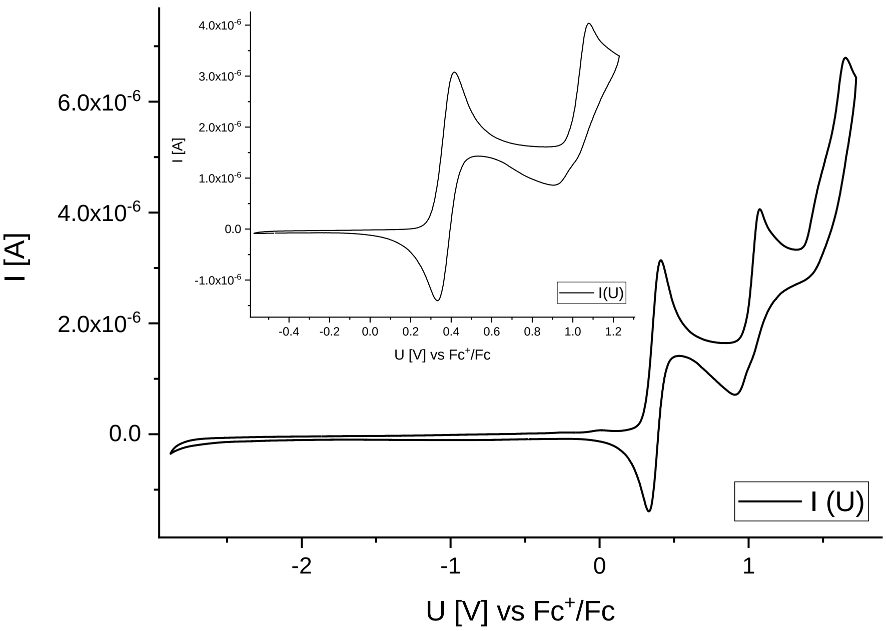

Figure S43: Cyclovoltammetry of **9** in MeCN (0.05 M NBu_4PF_6), V_s = 50 mV/s, referenced vs. Fc/Fc^+.

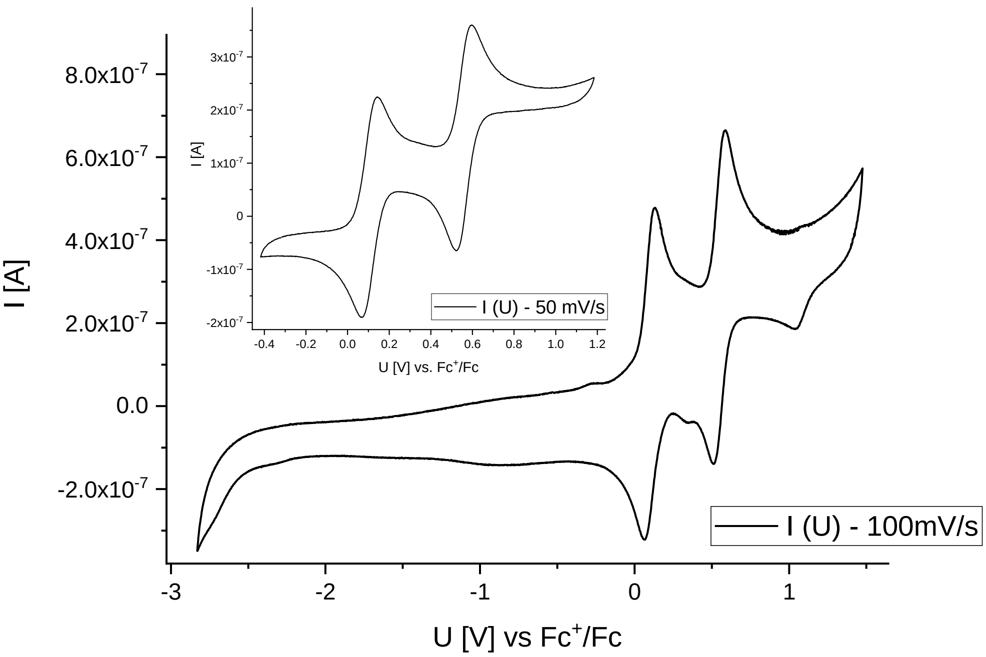

Figure S44: Cyclovoltammetry of **10** in MeCN (0.05 M NBu_4PF_6), V_s = 50 mV/s and 100 mV/s., referenced vs. Fc/Fc^+.

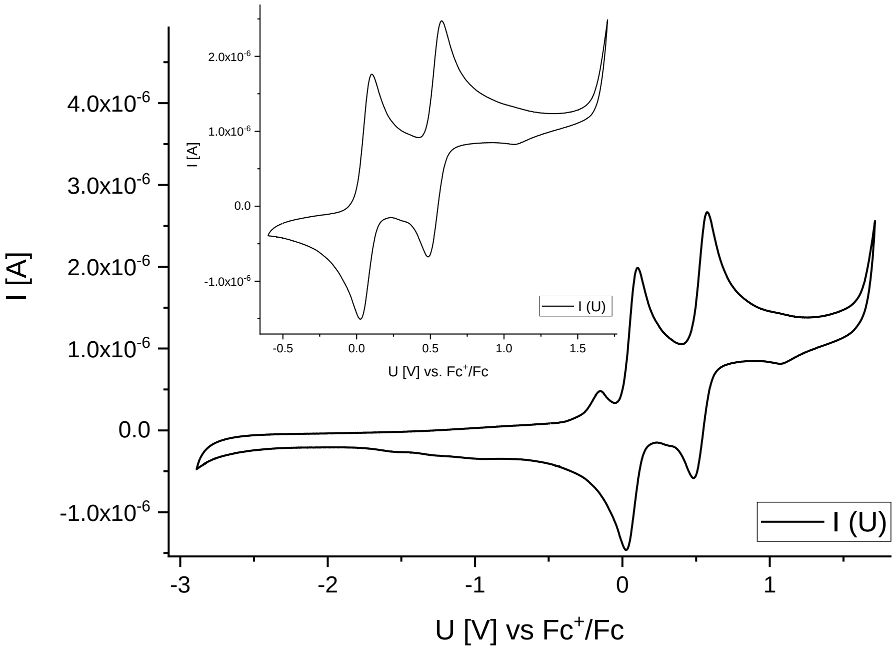

Figure S45: Cyclovoltammetry of **11** in MeCN (0.05 M NBu_4PF_6), V_s = 100 mV/s, referenced vs. Fc/Fc^+.

NMR spectroscopic data

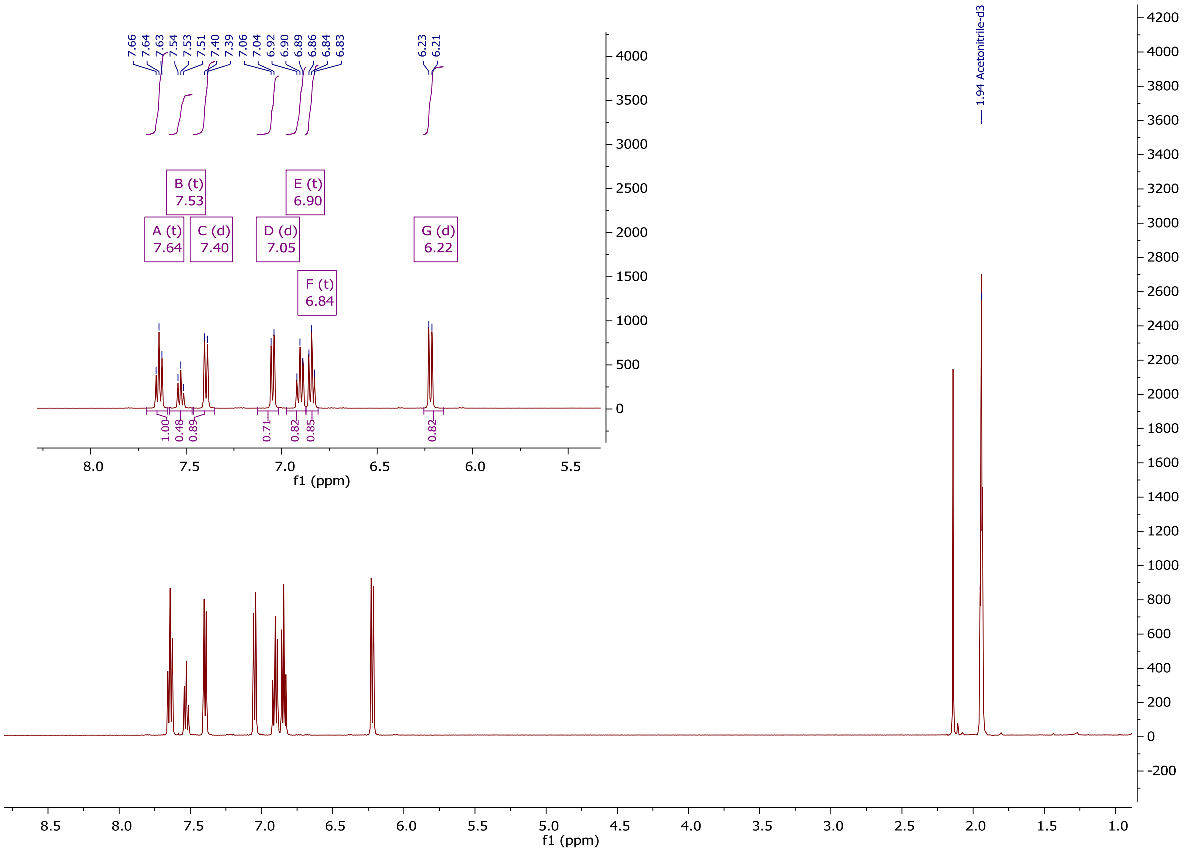

Figure S46: ^{1}H NMR spectrum of **1**.

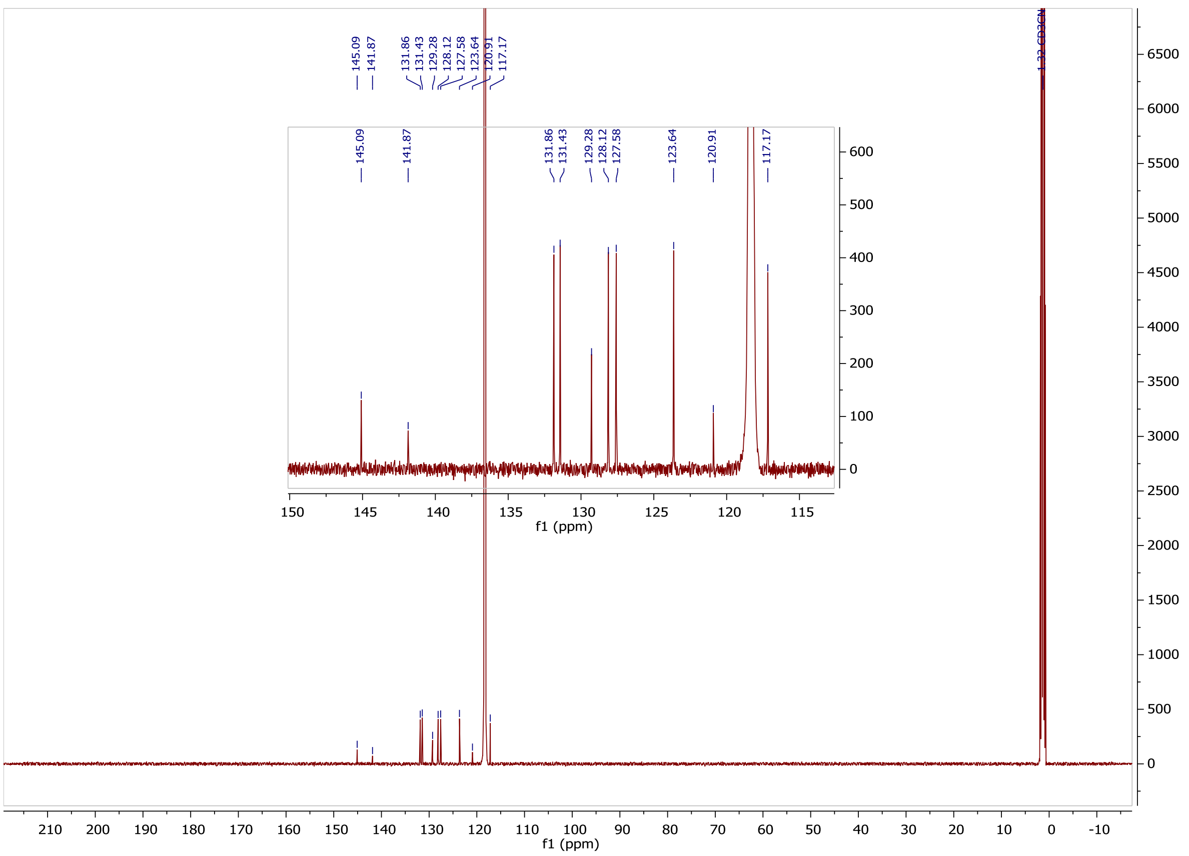

Figure S47: ^{13}C NMR spectrum of **1**.

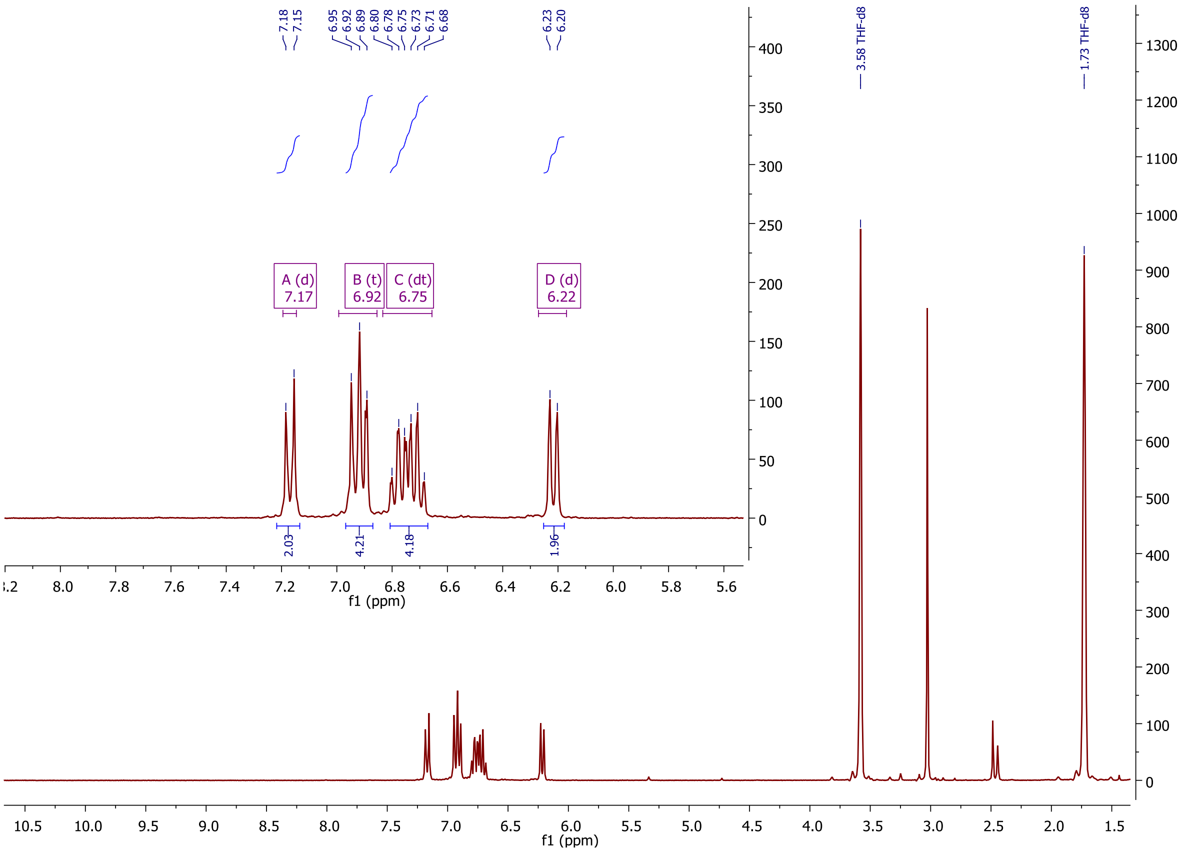

Figure S48: ^{1}H NMR spectrum of **2**.

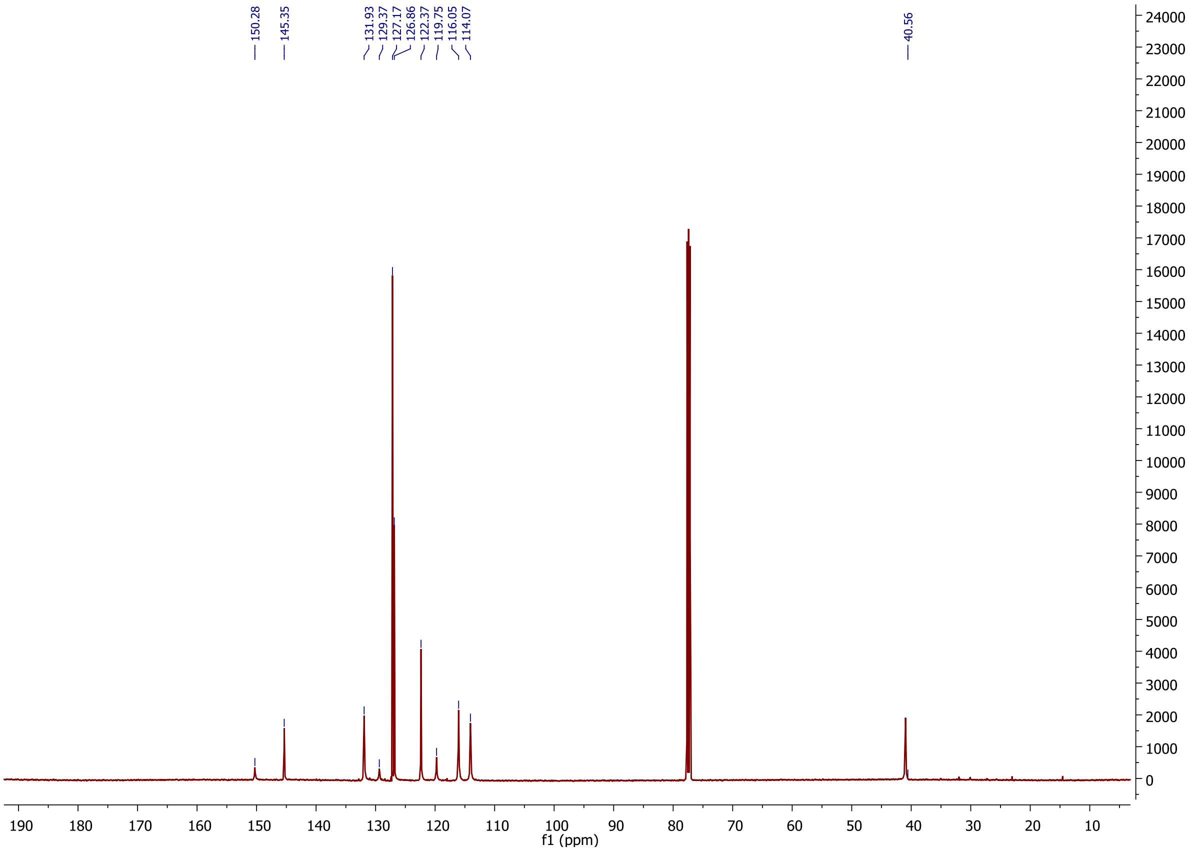

Figure S49: ^{13}C NMR spectrum of **2**.

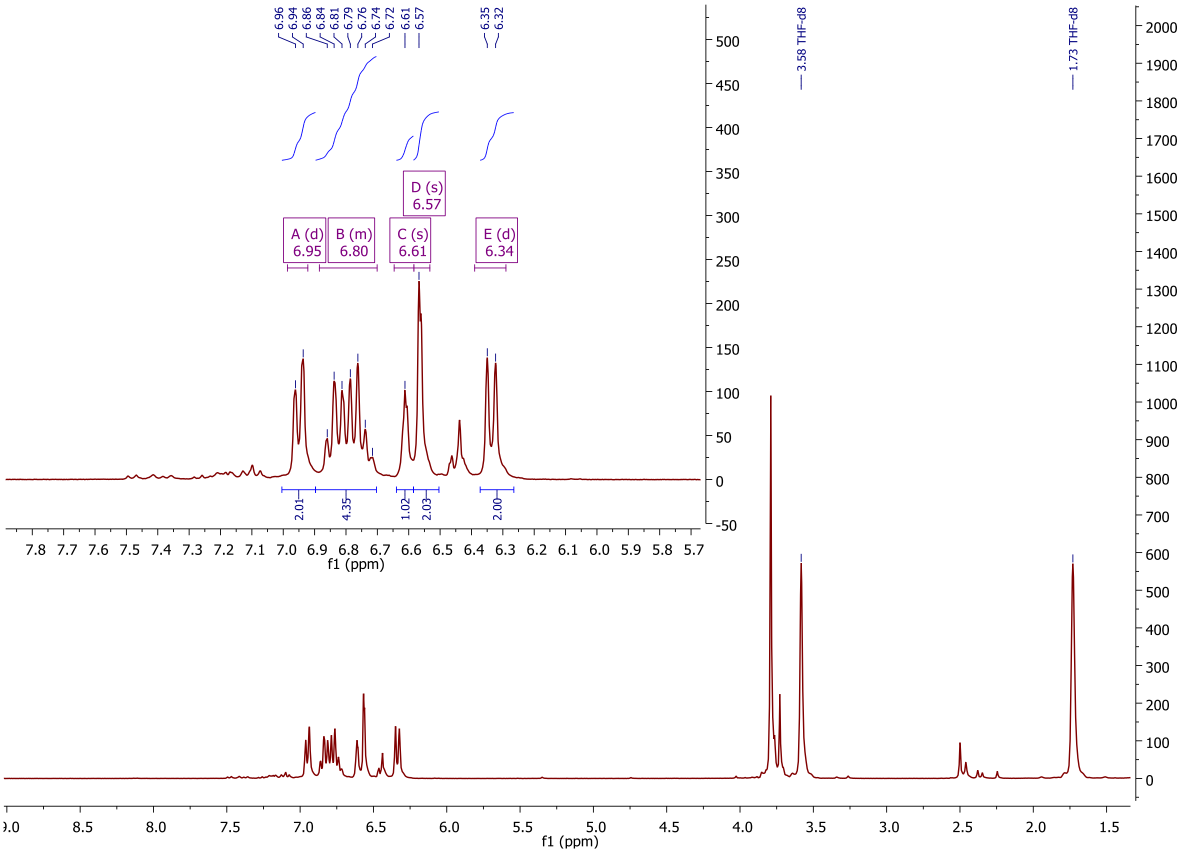

Figure S50: ^{1}H NMR spectrum of **3**.

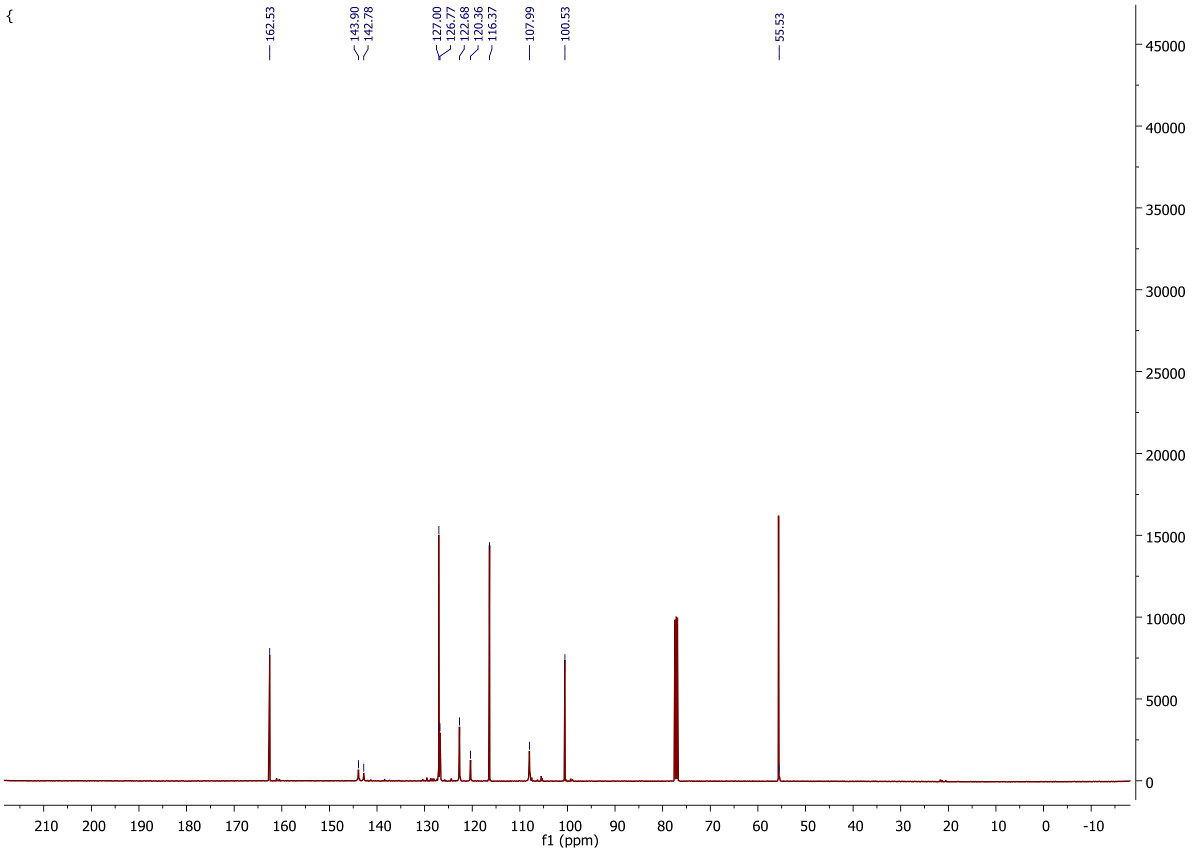

Figure S51: ^{13}C NMR spectrum of **3**.

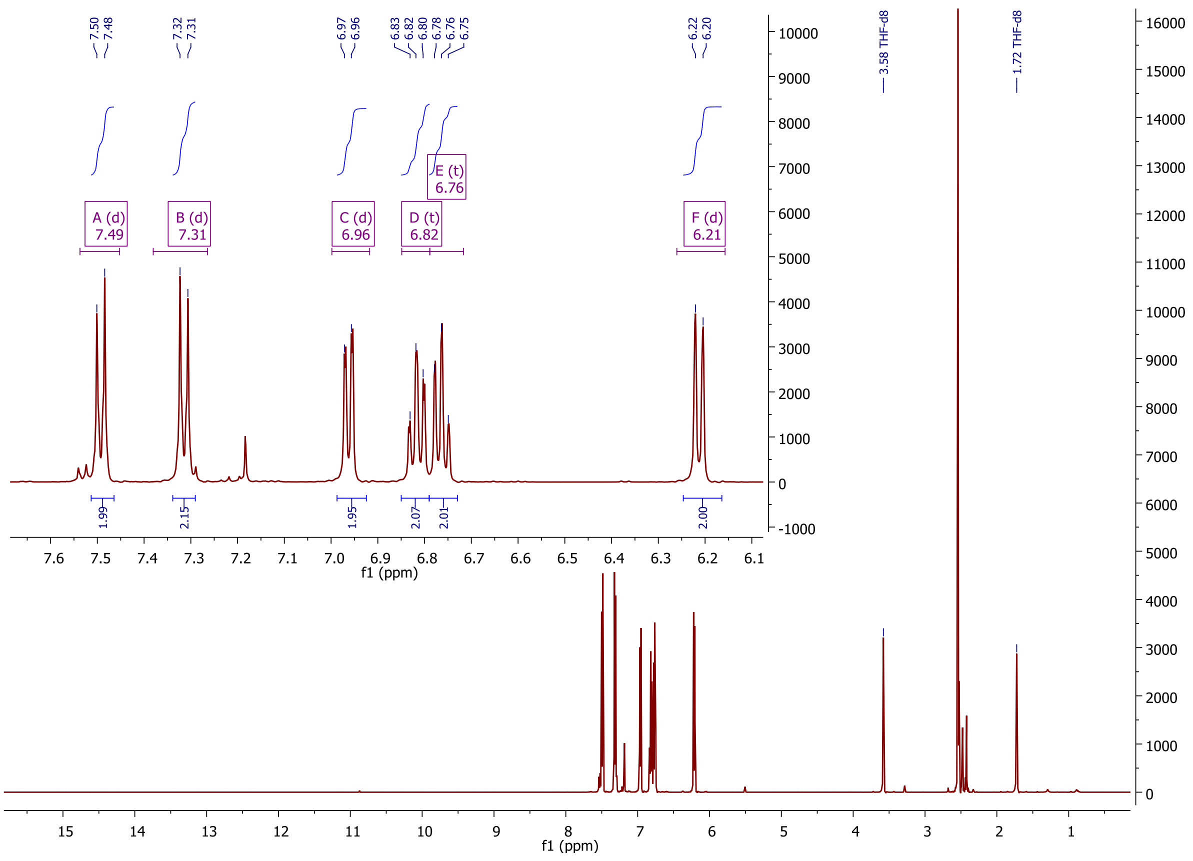

Figure S52: ^{1}H NMR spectrum of **4**.

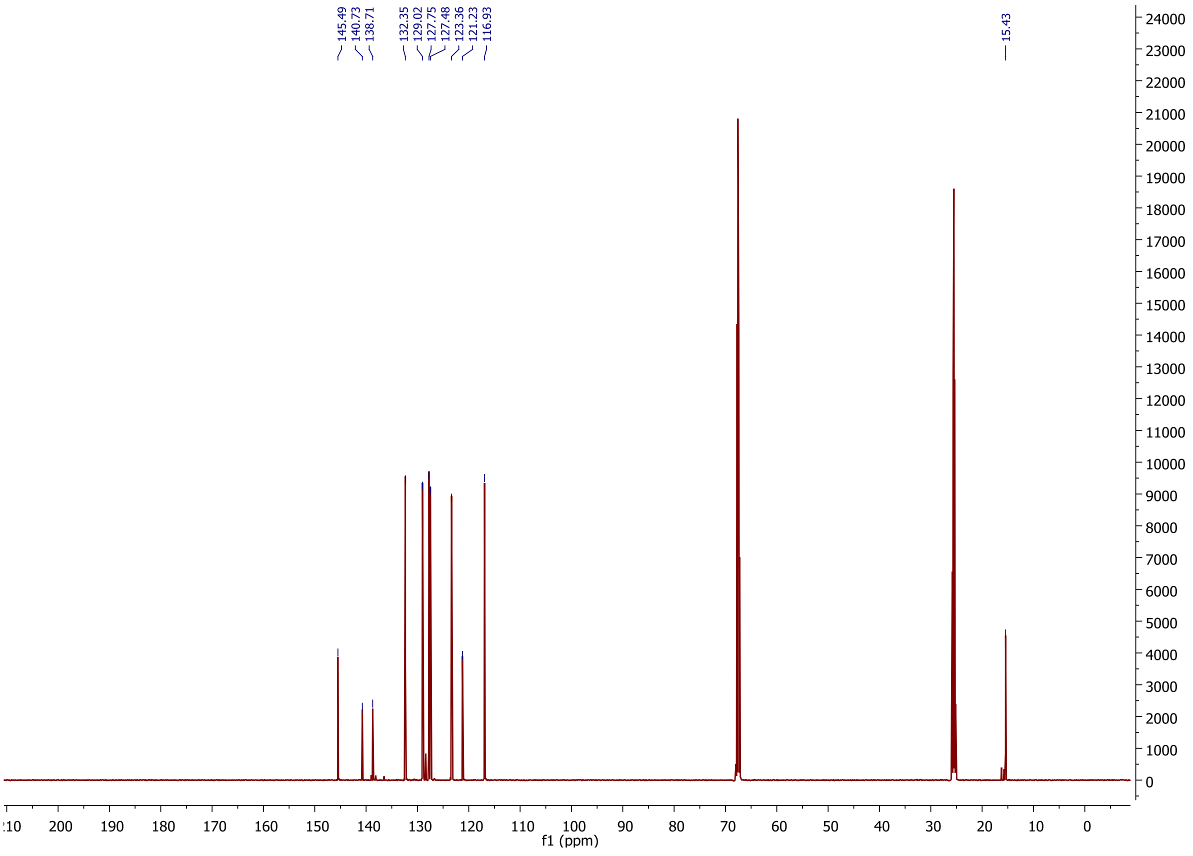

Figure S53: ^{13}C NMR spectrum of **4**.

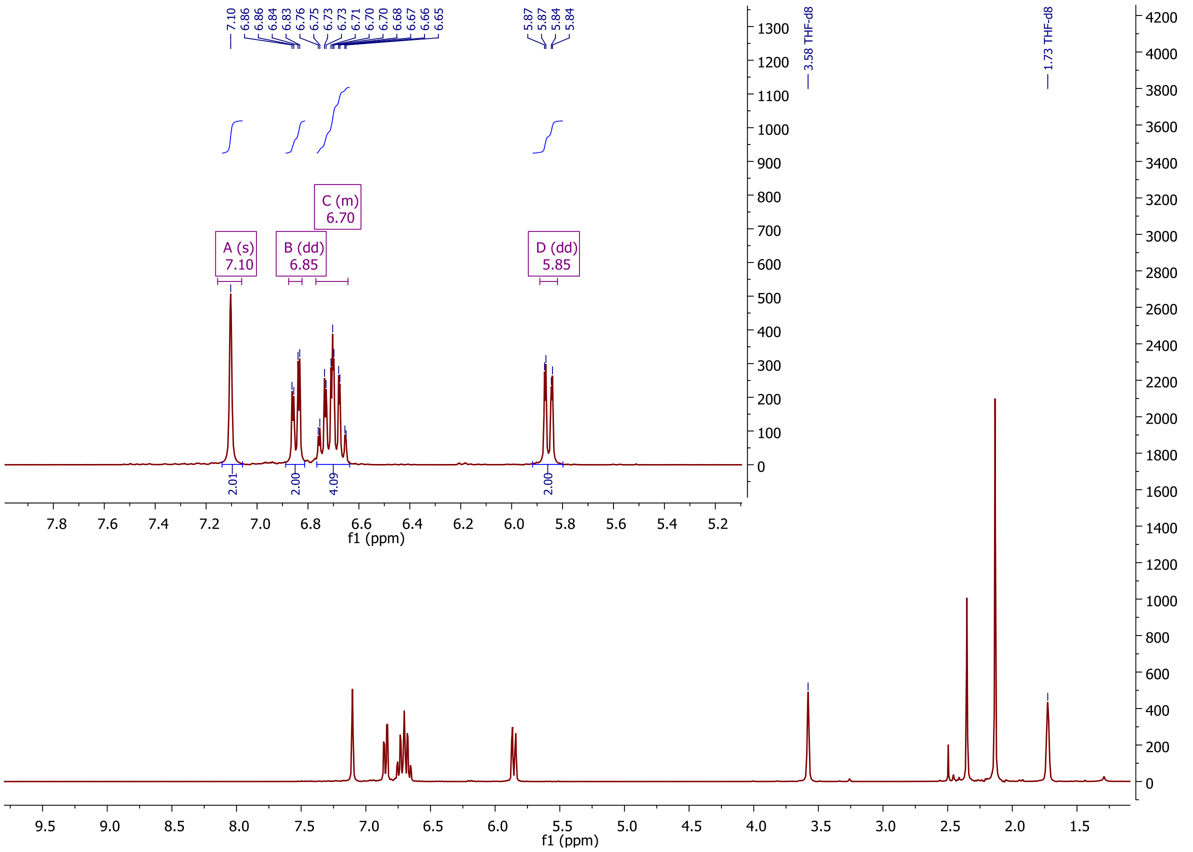

Figure S54: ^{1}H NMR spectrum of **5**.

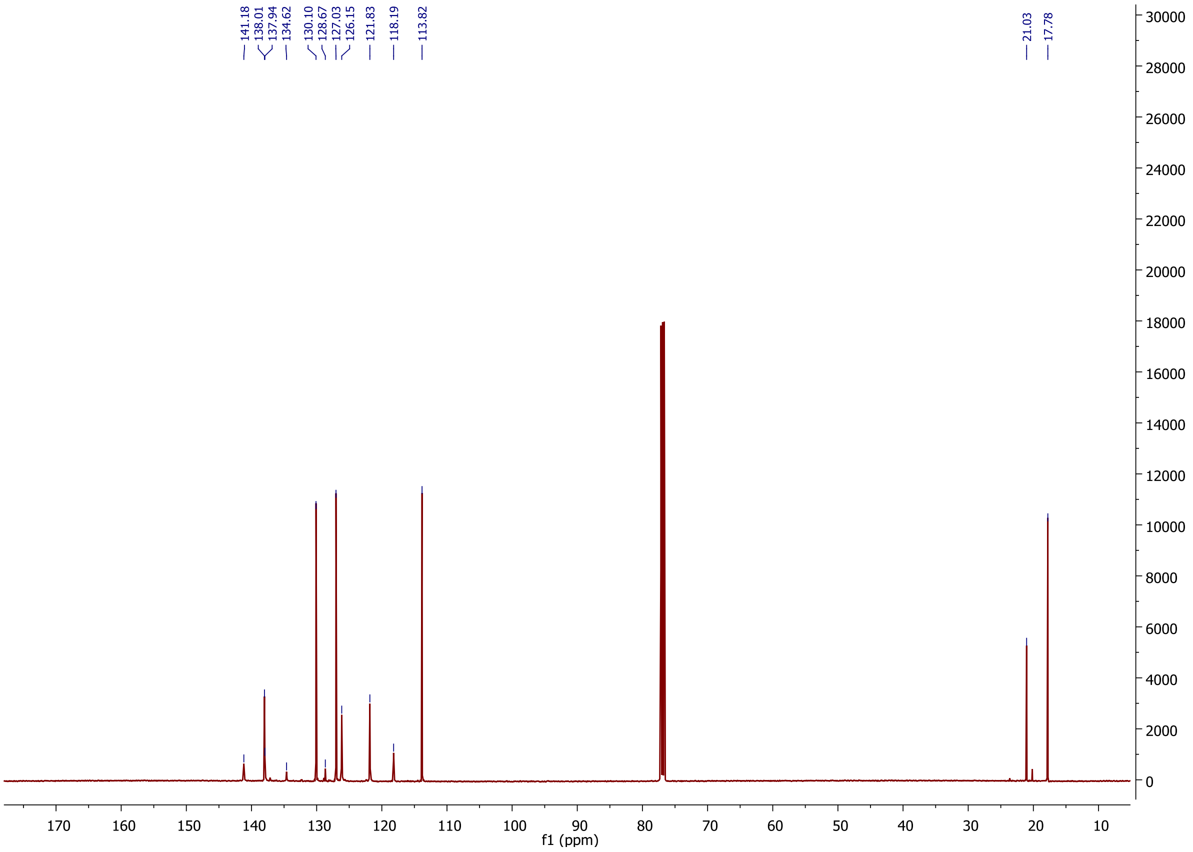

Figure S55: ^{13}C NMR spectrum of **5**.

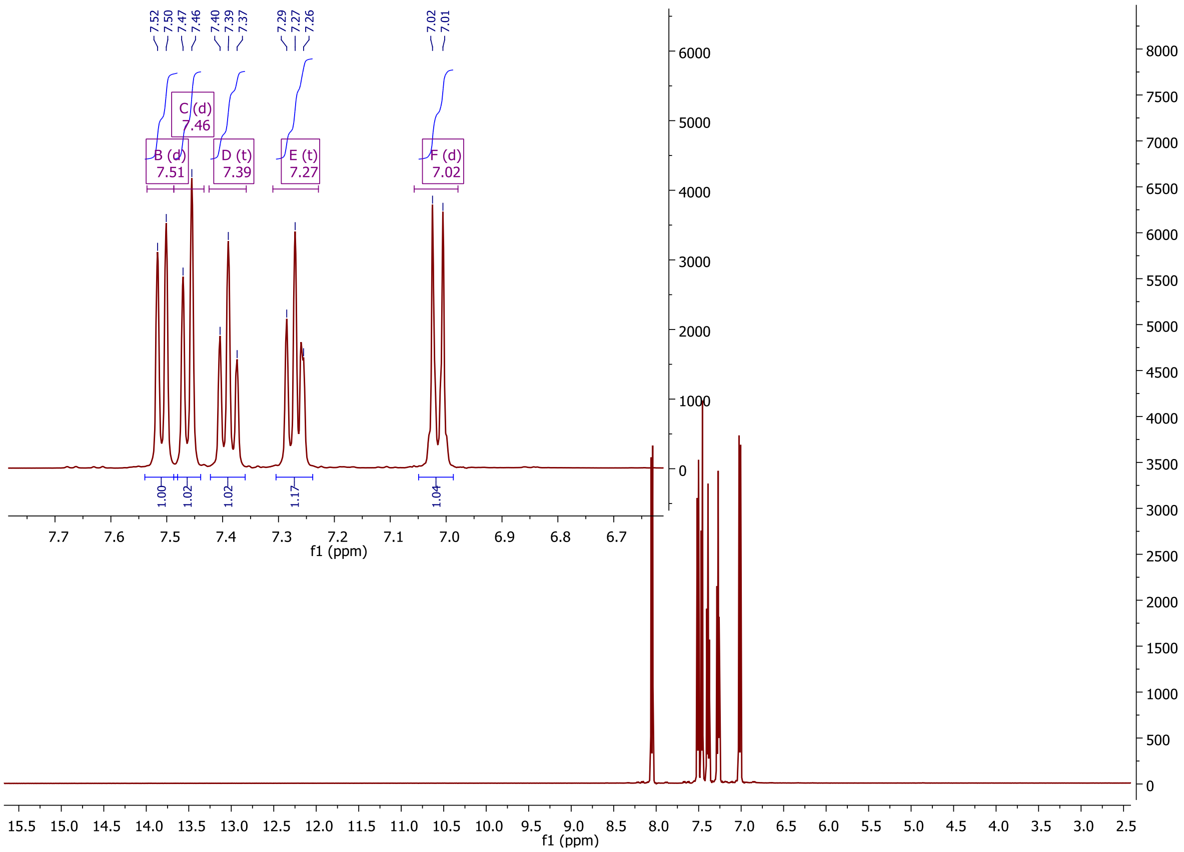

Figure S56: ^{1}H NMR spectrum of **6**.

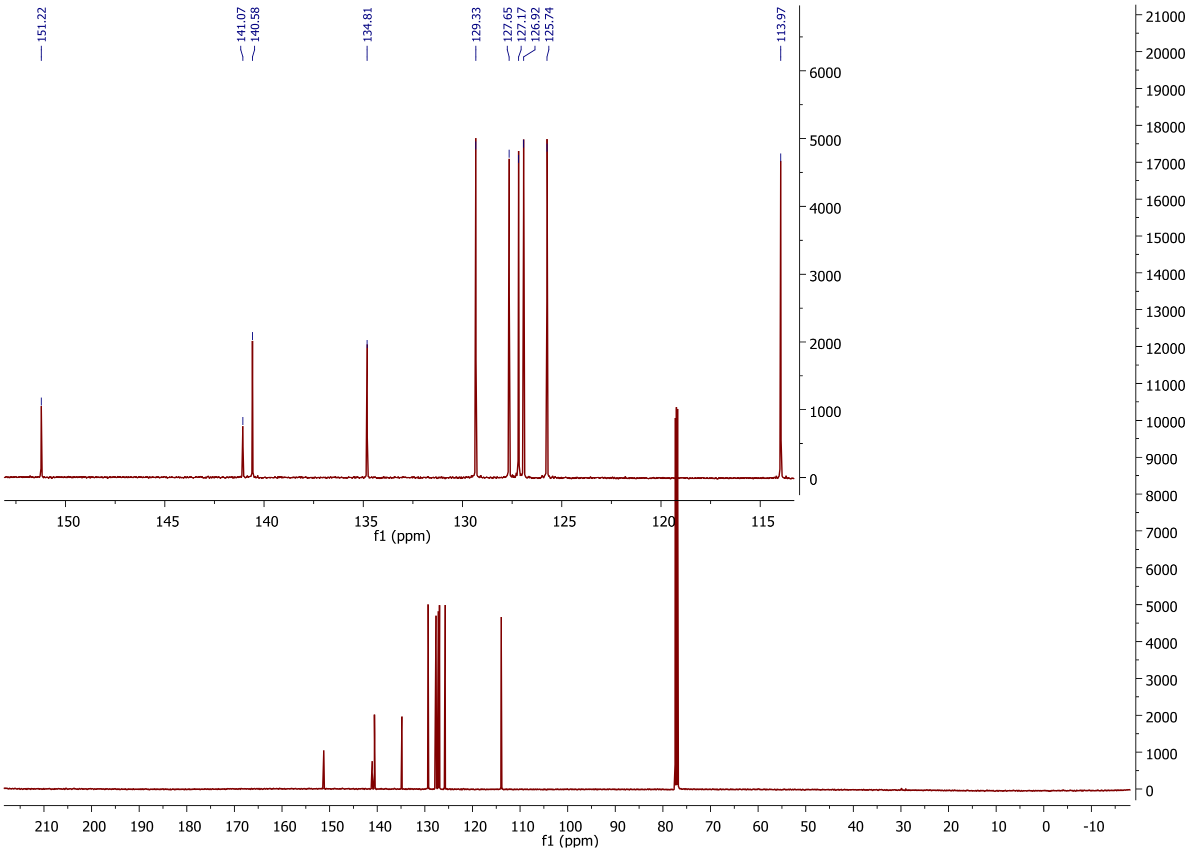

Figure S57: ^{13}C NMR spectrum of **6**.

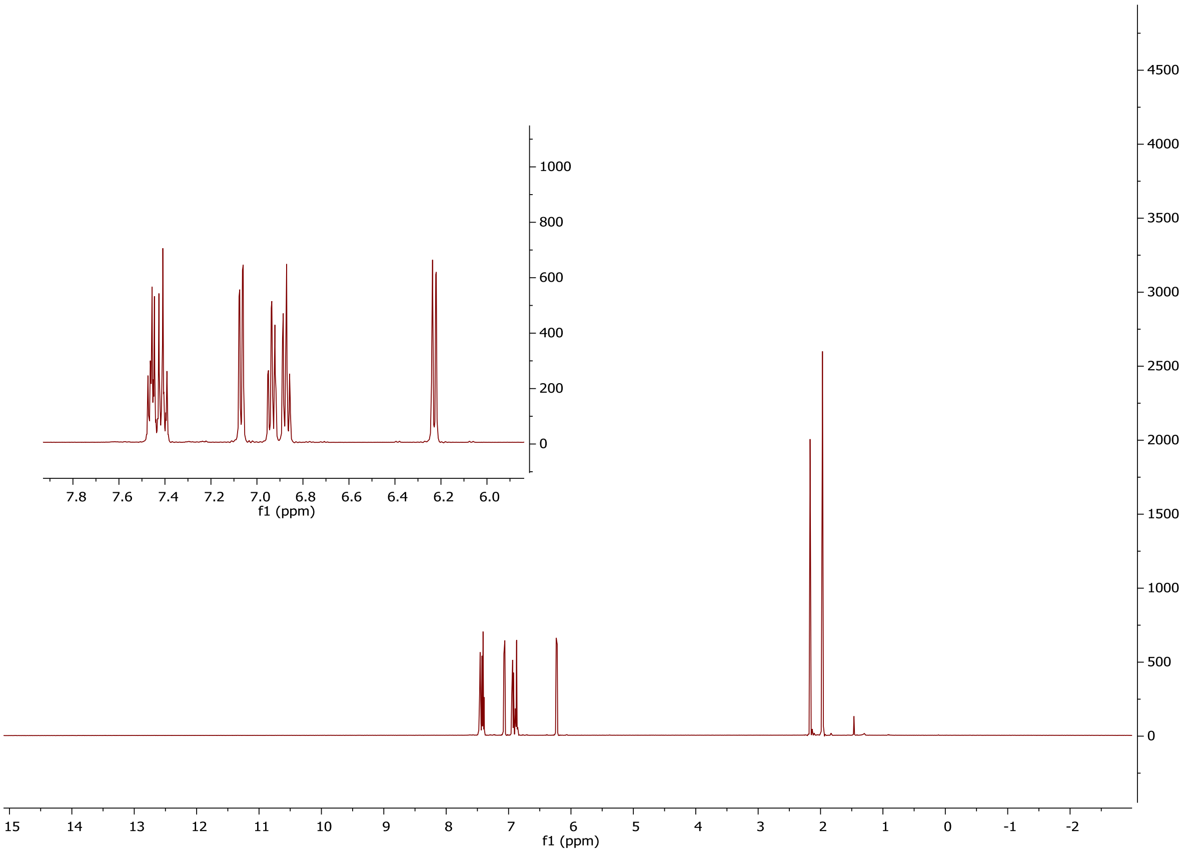

Figure S58: ^{1}H NMR spectrum of **7**.

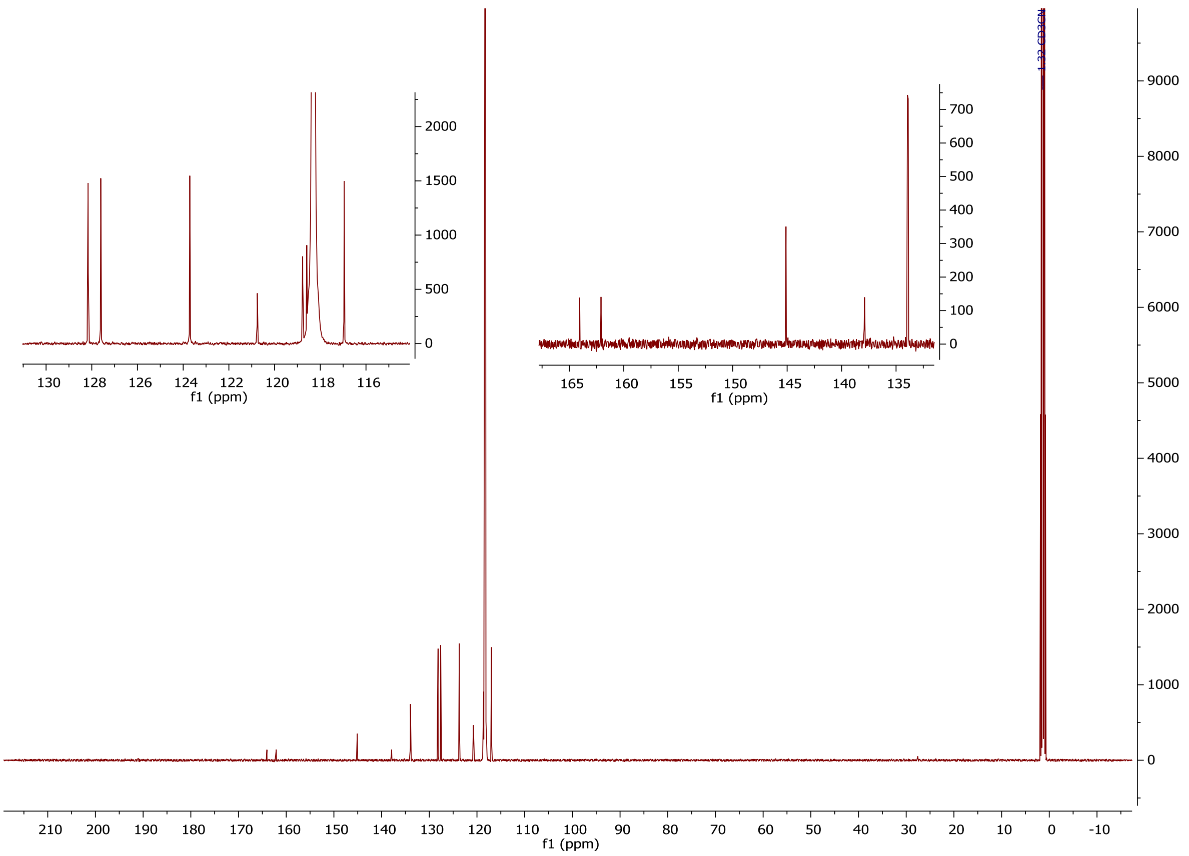

Figure S59: ^{13}C NMR spectrum of **7**.

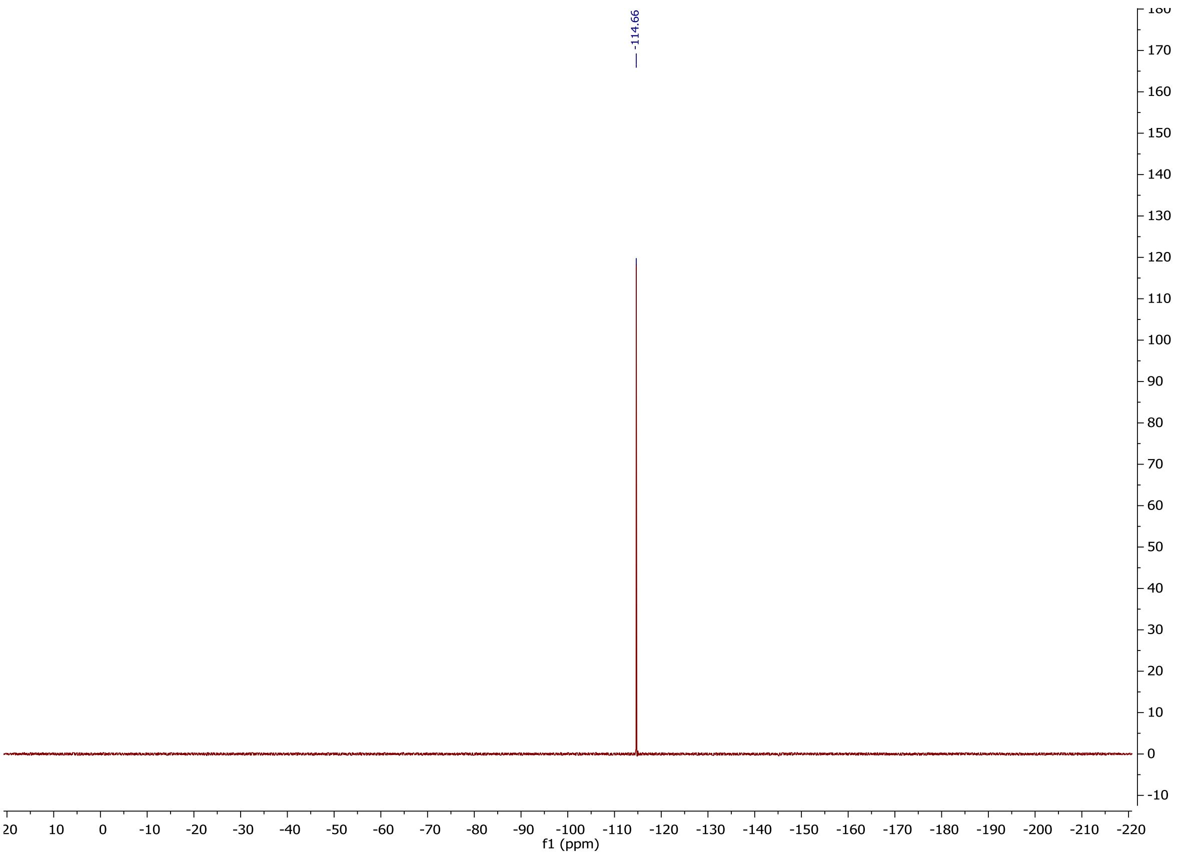

Figure S60: ^{19}F NMR spectrum of **7**.

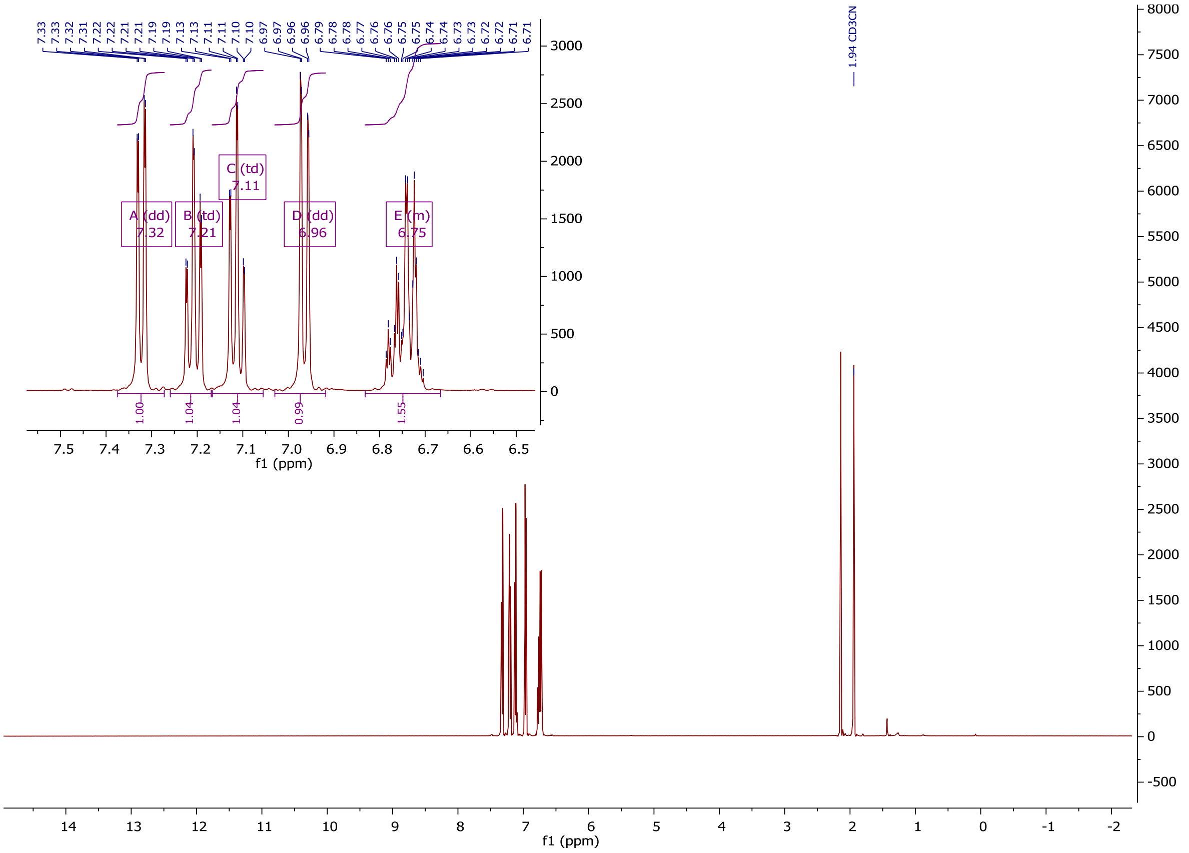

Figure S61: ^{1}H NMR spectrum of **8**.

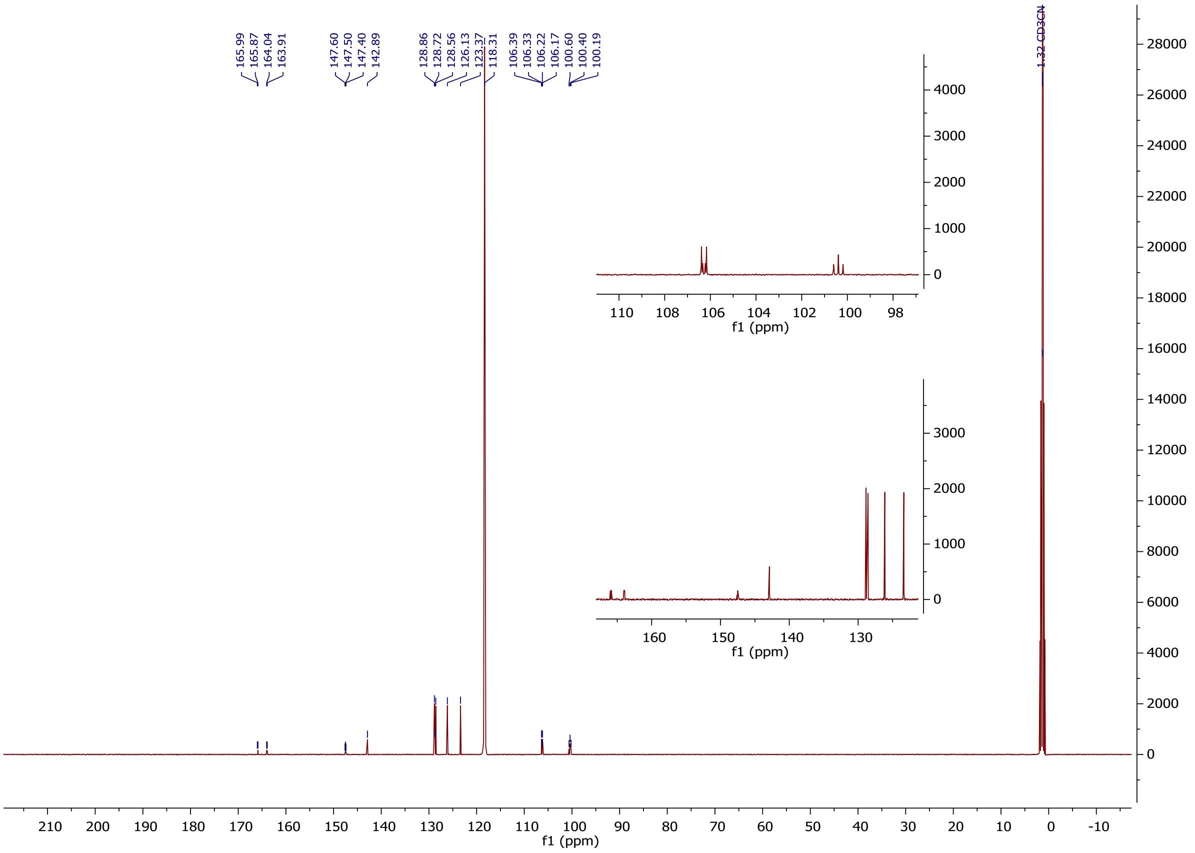

Figure S62: ^{13}C NMR spectrum of **8**.

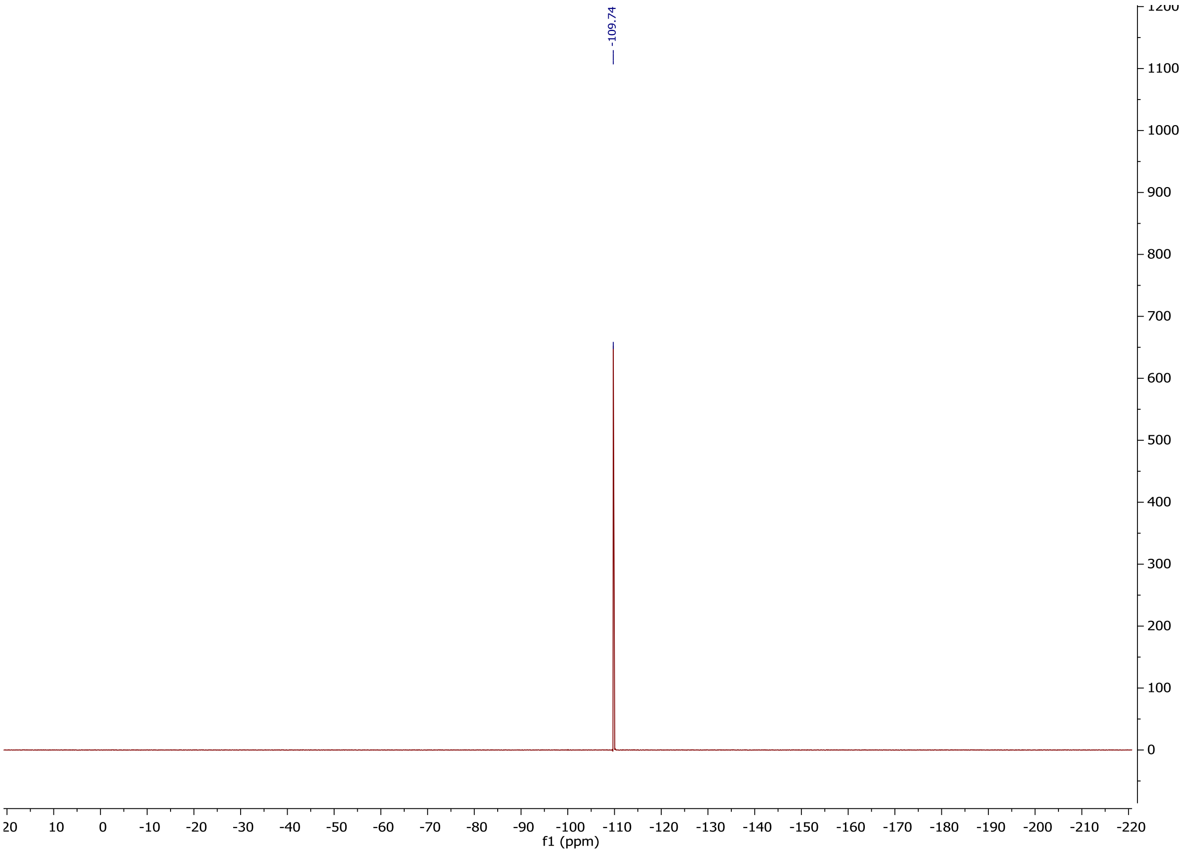

Figure S63: ^{19}F NMR spectrum of **8**.

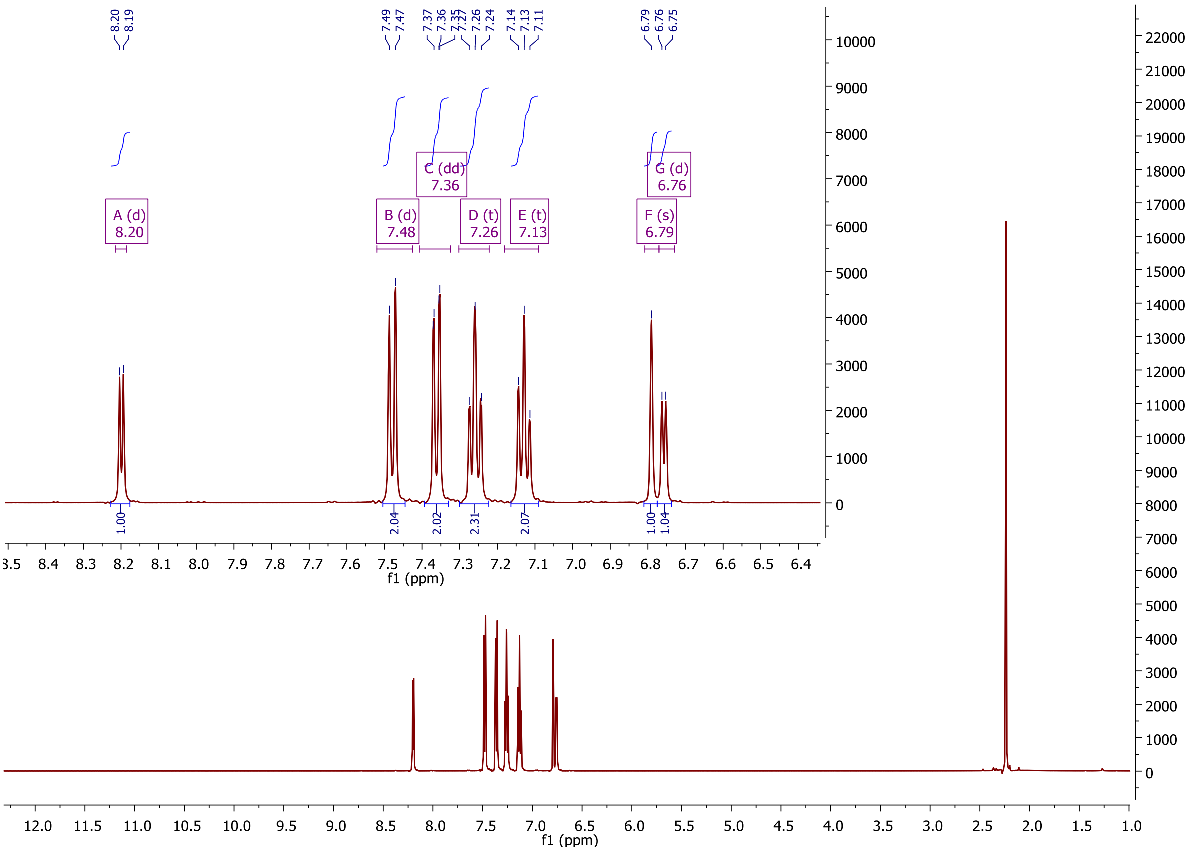

Figure S64: ^{1}H NMR spectrum of **9**.

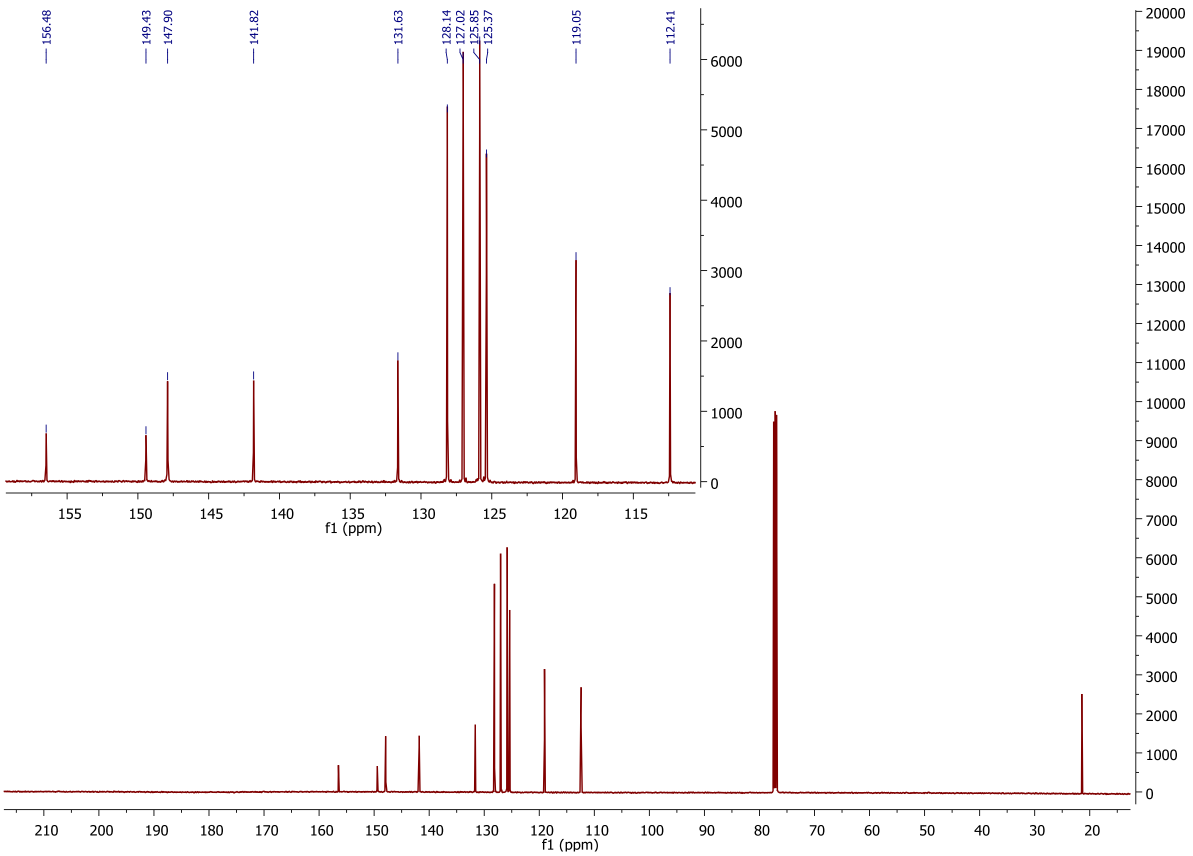

Figure S65: ^{13}C NMR spectrum of **9**.

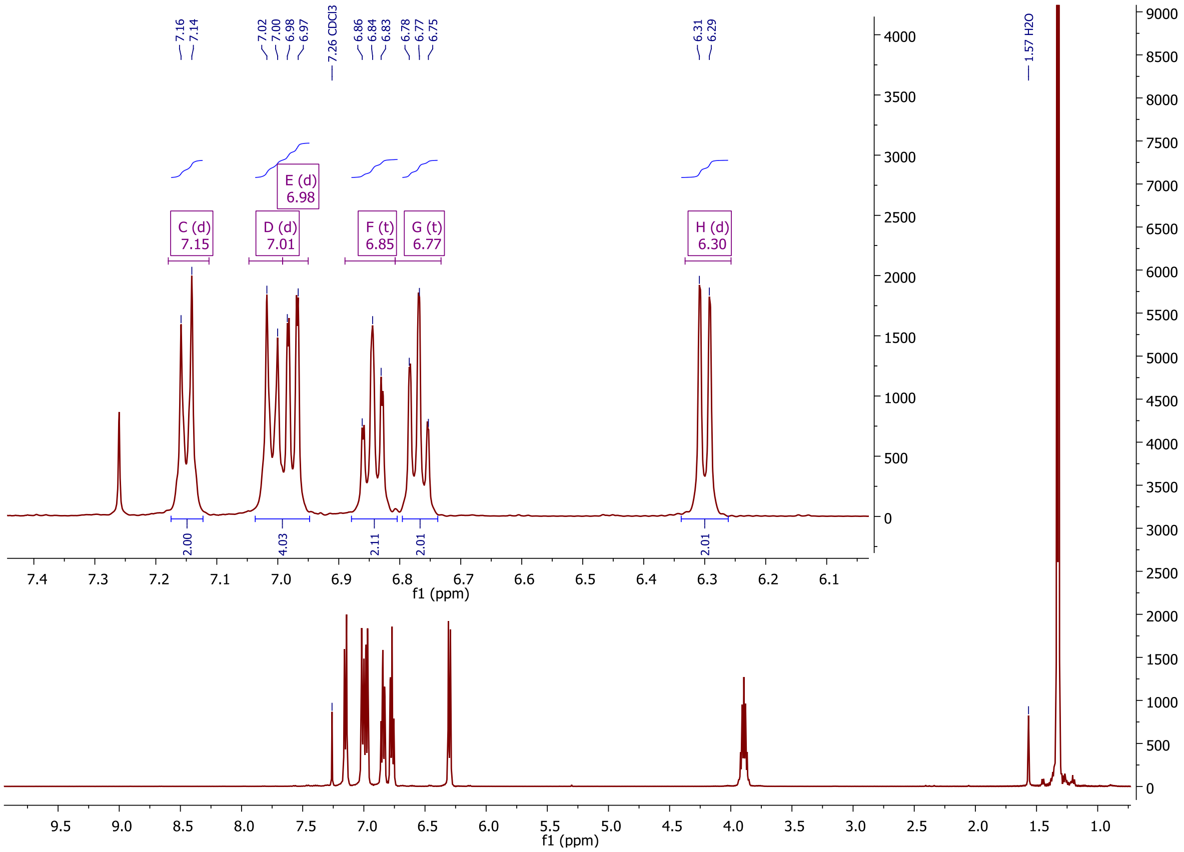

Figure S66: ^{1}H NMR spectrum of **10**.

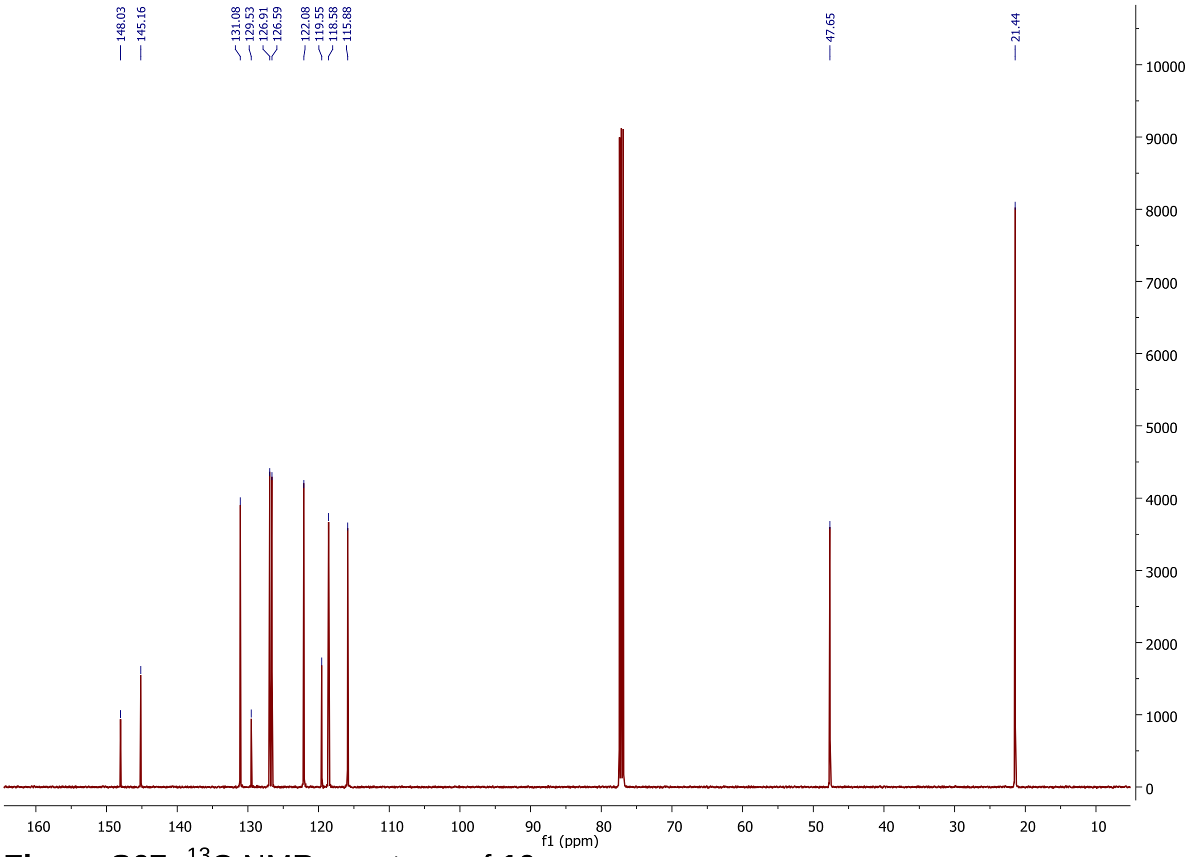

Figure S67: ^{13}C NMR spectrum of **10**.

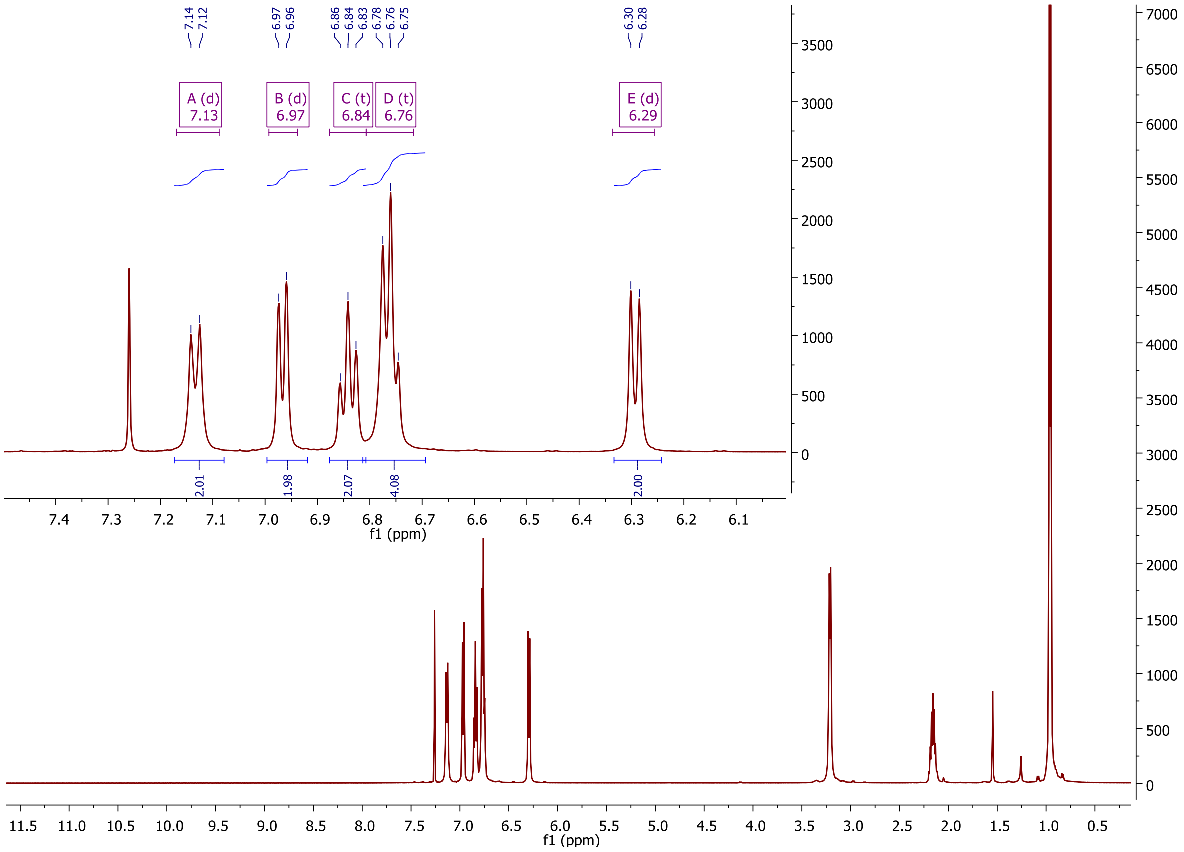

Figure S68: ^{1}H NMR spectrum of **11**.

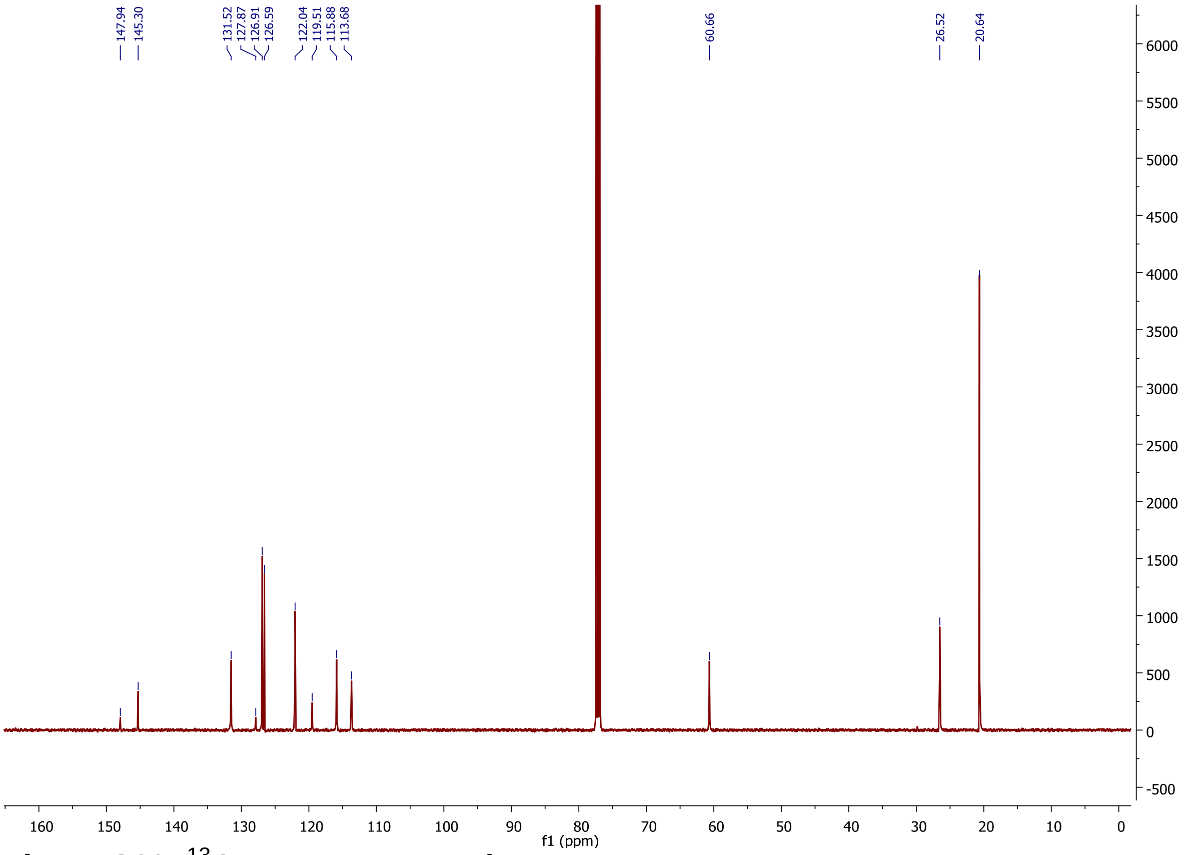

Figure S69: ^{13}C NMR spectrum of **11**.

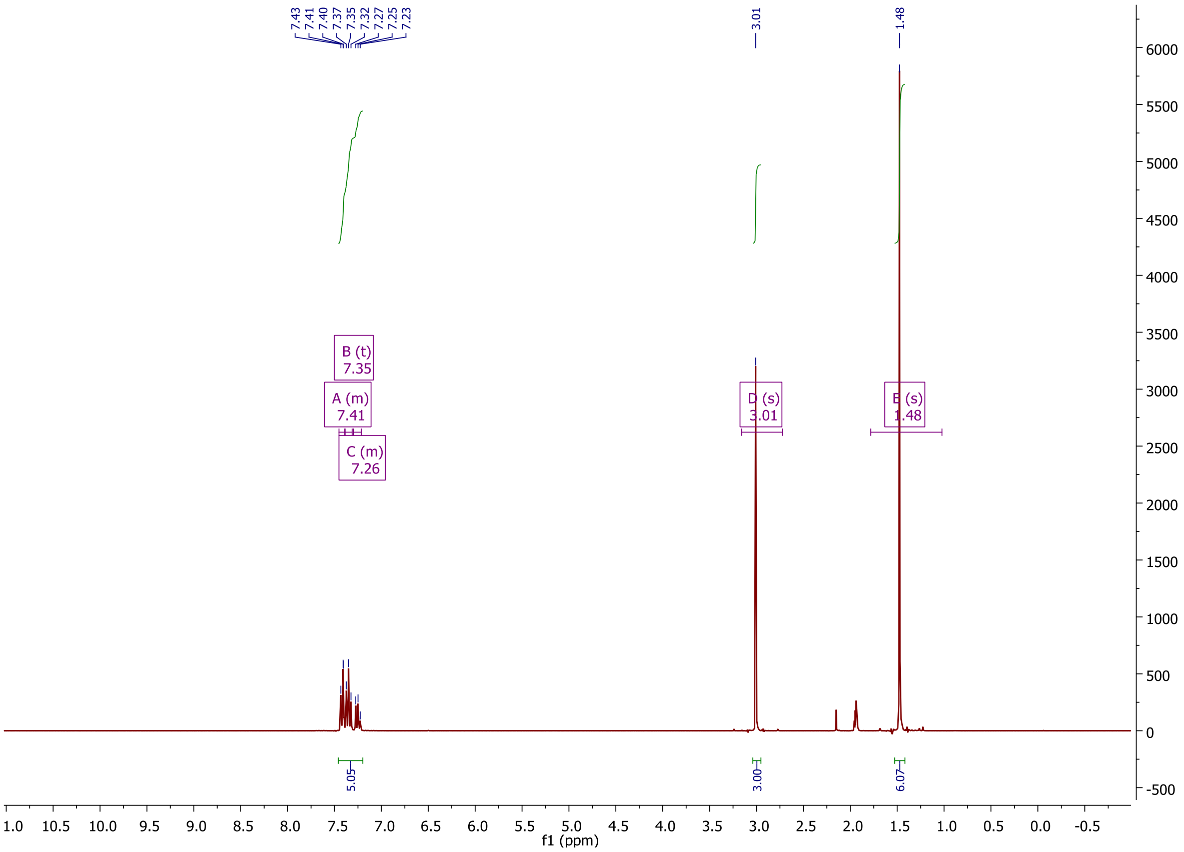

Figure S70: ^{1}H NMR spectrum of **16**.

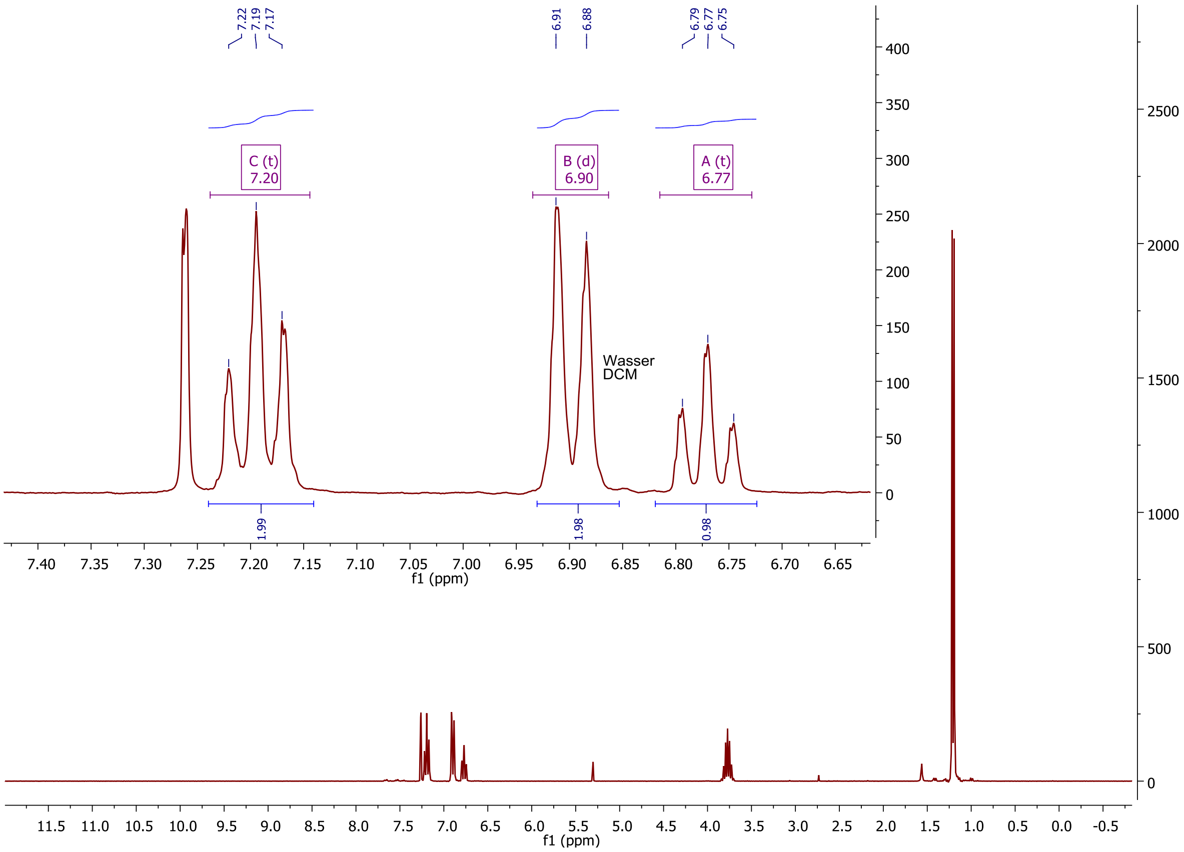

Figure S71: ^{1}H NMR spectrum of **17**.

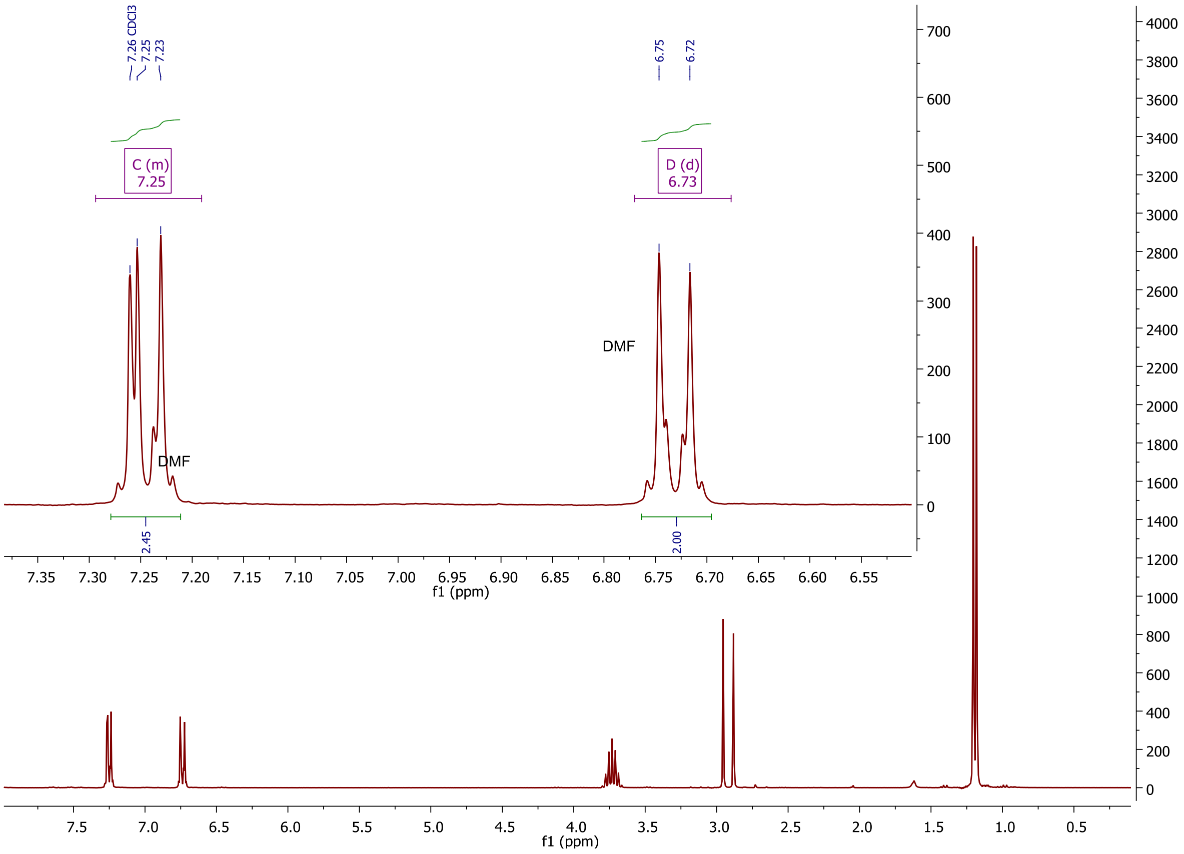

Figure S72: ^{1}H NMR spectrum of **18**.

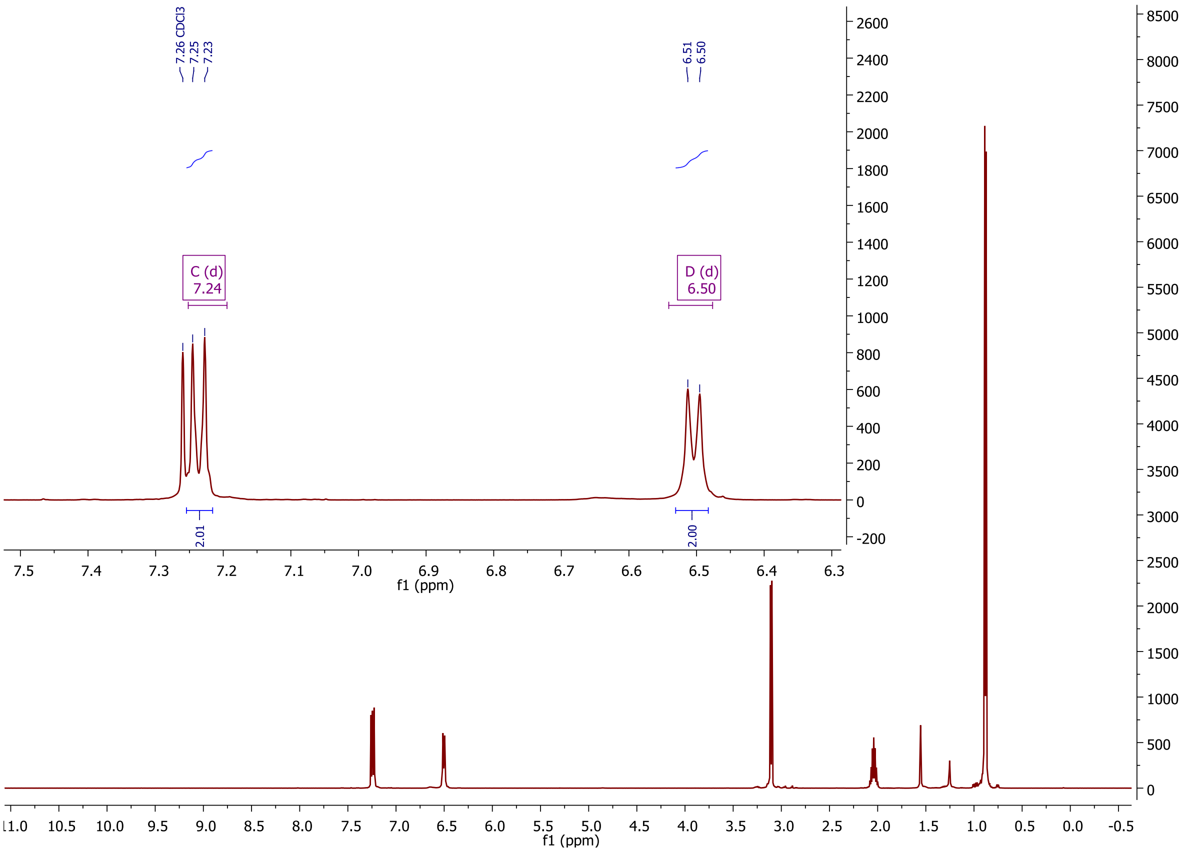

Figure S73: ^{1}H NMR spectrum of **19**.

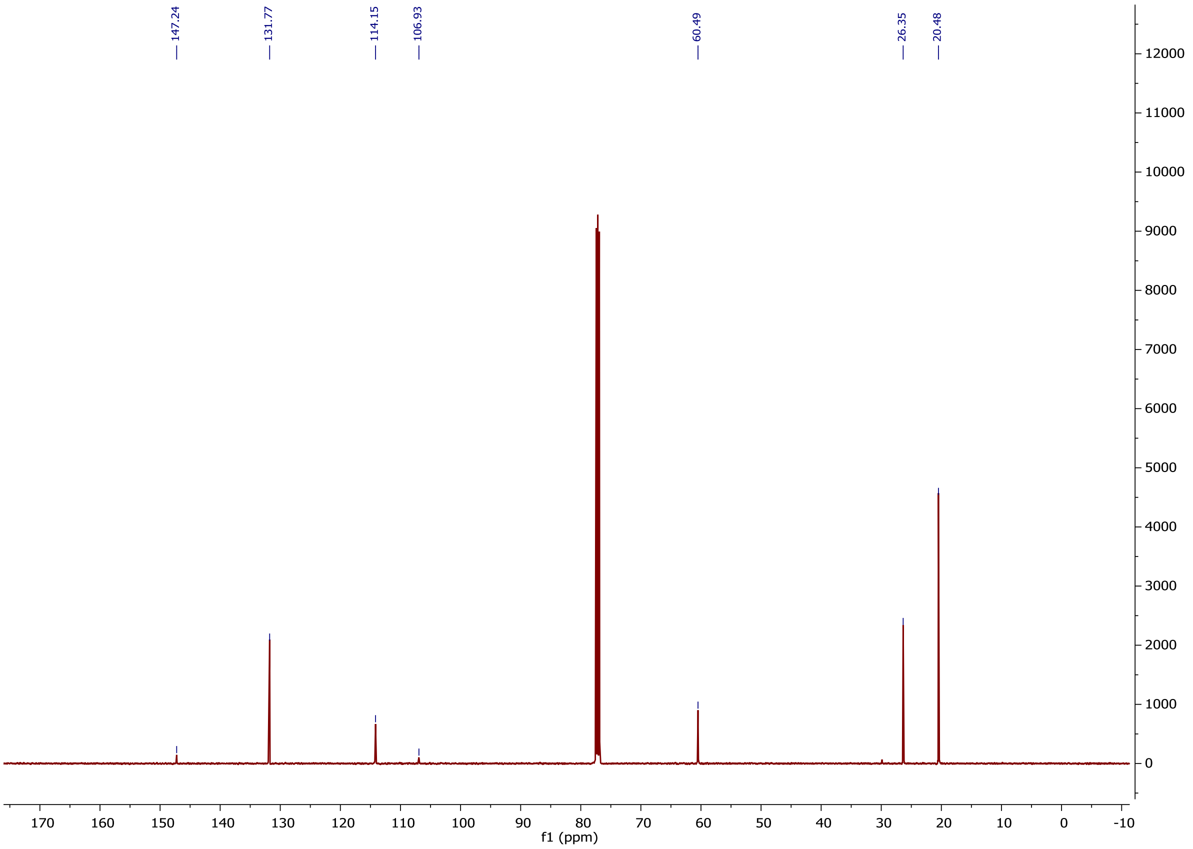

Figure S74: ^{13}C NMR spectrum of **19**.

Mass spectrometric data

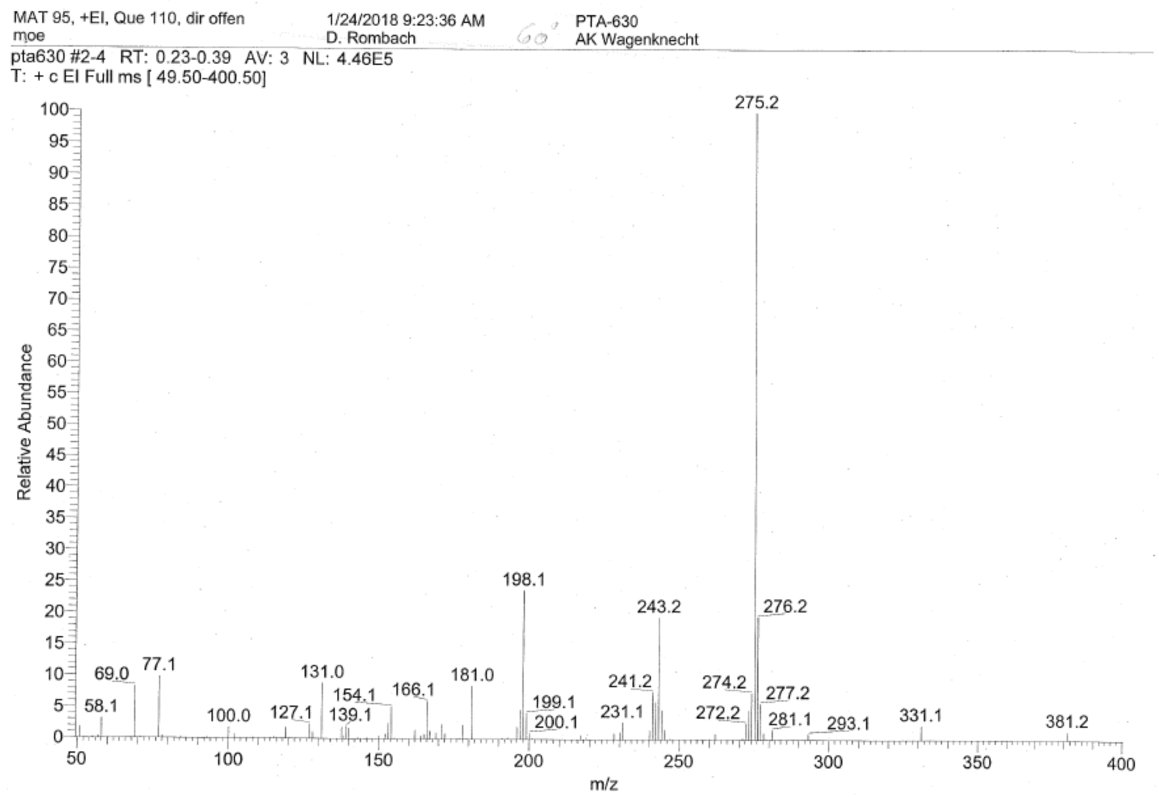

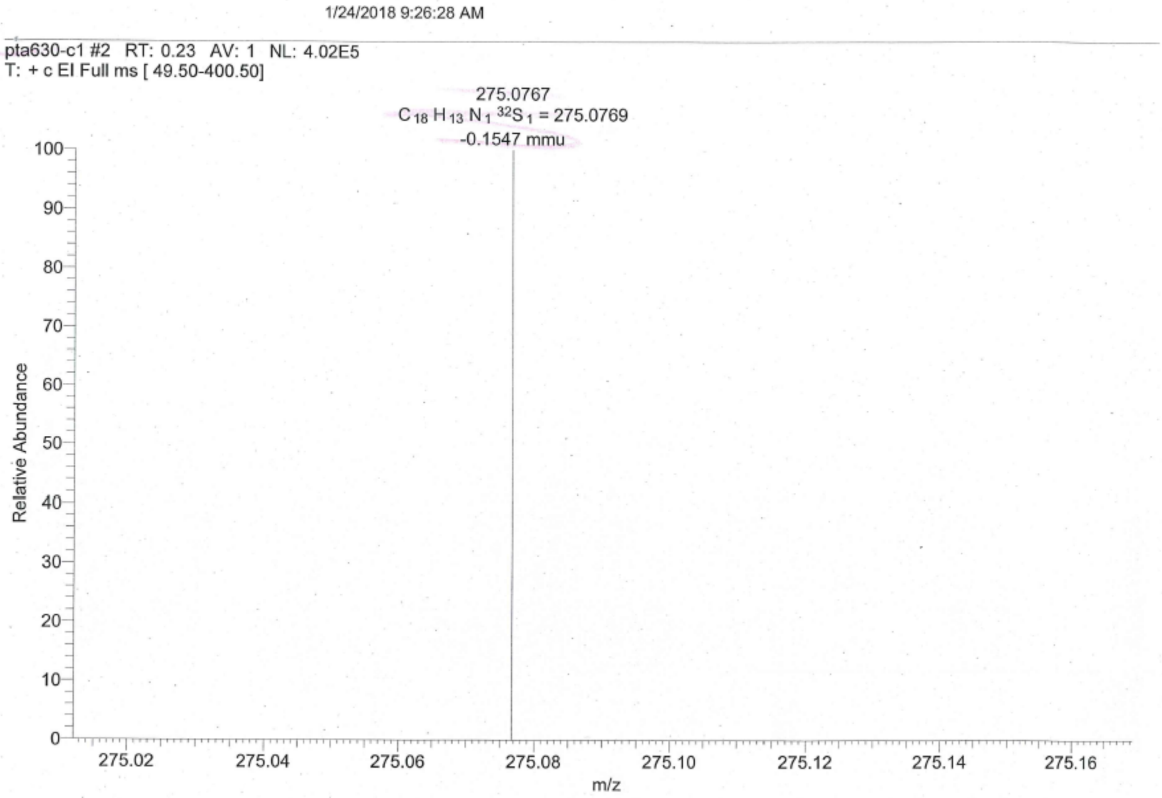

Figure S75: HR-EI/MS of **1**.

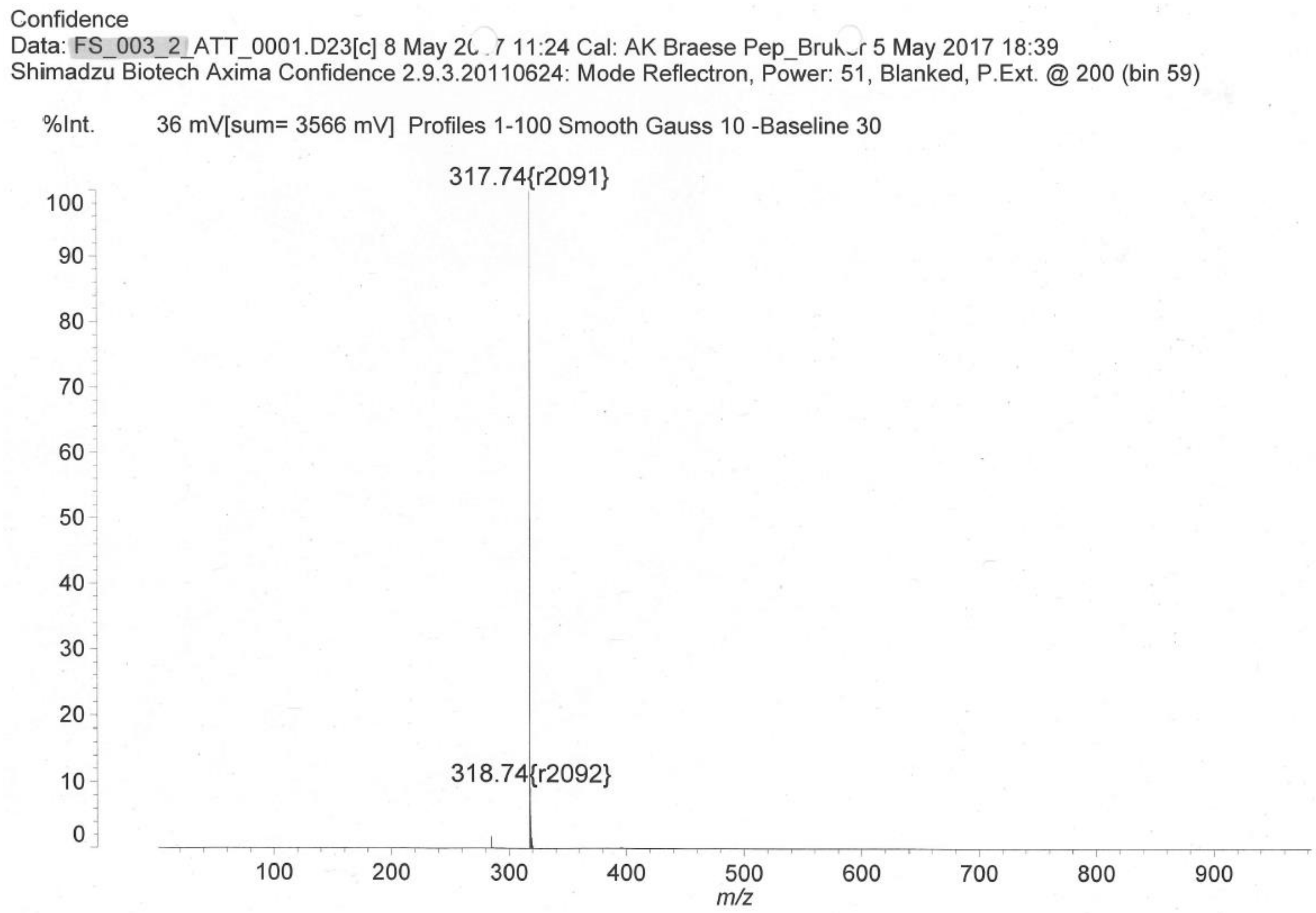

Figure S76: MALDI-TOF-MS of **2**..

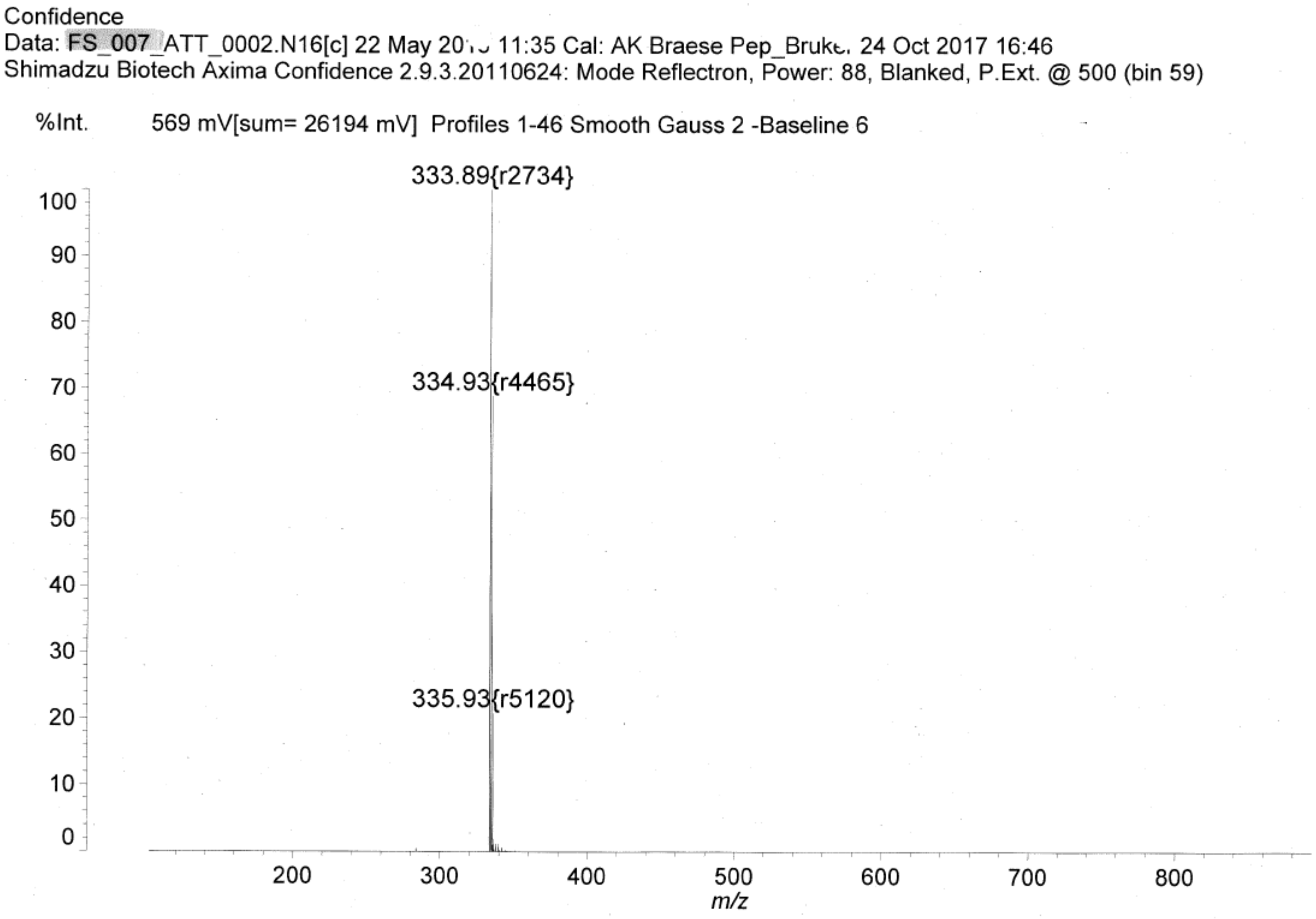

Figure S77: MALDI-TOF-MS of **3**.

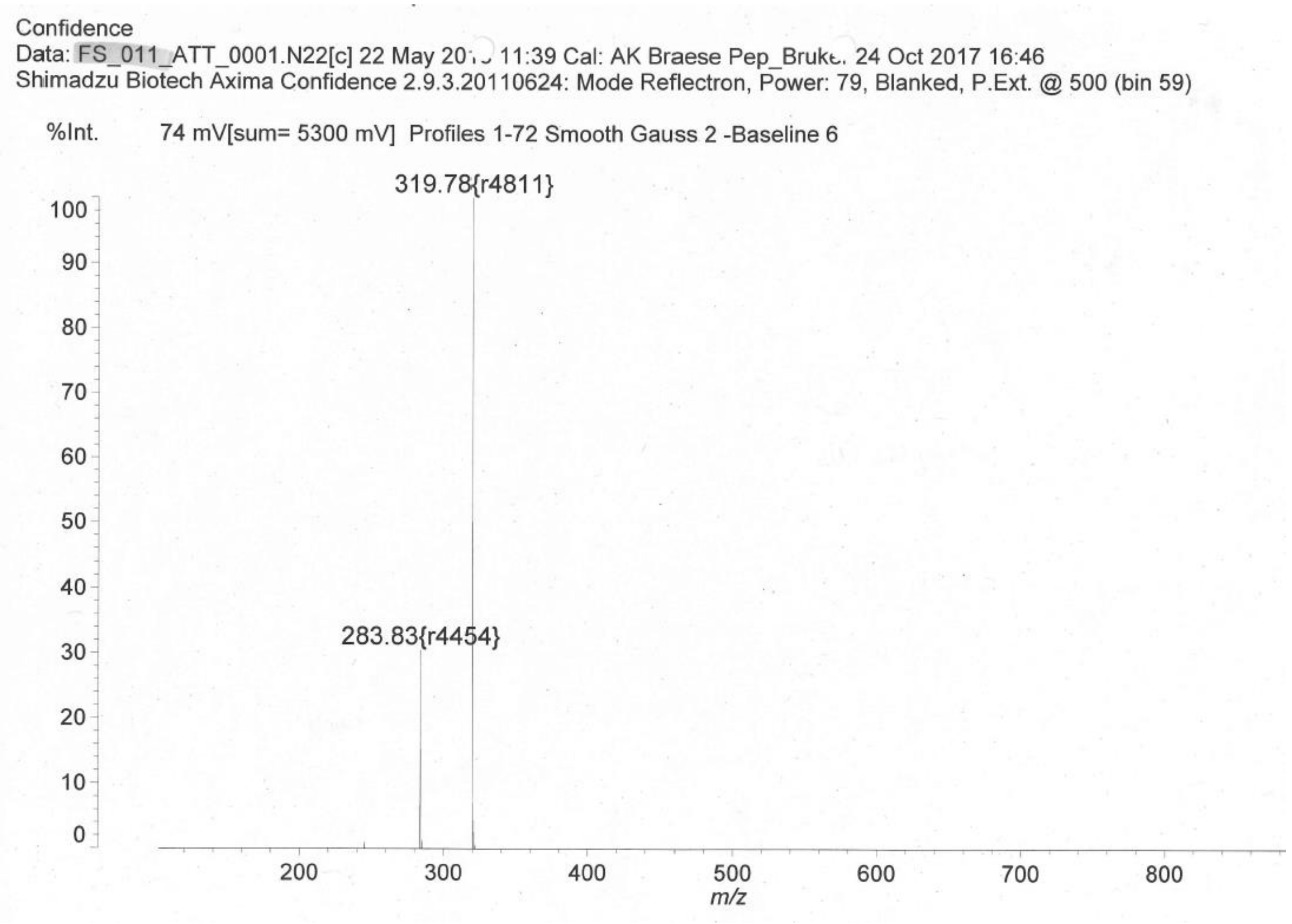

Figure S78: MALDI-TOF-MS of **4**.

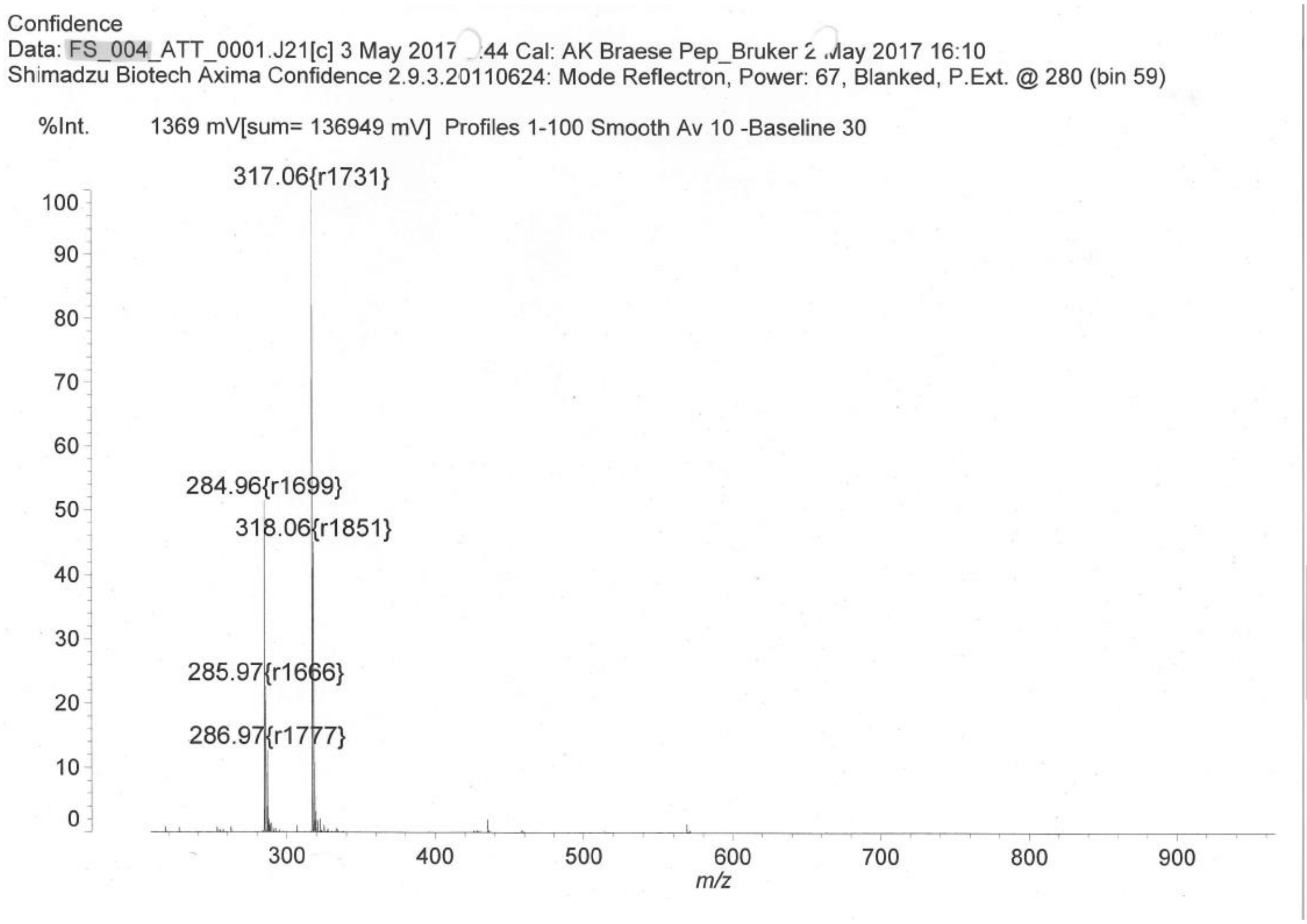

Figure S79: MALDI-TOF-MS of **5**.

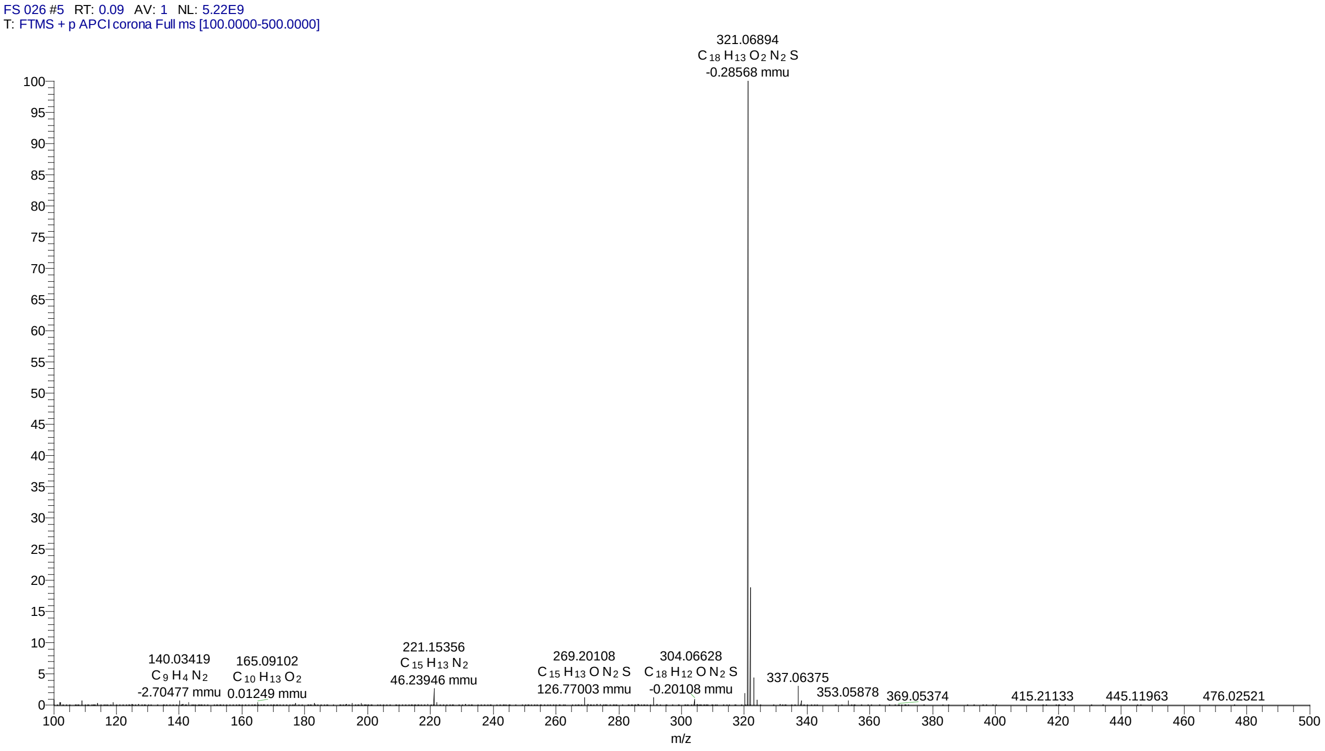

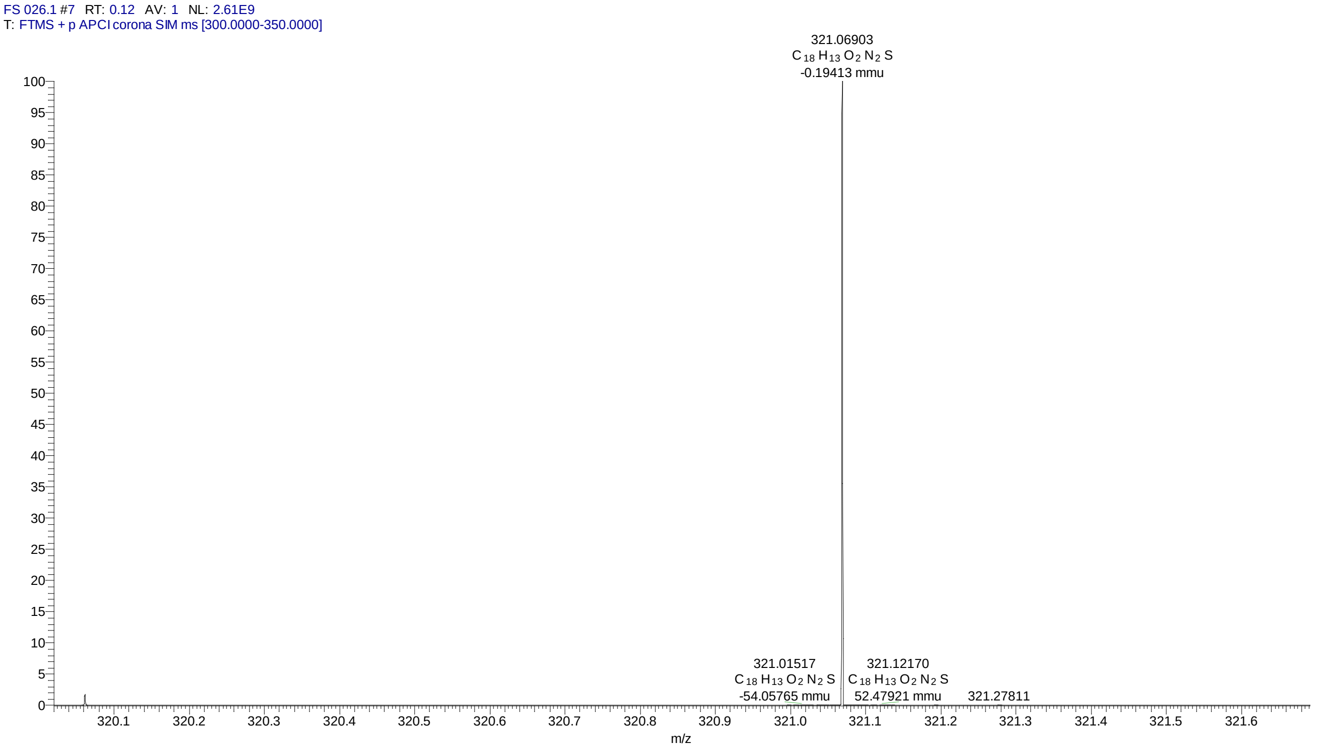

Figure S80: HR-APCI-MS of **6**.

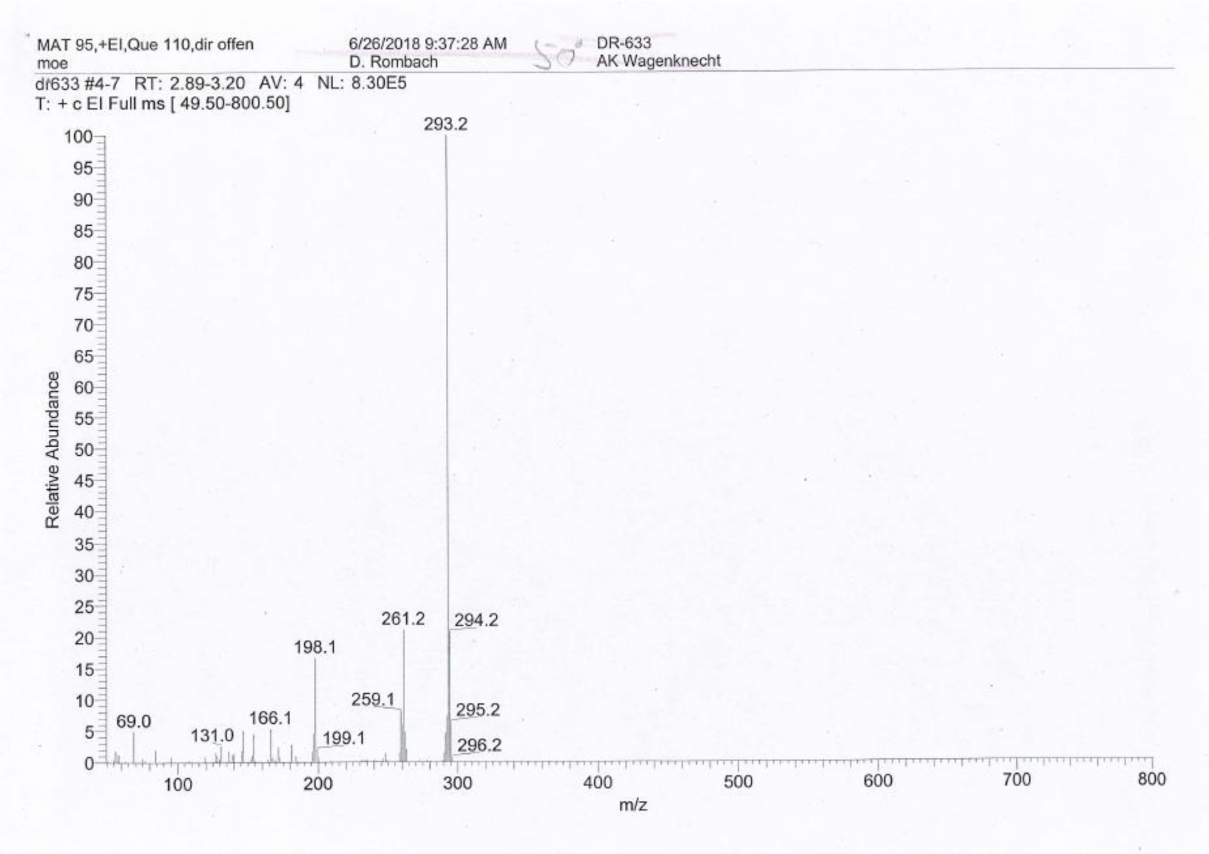

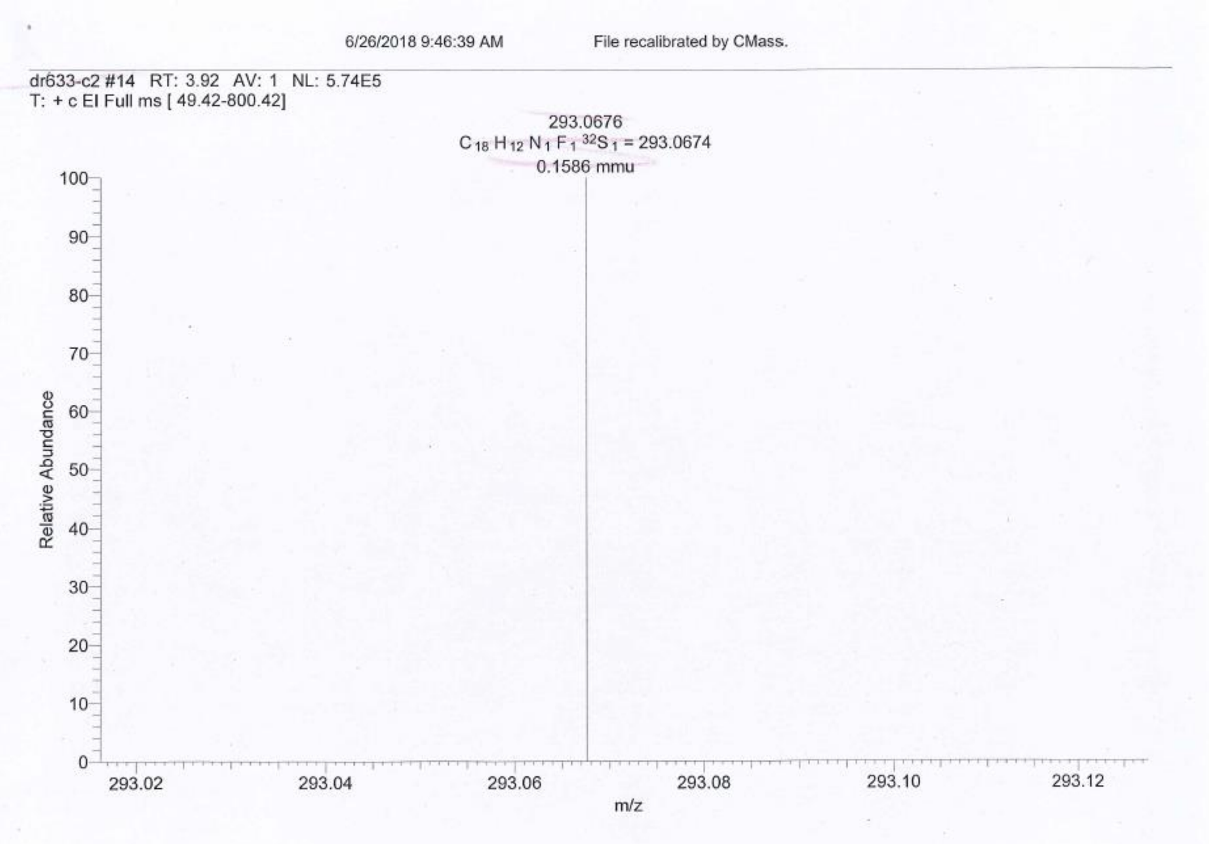

Figure S81: HR-EI-MS of **7**.

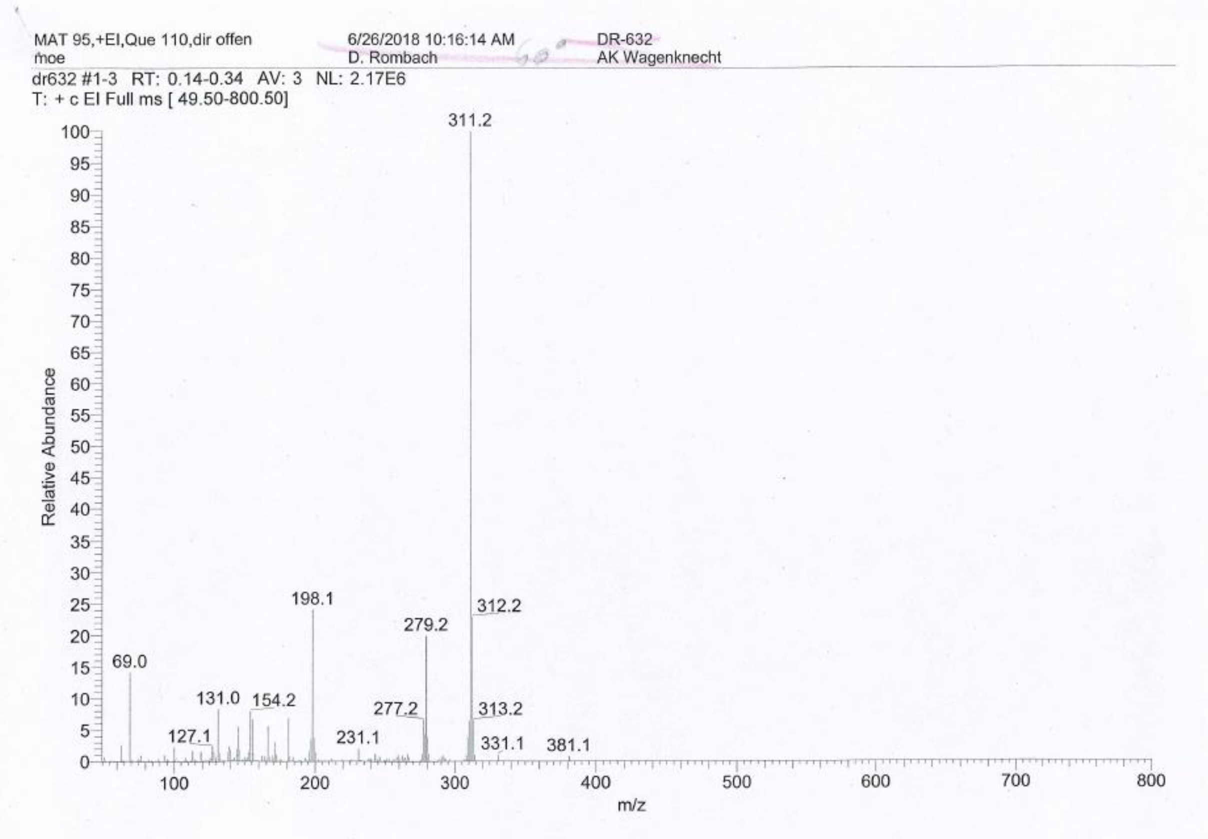

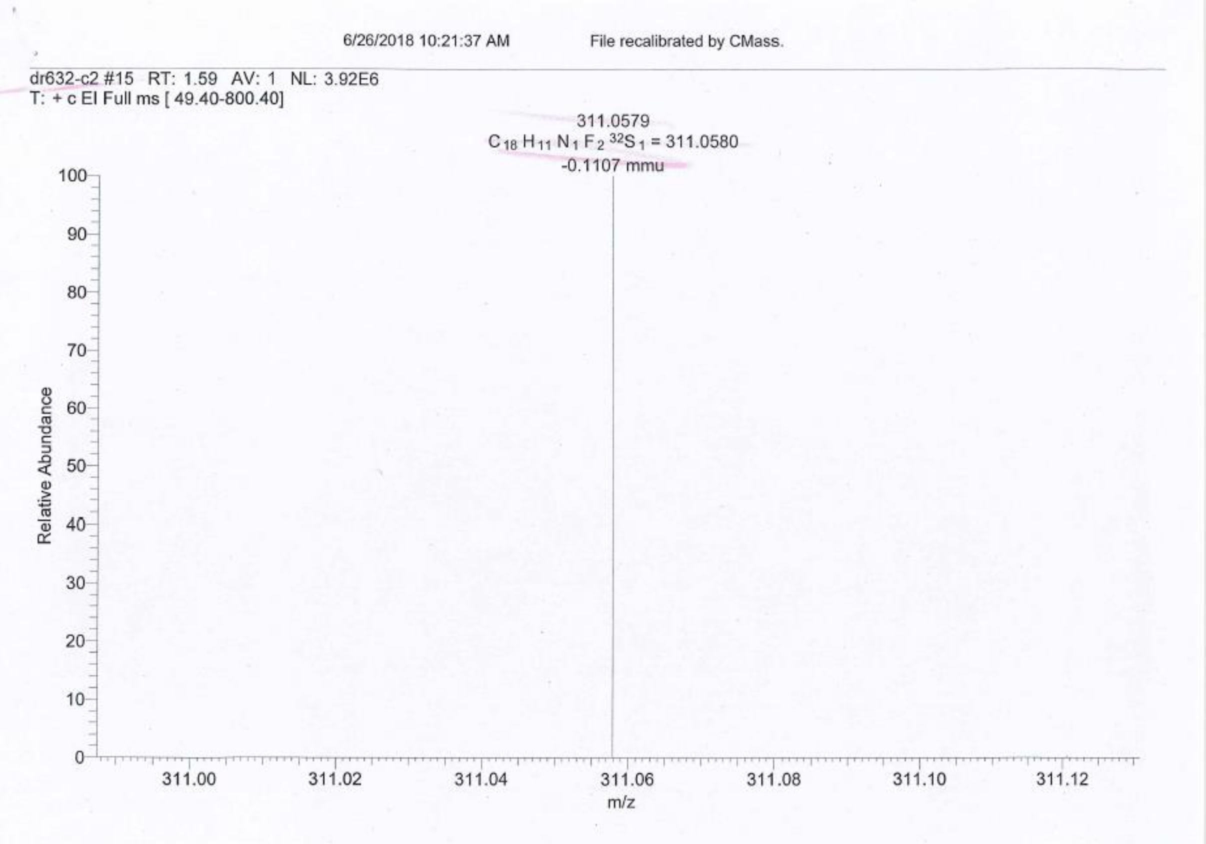

Figure S82: HR-EI-MS of **8**.

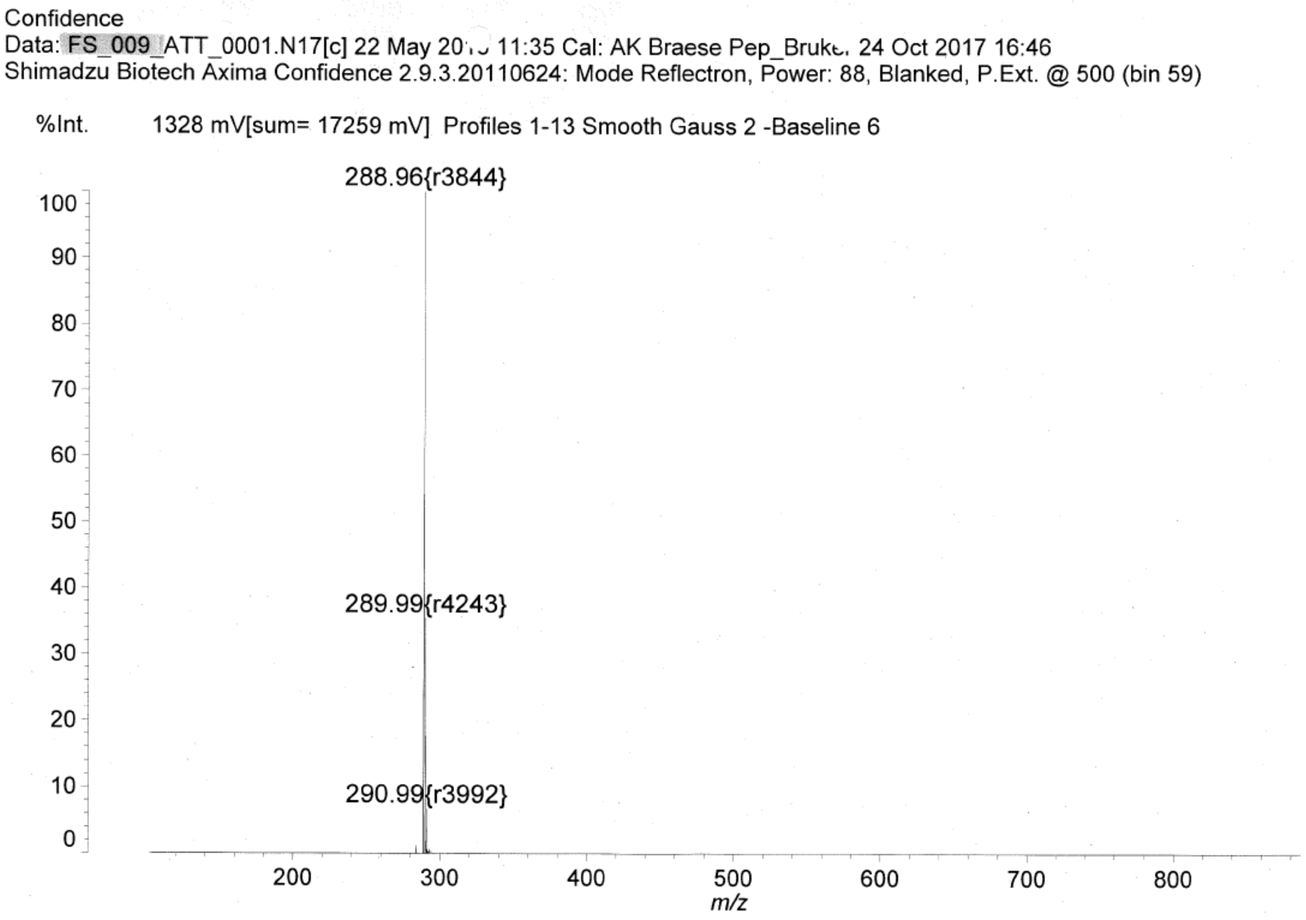

Figure S83: MALDI-TOF-MS of **9**.

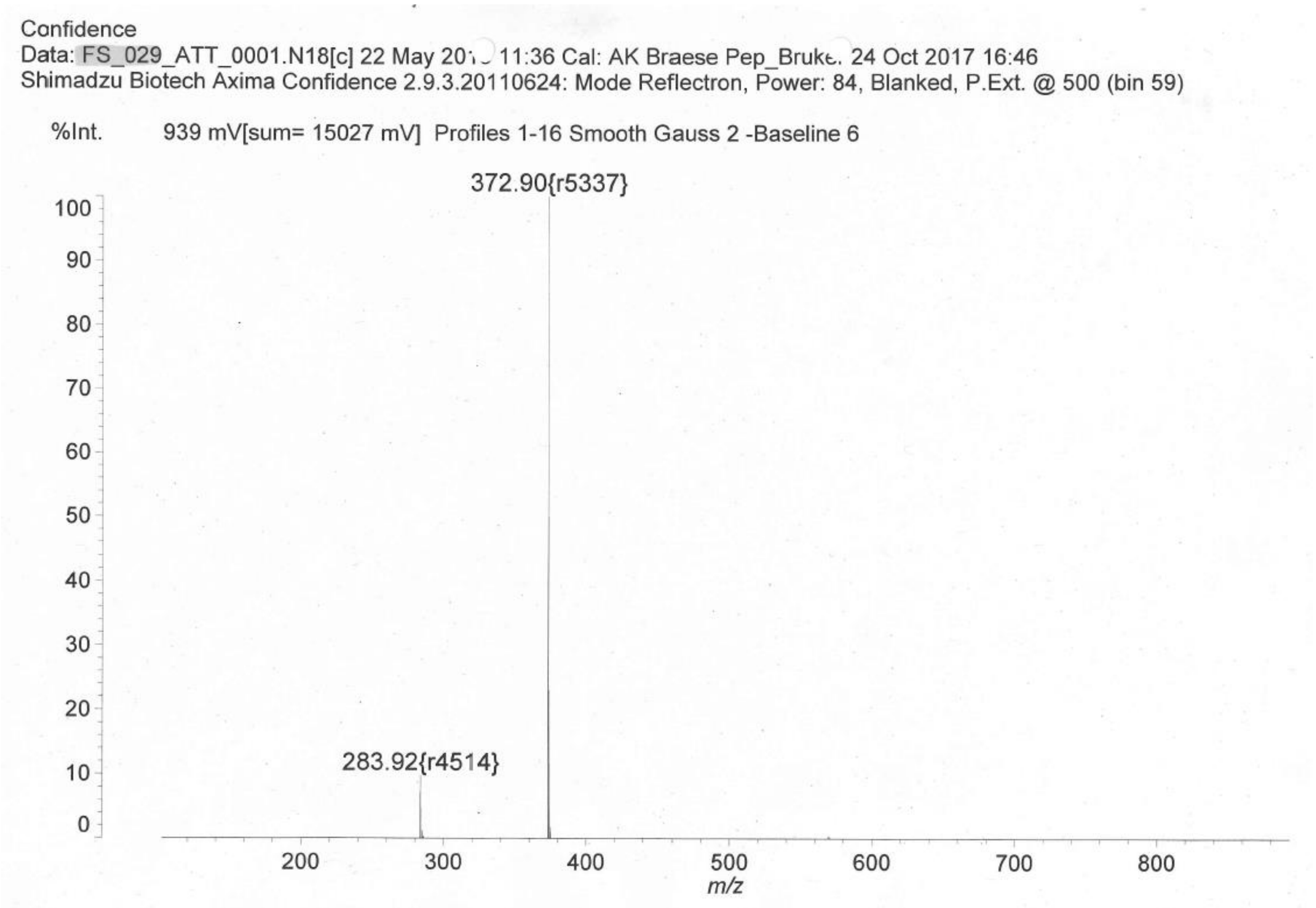

Figure S84: MALDI-TOF-MS of **10**.

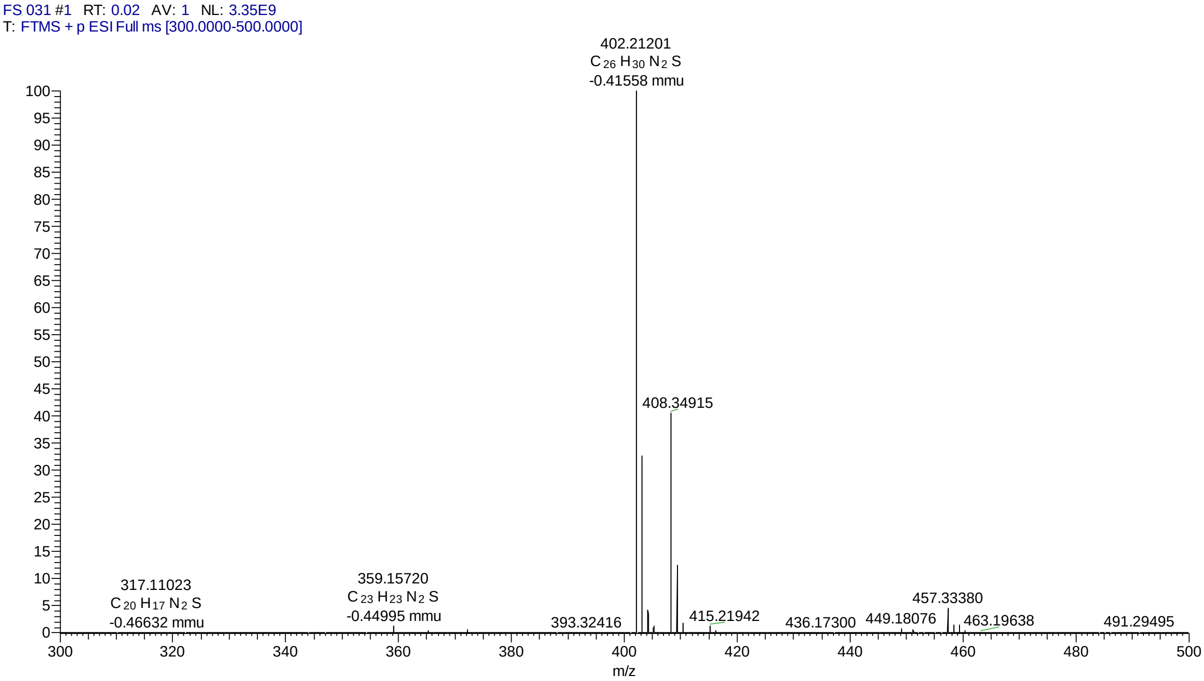

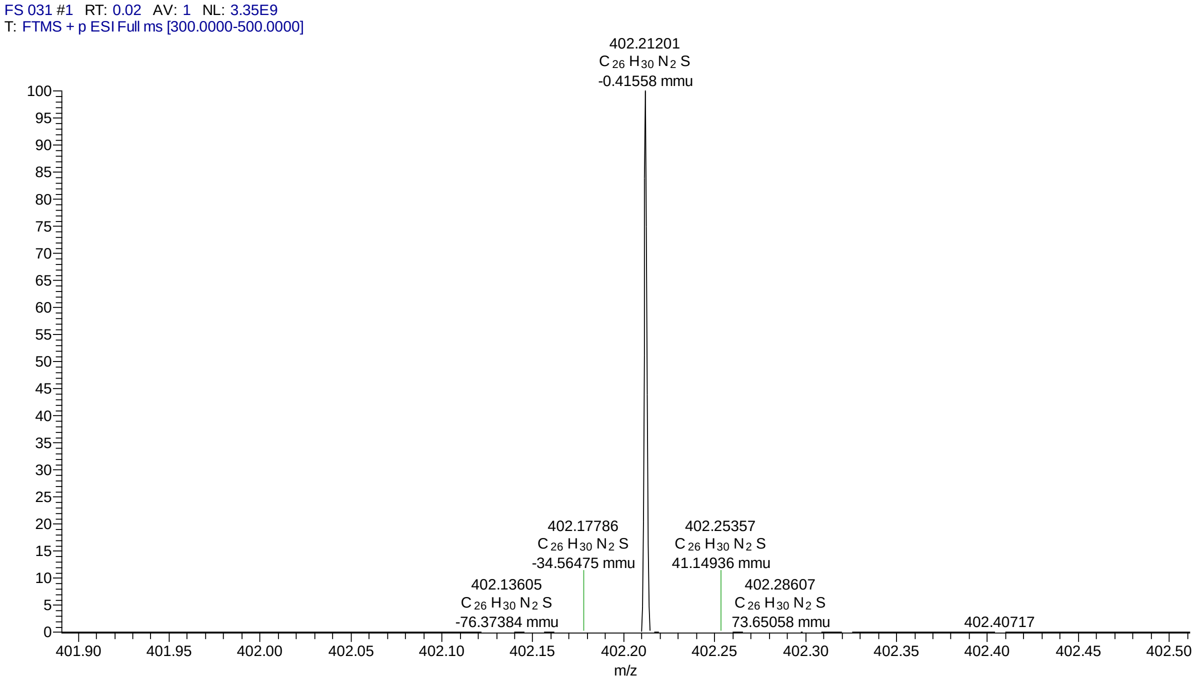

Figure S85: HR-ESI-MS of **11**.

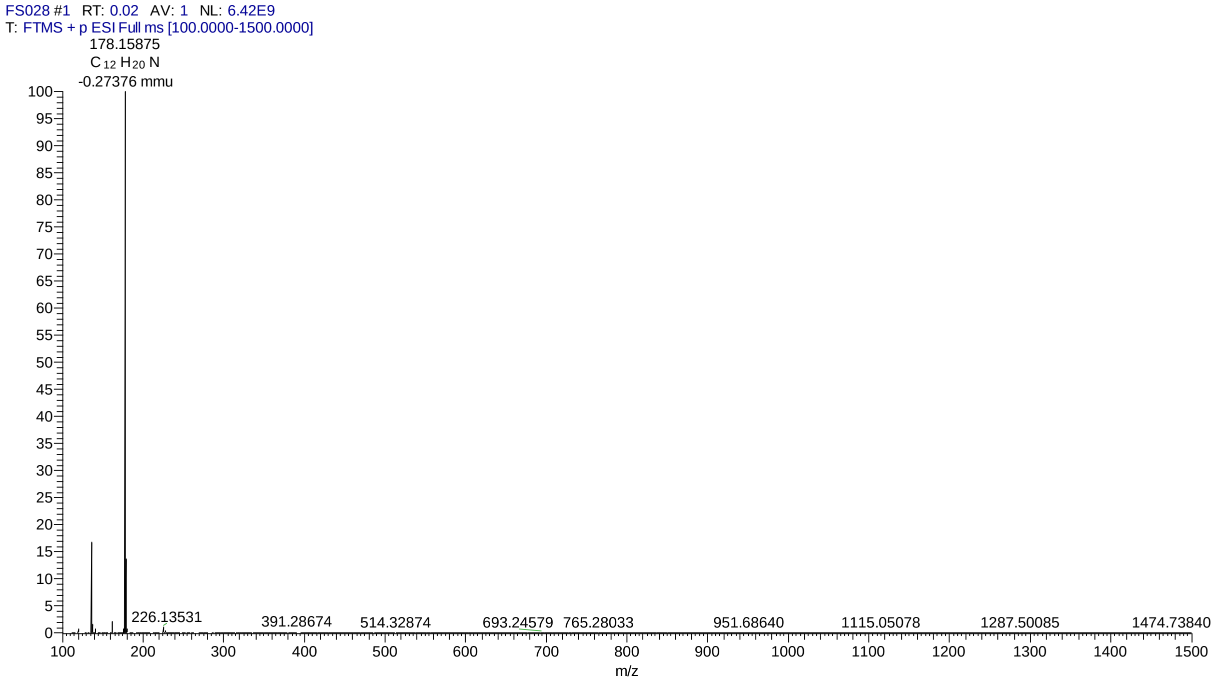

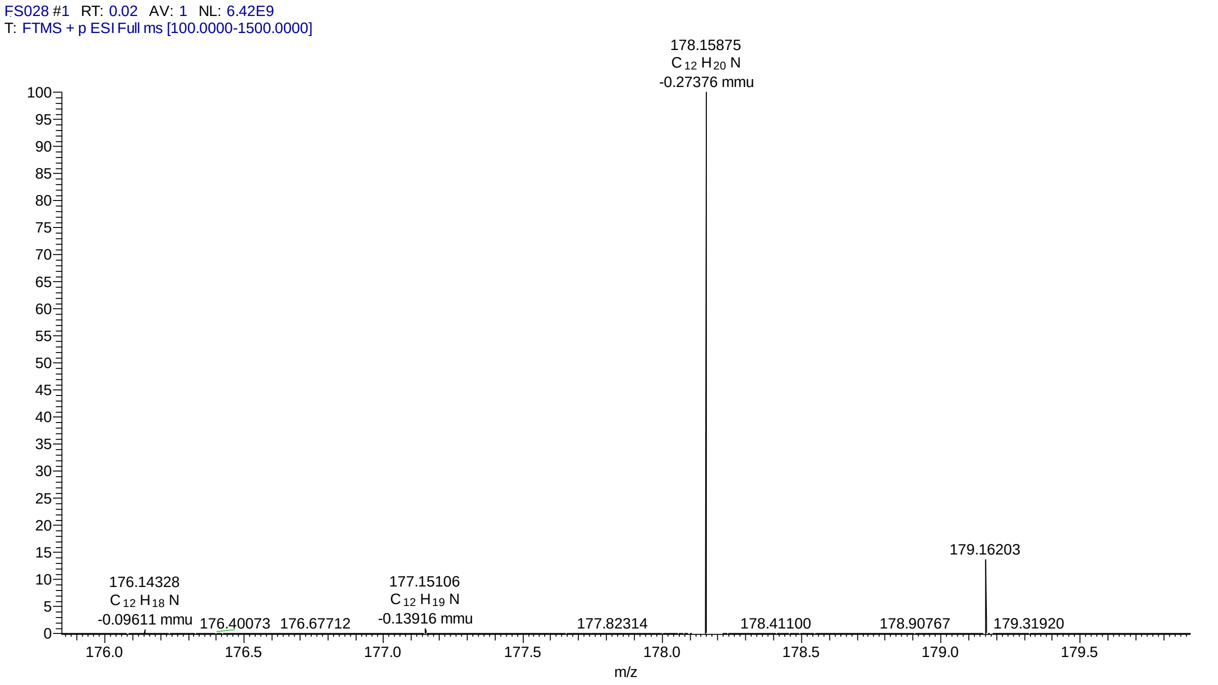

Figure S86: HR-ESI-MS of **17**.

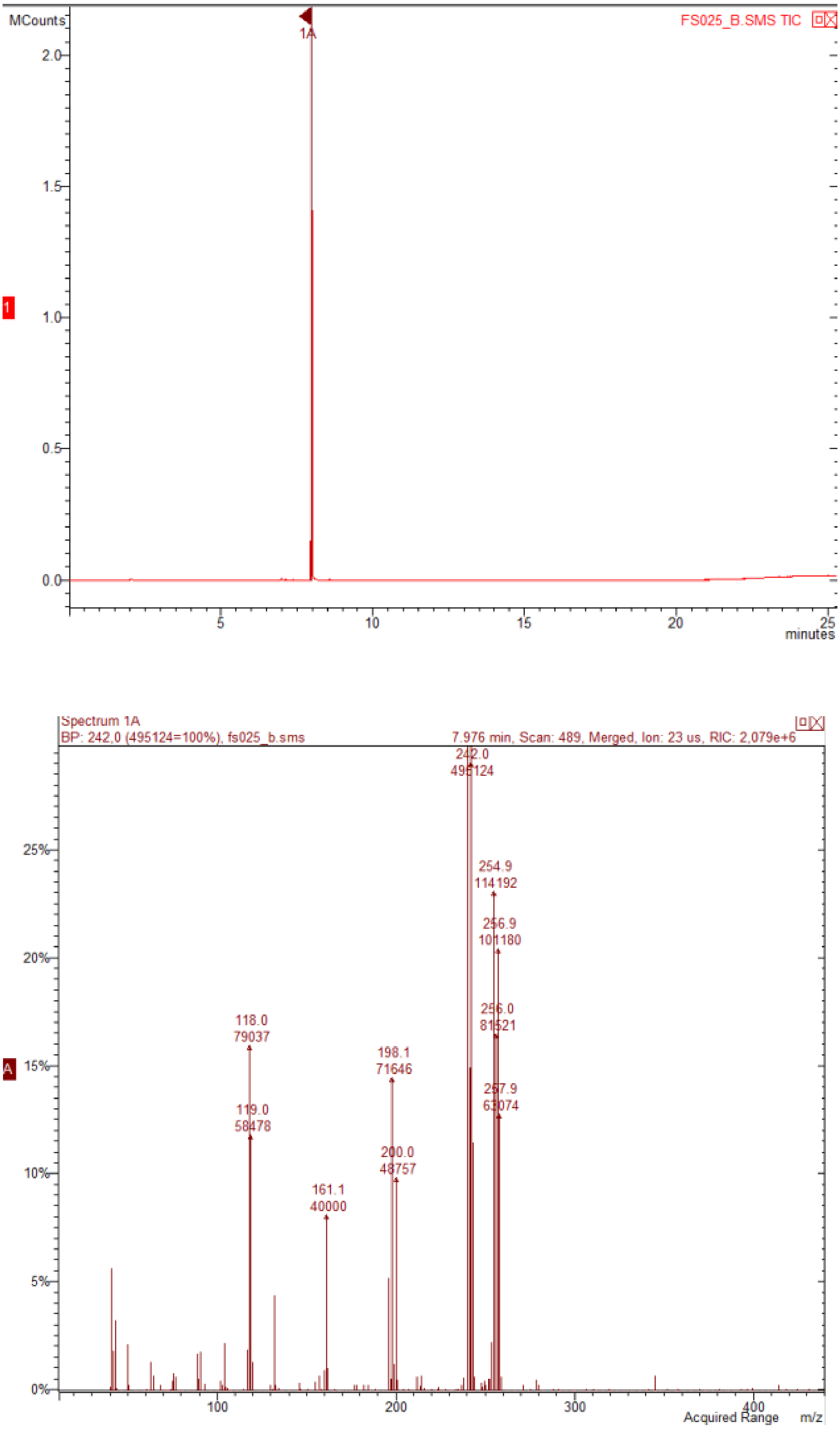

Figure S87: GC-EI-MS of **18**.

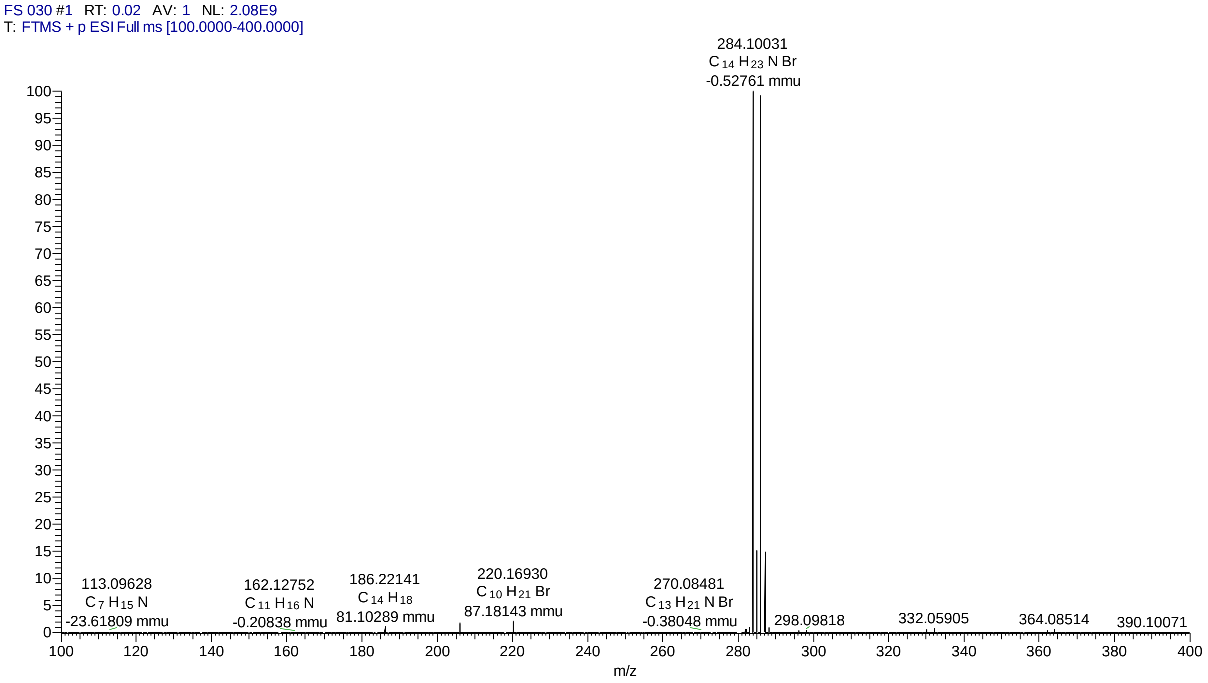

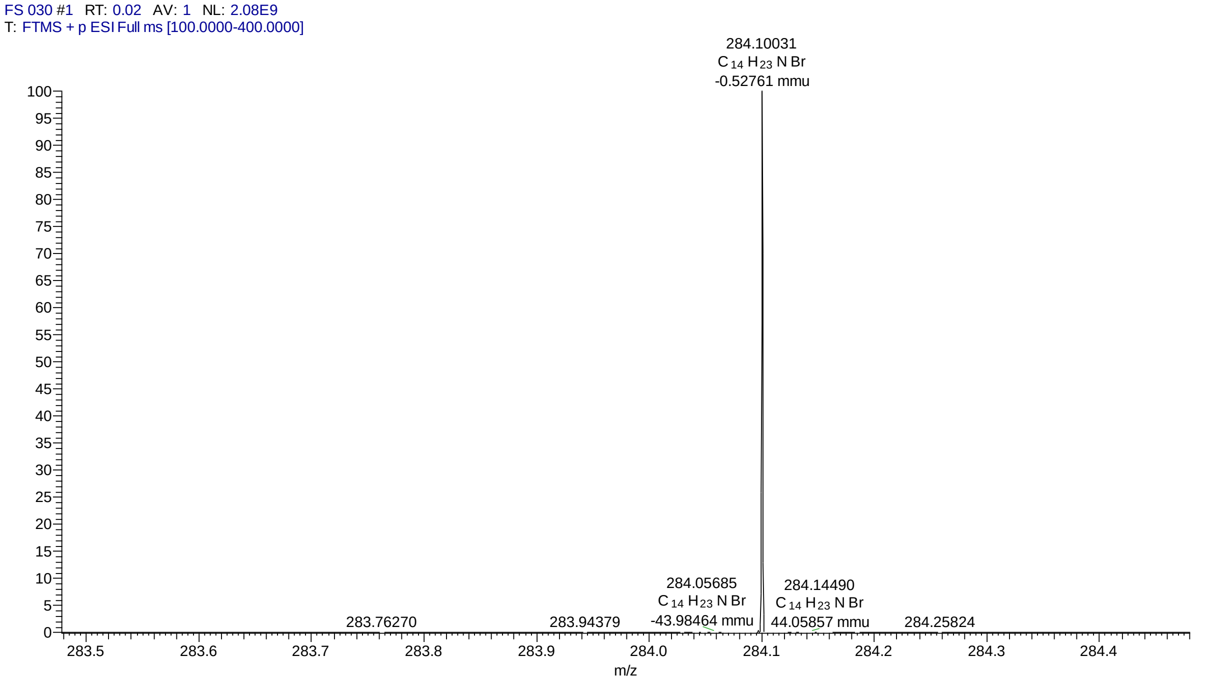

Figure S88: HR-ESI-MS of **19**.

References

S1. Fulmer, G.R.; Miller, A.J.M.; Sherden, N.H.; Gottlieb, H.E.; Nudelman, A.; Stoltz, B.M.; Bercaw J.B.; Goldberg, K.I. *Organometallics* **2010**, *29*, 2176–2179.

S2. Cheng, Y.-J.; Yu, S.-Y.; Lin, S.-C.; Lin, J.T.; Chen, L.-Y.; Hsiu, D.-S.; Wen, Y.S.; Lee, M.M.; Sun, S.-S. *J. Mater. Chem. C*, **2016**, *4*, 9499.

S3. Rombach,D.; Wagenknecht, H.-A. *ChemCatChem* **2018,** *10*, 2955–2961.

S4. Chen, M.; Deng, S.; Gu, Y.; Lin, J.; MacLeod, M.J.; Johnson, J.A. *J. Am. Chem. Soc*., **2017**, *139*, 2257 – 2266.

S5. Wang, D.; Talipov, M.R.; Ivanov, M.V.; Rathore, R. *J. Am. Chem. Soc*. **2016**, *138*, 16337 – 16344.
